# Influence of Remifentanil on the Pharmacokinetics and Pharmacodynamics of Remimazolam in Healthy Volunteers

**DOI:** 10.1097/ALN.0000000000005348

**Published:** 2025-01-15

**Authors:** Remco Vellinga, Jeroen V. Koomen, Douglas J. Eleveld, Thomas Stöhr, Marija Pesic, Michel M. R. F. Struys, Pieter J. Colin

**Affiliations:** 1 Department of Anesthesiology, University of Groningen, University Medical Center Groningen, Groningen, The Netherlands.; 2 Department of Anesthesiology, University of Groningen, University Medical Center Groningen, Groningen, The Netherlands; Department of Pharmacology, Toxicology and Kinetics, Dutch Medicines Evaluation Board, Utrecht, The Netherlands.; 3 Department of Anesthesiology, University of Groningen, University Medical Center Groningen, Groningen, The Netherlands.; 4 Department of Research and Development, Paion Deutschland GmbH, Aachen, Germany.; 5 Paion Deutschland GmbH, Aachen, Germany.; 6 Department of Anesthesiology, University of Groningen, University Medical Center Groningen, Groningen, The Netherlands; Department of Basic and Applied Medical Sciences, Ghent University, Ghent, Belgium.; 7 Department of Anesthesiology, University of Groningen, University Medical Center Groningen, Groningen, The Netherlands.

## Abstract

**Background:**

Synergistic effects between opioids and remimazolam on Bispectral Index (BIS) and Modified Observer’s Assessment of Alertness and Sedation (MOAAS) score were previously described. This study aimed to characterize the influence of remifentanil on the sedative properties of remimazolam as measured by MOAAS, BIS, and tolerance to laryngoscopy or tetanic stimulation (TOL or TOTS) and to determine target concentrations that maximize MOAAS 2 or 3.

**Methods:**

A three-period, crossover, dose-ranging clinical trial was performed in 24 healthy volunteers. In all periods, remimazolam was administered using a step-up and step-down target controlled infusion protocol (50 to 2,000 ng/ml). Stable remifentanil target concentrations of 0.5 ng/ml and 0.1 to 4.0 ng/ml were maintained in periods 2 and 3, respectively. Remifentanil, remimazolam, and CNS7054 (metabolite) concentrations and MOAAS, BIS, TOL, and TOTS were collected in each step of the target controlled infusion protocol. Data were analyzed using nonlinear mixed-effects models, where *P* ≤ 0.01 was considered significant.

**Results:**

Remifentanil reduced the apparent clearance of CNS7054 with a half-maximum inhibition at 8.0 ng/ml (95% CI, 5.5 to 13.4 ng/ml). A pharmacodynamic interaction was detected on all endpoints. Simulations indicate that the probability of observing a MOAAS 2 or 3 is highest at remimazolam target concentration of 275, 250, or 200 ng/ml combined with 0, 0.1, or 0.5 ng/ml remifentanil resulting in probabilities of 45%, 45%, and 44%, respectively. Additionally, simulations indicate that the highest probability of observing TOTS and TOL was 93.3% and 85.5%, respectively, at the highest studied target concentrations.

**Conclusions:**

A pharmacokinetic and pharmacodynamic drug–drug interaction between remimazolam and remifentanil was quantified in this clinical trial. Appropriate target concentrations for MOAAS and BIS could be estimated, but for TOL and TOTS, the trial design did not allow to fully characterize the exposure–response relationship.

## Abstract 2

The effect of coadministration of remifentanil on the sedative properties of remimazolam, as measured by Modified Observer’s Assessment of Alertness and Sedation (MOAAS) scores, Bispectral Index, tolerance to tetanic stimulation, and tolerance to laryngoscopy, was assessed in 24 healthy volunteers in a three-period, crossover, dose-ranging trial. Coadministration of remifentanil had no effect on the elimination clearance of remimazolam but inhibited the apparent elimination clearance of its metabolite, CNS7054, and had a pharmacodynamic interaction with remimazolam at all endpoints. Simulations predicted the probability of a MOAAS score of 2 or 3 was 45%, 45%, and 44% for a remimazolam target concentration of 275, 250, or 200 ng/ml, respectively, combined with 0, 0.1, or 0.5 ng/ml remifentanil, respectively.

## Editor’s Perspective

**What We Already Know about This Topic**

- Remimazolam is a short-acting benzodiazepine that is administered as repeated bolus doses for procedural sedation and as a continuous infusion for general anesthesia in adults
- Coadministration of opioids affects the sedative effects of remimazolam

**What This Article Tells Us That Is New**

- The effect of coadministration of remifentanil on the sedative properties of remimazolam, as measured by Modified Observer’s Assessment of Alertness and Sedation (MOAAS) scores, Bispectral Index, tolerance to tetanic stimulation, and tolerance to laryngoscopy, was assessed in 24 healthy volunteers in a three-period, crossover, dose-ranging trial
- Coadministration of remifentanil had no effect on the elimination clearance of remimazolam but inhibited the apparent elimination clearance of its metabolite, CNS7054, and had a pharmacodynamic interaction with remimazolam at all endpoints
- Simulations predicted the probability of a MOAAS score of 2 or 3 was 45%, 45%, and 44% for a remimazolam target concentration of 275, 250, or 200 ng/ml, respectively, combined with 0, 0.1, or 0.5 ng/ml remifentanil, respectively

Remimazolam exhibits sedative properties by binding to the γ-aminobutyric acid type A receptor.^1,2^ Remimazolam is administered *via* intermittent bolus dosing or continuous infusion.^3,4^ The U.S. drug label states that the dose of remimazolam for procedural sedation was based on data from patients who received opioids before the first dose of remimazolam, which suggests remimazolam should be administered in presence of an opioid.^4^ In the European Union drug label, the dose of remimazolam for procedural sedation is dependent on concomitant administration of an opioid, where the dose is lower in the presence of an opioid.^3^ In contrast, for general anesthesia, it is less clear to what extent the dose of remimazolam depends on concomitant administration of an opioid.^3^

Synergistic effects between the opioid, remifentanil, and remimazolam on the Bispectral Index (BIS) and the opioid, fentanyl, and remimazolam on the Modified Observer’s Assessment of Alertness and Sedation (MOAAS) score have been previously described.^5,6^ Both analyses demonstrated that the magnitude of the synergistic effects on sedation outcomes between opioids and remimazolam are dependent on the opioid dose.

A warning of profound sedation during concomitant administration of an opioid is specified in both the European Union and U.S. drug labels of remimazolam, but a description of the magnitude of the synergistic effects between remimazolam and opioids is lacking.^3,4^ Additionally, in the European Union drug label, there is no specific guidance on adjusting the dose of remimazolam in the presence of opioids.^3^ Therefore, it is currently unclear how the recommended dose of remimazolam for procedural sedation and general anesthesia is affected by the administered opioid dose.

In a previous analysis, we showed that there is development of acute tolerance to the sedative properties of remimazolam when administered to healthy volunteers using target controlled infusion (TCI) in the absence of opioids, which is potentially mediated by the metabolite of remimazolam, CNS7054.^7^ As a consequence, the dose–exposure–response relationship between remimazolam and its sedative effects changes over time.^7^ The primary objective of our trial was to characterize the dose–exposure–response relationship of remimazolam when targeting MOAAS 2 or 3 and the influence of remifentanil on this relationship. In the current analysis, we aimed to quantify the influence of remifentanil on the sedative properties of remimazolam as measured by MOAAS, BIS, tolerance to tetanic stimulation (TOTS), and tolerance to laryngoscopy (TOL). In addition, we investigated whether there is also acute tolerance development in the presence of the opioid remifentanil. Finally, we aimed to determine appropriate remimazolam target concentrations in the presence of remifentanil for TCI.

## Materials and Methods

This trial was conducted at the Department of Anesthesiology of the University Medical Center Groningen, Groningen, The Netherlands (registered at https://clinicaltrials.gov with identifier NCT04670471). The independent Ethics Committee of the Foundation “Evaluation of Ethics in Biomedical Research” in Assen, The Netherlands, has approved the study. Written informed consent was obtained from all volunteers.

### Study Design

Details on inclusion and exclusion criteria, study design, and study procedures were described previously.^7^ In short, in this single-center, three-period, crossover, dose-ranging trial, healthy volunteers followed a step-up and step-down remimazolam TCI protocol combined with stable plasma remifentanil concentrations of 0.0, 0.5, and 2.0 or 4.0 ng/ml in periods 1, 2, and 3, respectively (Supplemental Digital Content fig. 1, https://links.lww.com/ALN/D786). Periods were separated by a washout period of 1 week. Healthy volunteers with an age between 18 and 70 yr, body mass index between 18 and 30 kg · m^-2^, a bilateral patent arteria radialis, and an American Society of Anesthesiologists (Schaumburg, Illinois) Physical Status of I were eligible for inclusion in this clinical trial. Upon inclusion, volunteers were stratified across groups (n = 6 groups) by age (18 to 34, 35 to 49, and 50 to 70 yr) and sex.

### Drug Administration

Drugs were administered by two syringe pumps (AlarisTM GH syringe pump, Becton Dickinson, USA) controlled by a computer running RUGLOOP II software (Demed, Belgium) for Windows (Microsoft, USA). The RUGLOOP II software was programed to deliver remimazolam (Byfavo, Paion, Germany) by effect-site TCI using the model developed by Zhou *et al.* with a rate constant for effect-site equilibration of 0.135 min^-1^.^5^ Remimazolam effect site target concentrations in the TCI protocol were 150, 300, 400, 800, 1,300, and 2,000 ng/ml during step-up and 1,300, 800, 400, 300, and 150 ng/ml during step-down in the first and second period; and 100, 200, 250, 500, 850, and 1,000 ng/ml during step-up and 850, 500, 250, 200, and 100 ng/ml during step-down for the low period 3 remifentanil target concentration or 75, 150, 200, 400, 650, and 1,000 ng/ml during step-up and 650, 400, 200, 150, and 75 ng/ml during step-down for the high period 3 remifentanil target concentration. For each step in the infusion protocol, the infusion duration was composed of the time to reach pharmacokinetic steady state, which depended on the rate constant for effect-site equilibration and targeted effect-site concentration and ranged between 2 and 30 min. This period was followed by an equilibration period of 10 min to ensure steady state remimazolam concentrations. Subsequently, blood samples for the measurement of plasma drug and metabolite concentrations and pharmacodynamic outcomes were collected over a period of approximately 10 min (Supplemental Digital Content figs. 2 and 3, https://links.lww.com/ALN/D786). Therefore, each step in the TCI protocol was planned to consist of at least 22 min and up to 50 min of administration of remimazolam.

The remifentanil model by Eleveld et al.^8^ was used to deliver remifentanil by plasma TCI in periods 2 and 3. In period 2, a plasma target concentration of 0.5 ng/ml and, in period 3, after randomization of healthy volunteers, a plasma target concentration of either 2.0 or 4.0 ng/ml was used. Remifentanil infusion was started 15 min before initiation of the remimazolam TCI protocol to ensure steady state remifentanil concentrations.

Target concentrations, number of target levels, and the number of volunteers were optimized before the trial using clinical trial simulations based on modeling results of Zhou *et al*.^5^ and Schüttler *et al*.^9^ (Supplemental Digital Content 2, https://links.lww.com/ALN/D787).

### Measurement of Drug Effect

Hypnotic drug effect was assessed by an anesthesiologist using the MOAAS score, which contains categories from 5 (fully alert) to 0 (no response to trapezius squeeze). All volunteers were asked to stay in bed with their eyes closed and not to engage in activities or spontaneous conversations except for reacting to the MOAAS assessment. MOAAS scores were collected at baseline, defined as 2 min before remifentanil (period 2 and 3) or remimazolam (period 1) drug infusion, and during the last 5 min of each step of the infusion protocol, in which it was assumed that the remimazolam concentration was at steady state.

After a response time of 2 min and observing a MOAAS score of 4 or less, TOTS was evaluated using a noxious stimulus (*i.e.*, tetanic stimulus, 100 Hz, 50 mA for maximally 30 s), which was delivered by means of the EZstim III (Halyard Health, USA). The stimulus was applied to the ulnar side of the not dominant arm of the subject. After a response time of 2 min and observing a MOAAS of 1 or less, TOL was evaluated and aimed at full visualization of the vocal cords using a size 3 curved Macintosh blade (HEINE Optotechnik GmbH & Co., Germany). Verbal acknowledgment, eye opening, grimacing, couching withdrawal, or any other purposeful or nonpurposeful movement, including jaw clenching and bucking after a stimulus, was defined as response. A subject was considered “tolerant” if no response occurred.

Throughout the drug administration periods, BIS observations were collected (BIS Vista, Medtronic, USA) using electrodes placed on the forehead. The median BIS value from –60 s up to the start of the MOAAS assessment was used for this analysis. An overview of the entire sequence of interventions and MOAAS classification is provided in Supplemental Digital Content 1 (https://links.lww.com/ALN/D786).

The volunteers breathed spontaneously through a tight-fitting mask to monitor the frequency, tidal volumes, and the end-tidal carbon dioxide. If required, to maintain normocapnia, manual breathing support and chin lift were allowed.

### Recovery Period

A recovery period started after completion of all infusion steps or if any of the safety criteria (*i.e.*, heart rate less than 40 beats per minute despite use of atropine, changes in cardiac rhythm resulting in clinically significant hemodynamic instability) were reached. During the recovery period, MOAAS scores were assessed with a 2-min interval for the first 30 min until a volunteer reached two sequential MOAAS scores of 5. A volunteer was discharged from the research unit when a modified Aldrete score of 10 and the discharge criteria of the hospital’s postanesthesia care unit were reached.

### Storage and Analysis of Blood Samples

Remimazolam, CNS7054, and remifentanil concentrations were determined in arterial plasma samples. For remimazolam and CNS7054, both analytes were determined in the same sample, for which a 2-ml sample was collected and, for remifentanil, a 3-ml sample was collected. All samples were collected at baseline (before start of infusion either remimazolam, period 1, or remifentanil, periods 2 and 3) and at each step in the infusion protocol at steady state (*i.e.*, after a minimum equilibration period of at least 25 min after target adjustment). Blood samples were collected in ethylenediaminetetraacetic acid tubes and stored on ice. Within 10 min (5 min for remifentanil samples) after collection, samples were centrifuged (Labofuge 400R, Heraeus Holding, GmbH, Germany). The remimazolam/CNS7054 samples were centrifuged for 10 min at 2,000*g* at 4°C, remifentanil samples were centrifuged at –20°C for 10 min at 2,000*g*. Plasma was transferred to cryovials (for remifentanil formic acid was added to ensure stability). Remimazolam samples were stored at –20°C, and remifentanil samples were stored at –80°C until analysis.

Remimazolam and CNS7054 were extracted from plasma samples after addition of methanol (200 µl) using protein precipitation and were analyzed using ultra-high-performance liquid chromatography–mass spectrometry using d4-remimazolam and d_4_-CNS7054 as internal standards. A Waters ACQUITY I–class chromatography system with a BetaSil Phenyl/Hexyl 5-µm column was used with a gradient of solvents A (water containing 0.1% formic acid) and B (acetonitrile; %A:%B: 0 min, 70:30; 1.2 min, 30:70; 1.3 min, 10:90; 1.5 min, 70:30). The lower and upper limits of quantification were 2 ng/ml and 2,000 ng/ml (remimazolam) and 20 ng/ml and 20,000 ng/ml (CNS7054), with a within-run precision of 6.6% or less and between-run precision of 3.7% or less.

Remifentanil was extracted from acidified (0.15% formic acid) plasma samples using protein precipitation, and extracts were analyzed on an API-5000 triple quadrupole mass spectrometer. Remifentanil-^13^C_6_ served as internal standard. A Waters ACQUITY I–class chromatography system with a ACQUITY UPLC HSS T3 1.8-µm column was used with a gradient of solvents A (water containing 0.1% formic acid) and B (acetonitrile; %A:%B: 0 min, 80:20; 1.9 min, 20:80; 2.2 min, 80:20). The lower and upper limits of quantification were 0.01 and 10 ng/ml, respectively. The within-run precision was 9.8% or less, and the between-run precision was 7.0% or less.

### Pharmacokinetics

Remimazolam and CNS7054 concentrations were analyzed using a population pharmacokinetic modeling approach. As a starting point for the population pharmacokinetic model, the remimazolam population pharmacokinetic model from Vellinga *et al.* was refitted on the pharmacokinetic data obtained in all three periods, in which it was assumed that 80% of remimazolam was converted to CNS7054 (if incorrect, this will affect values of the model parameters of the metabolite).^7^ Subsequently, we evaluated whether there was a pharmacokinetic interaction of remifentanil on the clearance of remimazolam or apparent clearance of CNS7054 according to equation 1.


$$\begin{array}{l} \begin{array}{lll} & CL_{cor}=CL_{i}\; & \times \;\left(1-{C_{remifentanil}}^{\gamma }/\left({C_{remifentanil}}^{\gamma }+C{50_{remifentanil}}^{\gamma }\right)\right)\;\; \end{array} \end{array}$$ (1)

For this, *post hoc* predicted remifentanil concentrations (C_remifentanil_) were used as input, which were based on the model by Eleveld et al.,^8^ the measured remifentanil concentrations, the characteristics (weight, age, and sex), and remifentanil dosing history of the volunteers in this study. In equation 1, CL_cor_ is the individual clearance of remimazolam (or apparent individual clearance of CNS7054) corrected for the absence or presence of remifentanil, CL_i_ is the individual clearance of remimazolam (or apparent individual clearance of CNS7054) in the absence of remifentanil, C_remifentanil_ is the predicted remifentanil concentration, C50_remifentanil_ is the predicted remifentanil concentration where clearance is 50% of the maximum clearance, and γ is the Hill parameter, which influences the shape of the relation.

### Pharmacodynamic Model Development

Model parameters were estimated using the first-order conditional estimation with interaction method for BIS and the LaPlacian algorithm for MOAAS, TOL, and TOTS, as implemented in NONMEM version 7.5 (Icon Development Solutions, USA). Pre- and postprocessing of data were performed in R version 4.0.3 (R core team, Austria, URL: https://www.R-project.org/, accessed October 10, 2020).

A sequential approach was used to develop all population pharmacodynamic models. For MOAAS and BIS, population pharmacodynamic models were previously developed based on data from the first period.^7^ These models (a proportional odds logistic regression model for MOAAS and a logistic regression model for BIS) were refitted to the pharmacodynamic data of all periods combined. Subsequently, a potential pharmacodynamic interaction between remimazolam and remifentanil was evaluated using equation 2.


$$\begin{array}{l} \begin{array}{lll} & Drug\;effect=Drug\;effect_{remimazolam}\; & \times \left(1+C_{remifentanil}/C50_{remifentanil}\right)\; \end{array} \end{array}$$ (2)

In this equation, drug effect is the overall drug effect, drug effect_remimazolam_ is the drug effect in the absence of remifentanil, C_remifentanil_ is the *post hoc* predicted remifentanil concentration, and C50_remifentanil_ is the *post hoc* predicted remifentanil concentration where the drug effect achieved by remimazolam alone is doubled.

The *a priori* pharmacodynamic models for MOAAS and BIS by Vellinga *et al.* include a tolerance mechanism, driven by competitive antagonism by CNS7054, attenuating the remimazolam drug effect.^7^ The need for inclusion of the tolerance mechanism was reevaluated during model development. Also, reevaluation of the random effects in the model, representing the between-subject variability on model parameters, was performed.

For the endpoints TOTS and TOL, no *a priori* population pharmacodynamic models were available. Therefore, model development initiated by fitting a logistic regression model parameterized as equation 3.


$$\begin{array}{l} P\left(Y=0\right)=1-P\left(Y=1\right)=1*\left(1-Drug\;effect\right) \end{array}$$ (3)

In these equations, P(Y = 0) and is the probability of observing response (Y = 0) and equal to 1 - probability of no response, P(Y = 1), to laryngoscopy or tetanic stimulation, in which it is assumed that there is 100% probability of response in the absence of drug. This assumption was evaluated during model development. Drug effect is the effect of remimazolam on the probability of observing response to laryngoscopy or tetanic stimulation. The drug effect was parameterized according to equation 4 and included competitive inhibition by CNS7054.


$$\begin{array}{l} \begin{array}{lll} & Drug\;effect\;\;\; & =\frac{C_{remimazolam}/C50_{remimazolam}}{\begin{array}{lll} & (1+C_{remimazolam\;}/\;C50_{remimazolam}\;\; & +C_{CNS7054}/C50_{CNS7054})\; \end{array}}\;\; \end{array} \end{array}$$ (4)

In this equation, C_remimazolam_ and C_CNS7054_ are the *post hoc* predicted remimazolam and CNS7054 plasma concentrations and C50_remimazolam_ and C50_CNS7054_ are the predicted plasma concentrations of remimazolam and CNS7054 where 50% of the maximum drug effect is achieved. The need for inclusion of the competitive inhibition by the metabolite in this equation was evaluated. Subsequently, the potential for interaction between remifentanil and remimazolam was evaluated using equation 5.


$$\begin{array}{l} \begin{array}{lll} & C50_{remimazolam,\;cor}=C50_{remimazolam}\;\; & \times \;\left(1-C_{remifentanil}/\left(C_{remifentanil}+C50_{remifentanil}\right)\right)\; \end{array} \end{array}$$ (5)

In this equation, C50_remimazolam_ and C50_remimazolam,cor_ represent the concentration of remimazolam where 50% of the maximum drug effect is achieved in the absence of remifentanil or corrected for the presence of remifentanil, respectively. C50_remifentanil_ is the *post hoc* predicted remifentanil concentration (C_remifentanil_), where the C50_Remimazolam,cor_ is 50% compared to C50_remimazolam_.

For BIS, an interaction model assuming an additive interaction between remifentanil and remimazolam and CNS7054 was also evaluated according to equation 6.


$$\begin{array}{l} \begin{array}{lll} & Drug\;effect\;\; & =\frac{C_{remimazolam}/C50_{remimazolam}+C_{remifentanil}\;/\;C50_{remifentanil}}{\begin{array}{lll} & (1+C_{remimazolam\;}/C50_{remimazolam}+C_{CNS7054}\; & /C50_{CNS7054}+C_{remifentanil}/C50_{remifentanil})\; \end{array}}\;\; \end{array} \end{array}$$ (6)

Random effects on all parameters were explored by assuming either a log-normal distribution or additive normal distribution in the logit domain. Models were compared by the change in objective function value for nonnested models and a likelihood ratio test for nested models with a decrease in objective function value of 6.64 corresponding to a significant (*P* < 0.01) improvement in model fit when one additional degree of freedom is introduced. Further, model performance was assessed by visual predictive checks, plausibility and uncertainty of the model parameters and a condition number less than 1,000. Uncertainty in the model parameters was estimated using log-likelihood profiling.

### Simulations

Simulations were conducted to illustrate the pharmacokinetics and pharmacodynamics of combinations of remimazolam and remifentanil. TCI of remimazolam was simulated targeting effect-site concentrations ranging from 150 to 2,000 ng · ml^-1^. TCI simulations were based on the final population pharmacokinetic model from this study and assumed a rate constant for effect-site equilibration of 0.135 min^-1^ and a maximum infusion rate of 6 mg · min^-1^. Before initiation of remimazolam, remifentanil TCI was initiated using the model by Eleveld *et al*.^8^ targeting concentrations of 0, 0.1, 0.5, 1, 2, and 4 ng/ml. In the simulations, a typical individual of 70 kg bodyweight was assumed. Descriptive statistics were used to summarize the predicted BIS, MOAAS, TOTS, and TOL at 10, 60, and 120 min after the initiation of remimazolam TCI administration. All simulations were conducted using the RxODE package (version 1.0.9) in R.

## Results

This clinical trial included 24 volunteers, of whom 13 (54.2%) were male. The volunteers had a mean (minimum, maximum) age of 43 (19, 70) years, weight of 74 (51, 106) kg, height of 175 (159, 192) cm, and body mass index of 24.1 (20.2, 29.4) kg · m^-2^. Cardiovascular and respiratory homeostasis was maintained throughout the study duration (Supplemental Digital Content 3, https://links.lww.com/ALN/D788).

### Interim Analysis

After the inclusion of six healthy volunteers, it appeared that the remifentanil target concentrations of 2 ng/ml and 4 ng/ml in period 3 did not result in sufficient granularity for describing the exposure-response around MOAAS 2 or 3. Based on data of these six healthy volunteers, target concentrations of both remimazolam and remifentanil in period 3 were optimized in line with the initial trial design optimization described in Supplemental Digital Content 2 (https://links.lww.com/ALN/D787). For the remainder of the trial, in period 3, volunteers were randomized for remifentanil TCI target plasma concentrations of 0.1 or 1.0 ng/ml instead of 2.0 or 4.0 ng/ml. The remimazolam TCI effect site target concentrations for the respective remifentanil targets were also optimized and were 125, 225, 275, 525, 925, and 1,700 ng/ml during step-up and 925, 525, 275, 225, and 125 ng/ml during step-down and 50, 100, 125, 225, 400, and 1,300 during step-up and 400, 225, 125, 100, and 50 during step-down. The final numbers of subjects across the remifentanil target concentrations were 8, 24, 10, 4, and 2 for the 0.1, 0.5, 1, 2, and 4 ng/ml targets, respectively.

### Performance of Target Controlled Infusion

The measured remimazolam and remifentanil concentrations by TCI target in sessions 2 and 3 are shown in Supplemental Digital Content 4 (supplemental fig. 1, https://links.lww.com/ALN/D789). The median prediction errors for remimazolam was +5.6% (95% CI, –2.6% to +14%) and +16% (95% CI, +7.1% to +25%) for sessions 2 and 3, respectively. The corresponding median absolute prediction errors were 20% (95% CI, 15% to 25%) and 26% (95% CI, 20% to 33%). For remifentanil, median prediction errors were –8.1% (95% CI, –16% to +0.0%) and –11% (95% CI, –19% to –1.9%) and median absolute prediction errors were 21% (95% CI, 17% to 25%) and 21% (95% CI, 15% to 27%) for sessions 2 and 3, respectively.

### Pharmacokinetics

The influence of remifentanil coadministration on the pharmacokinetics of remimazolam and CNS7054 was tested on remimazolam clearance and apparent CNS7054 clearance. No influence was detected on the clearance of remimazolam (decrease in objective function value, 0.0 points; *P* = 1.00), but a clear influence of remifentanil was detected on the apparent clearance of CNS7054 (decrease in objective function value, –315.4 points; *P* < 0.001). The remifentanil concentration reducing apparent CNS7054 clearance by 50% was estimated as 8.0 ng/ml (95% CI, 5.5 to 13.4 ng/ml). A detailed description of the model development and population pharmacokinetic model, including numerical and graphical model diagnostics, is provided in Supplemental Digital Content 5 (https://links.lww.com/ALN/D790).

### Pharmacodynamic Model Development

**Fig. 1.**
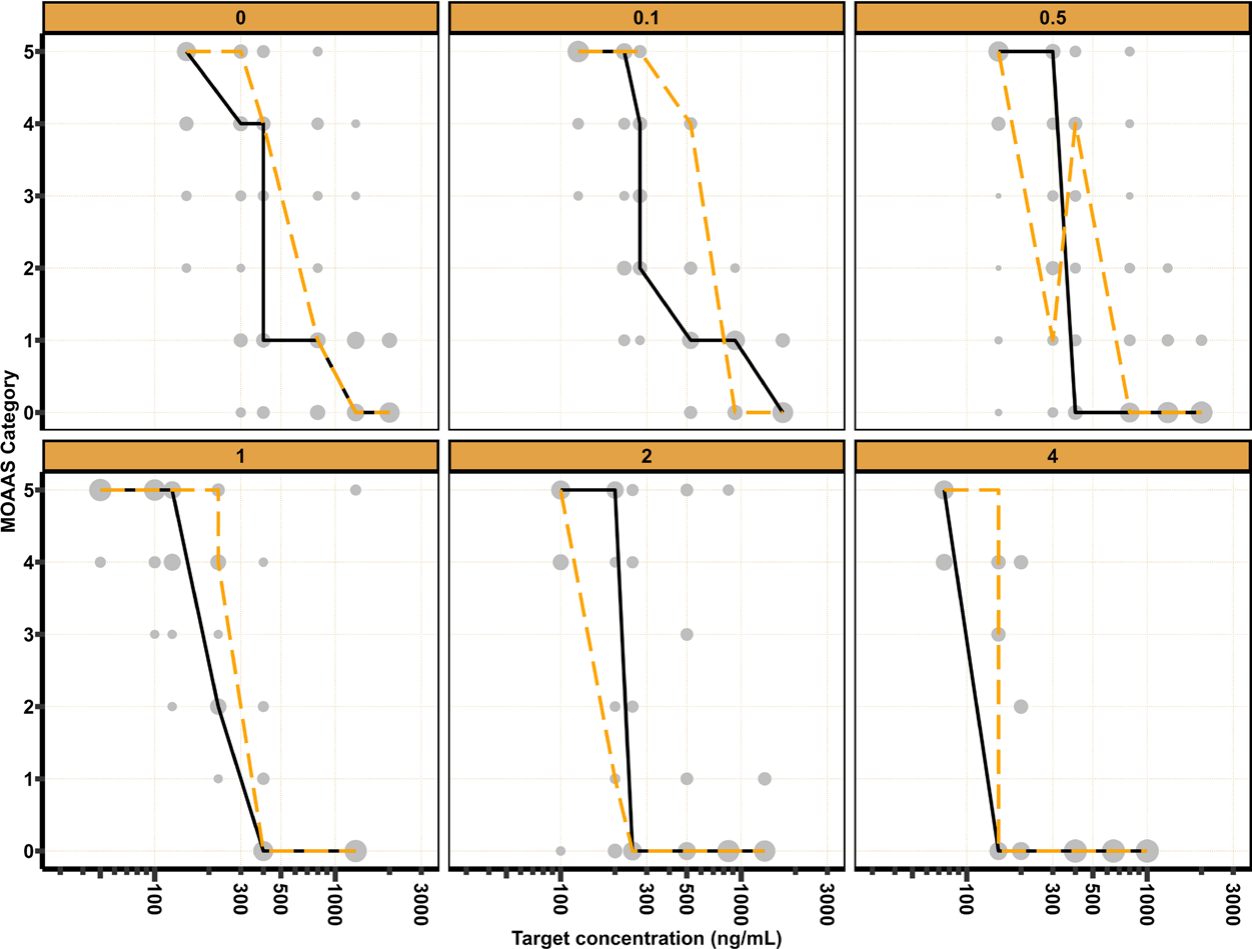
Modified Observer’s Assessment of Alertness and Sedation (MOAAS) scores *versus* remimazolam target concentrations stratified by remifentanil target concentrations. *Gray dots* represent observed MOAAS scores (*size* indicates relative frequency per remimazolam target concentration). *Black solid line* and *orange dashed line* represent the most frequently observed and predicted MOAAS scores, respectively.

**Fig. 2.**
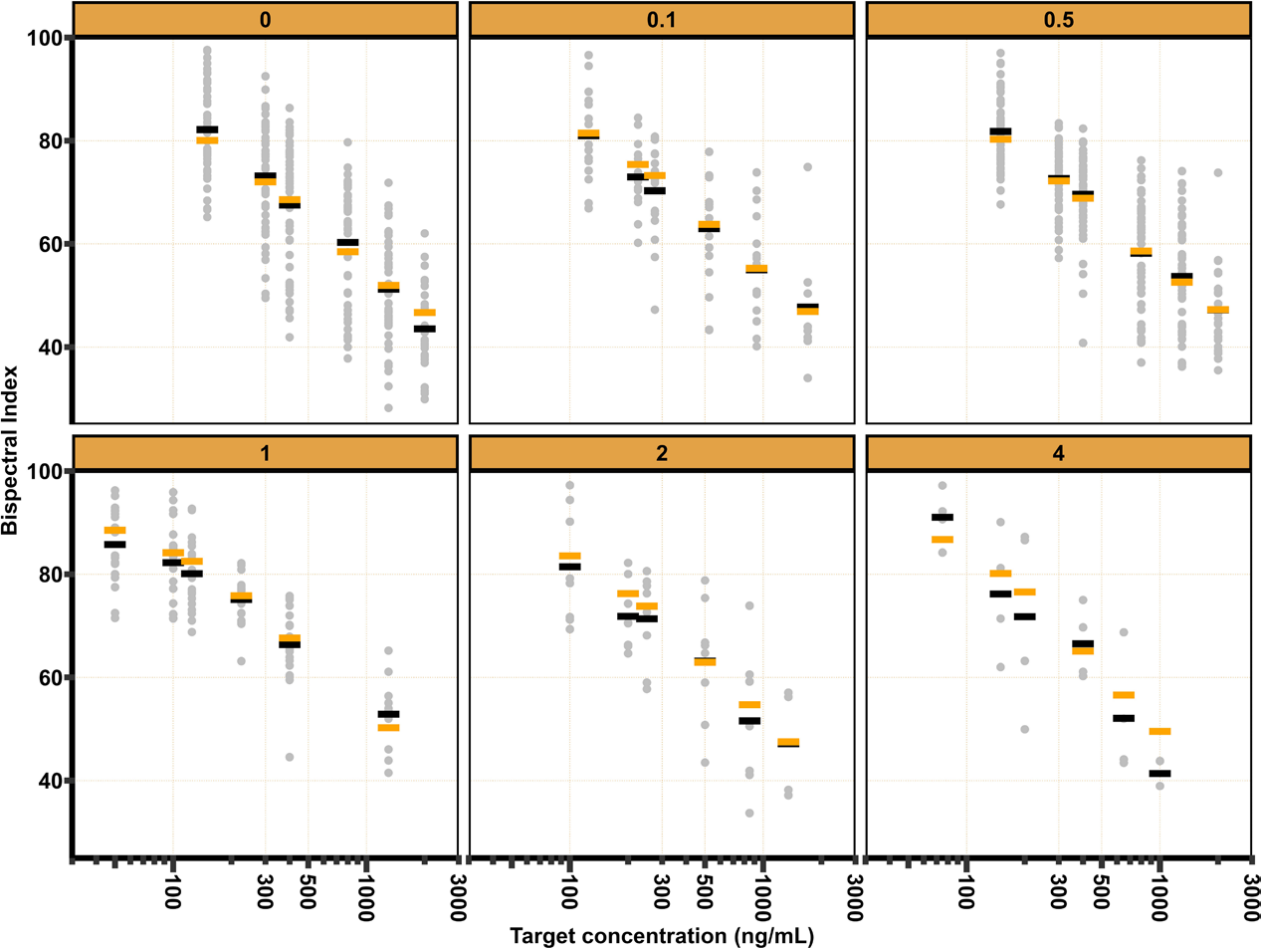
Bispectral Index values *versus* remimazolam target concentrations stratified by remifentanil target concentration. *Gray dots* represent observed Bispectral Index values. *Black* and *orange lines* represent the observed and predicted mean Bispectral Index values, respectively.

**Fig. 3.**
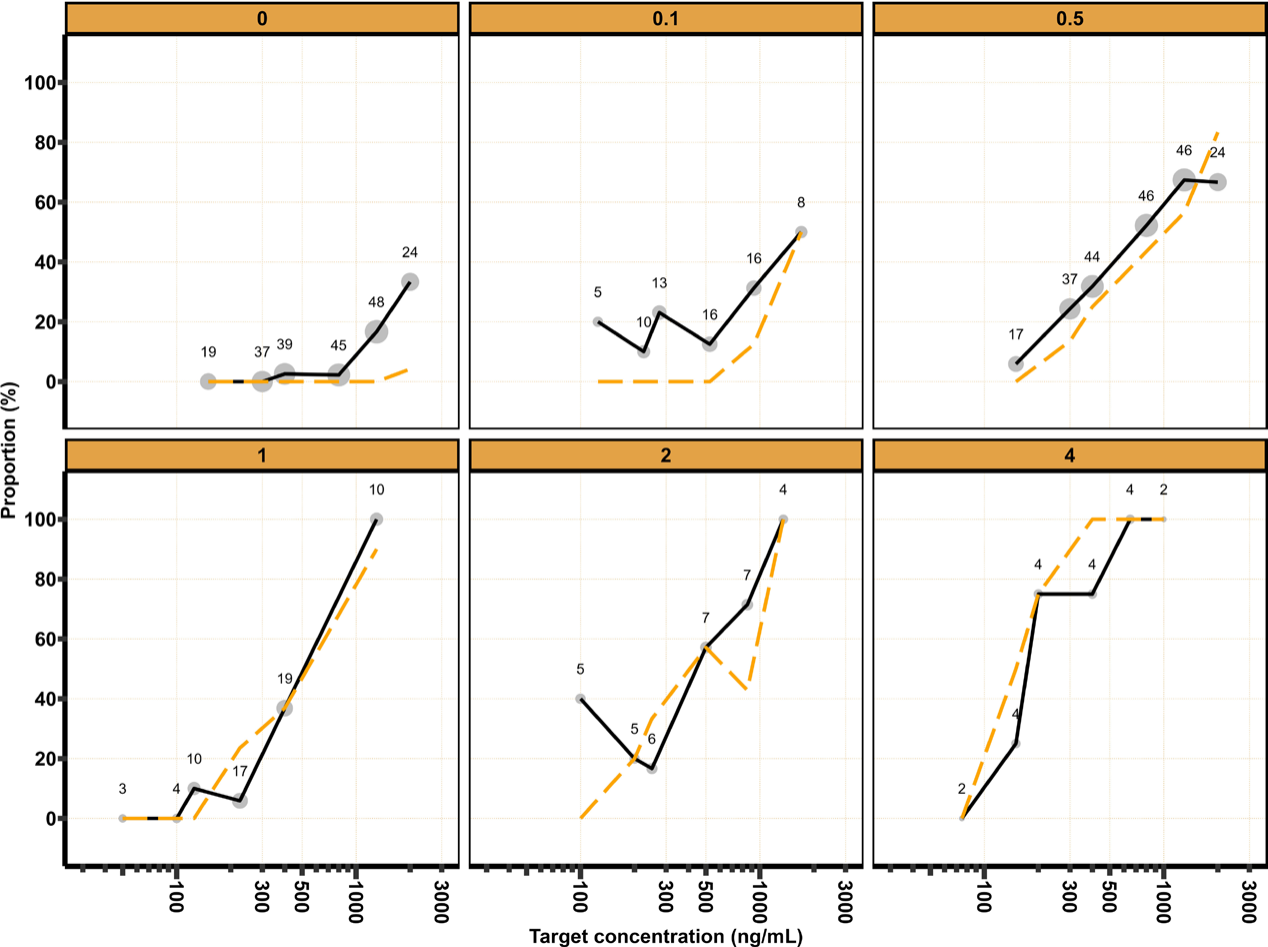
Tolerance to tetanic stimulation *versus* remimazolam target concentrations stratified by remifentanil target concentration. *Black solid line* and *orange dashed line* represent the most frequently observed and predicted proportions of observations that demonstrate tolerance to tetanic stimulation, respectively. *Size* of *gray dots* and *numbers* represent the number of observations.

**Fig. 4.**
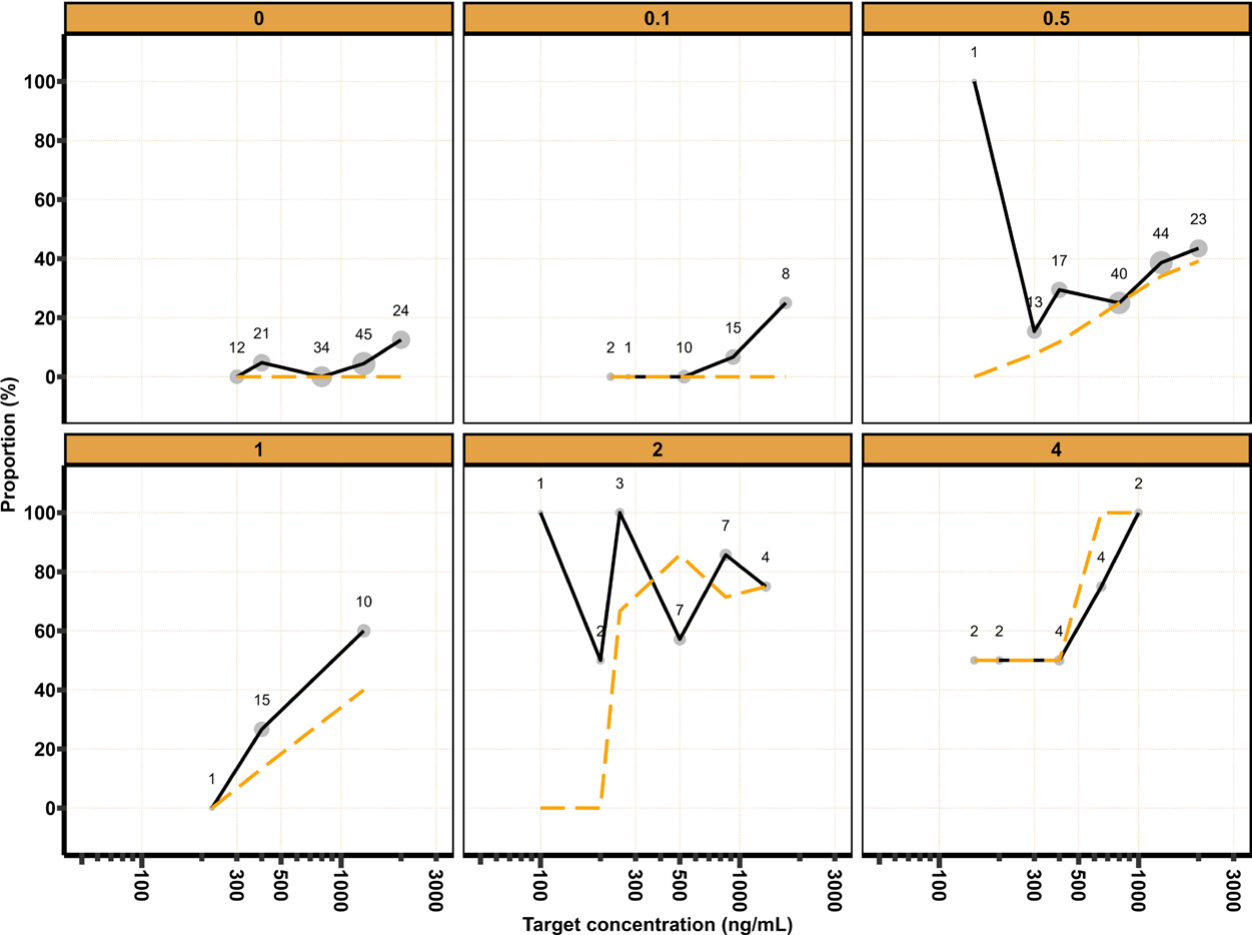
Tolerance to laryngoscopy *versus* remimazolam target concentrations stratified by remifentanil target concentration. *Black solid line* and *orange dashed line* represent the most frequently observed and predicted proportions of observations that demonstrate tolerance to laryngoscopy, respectively. *Size* of *gray dots* and *numbers* represent the number of observations.

In total, 1,025 MOAAS, 891 BIS, 611 TOTS, and 374 TOL observations were collected in three periods, which are displayed in figures 1 to 4. For MOAAS and BIS, model development was initiated by refitting the models developed by Vellinga *et al.*^7^ Addition of a remifentanil interaction on drug effect according to equation 2 improved the model fit for MOAAS (decrease in objective function value, –171.6 points; degrees of freedom, 1; *P* < 0.001). For BIS, a synergistic interaction according to equation 2 improved the model (decrease in objective function value, –8.9 points; degrees of freedom, 1; *P* = 0.002), but an additive interaction model on drug effect, according to equation 6, improved the model fit to a greater extent (decrease in objective function value, –26.3 points; degrees of freedom, 1; *P* < 0.001).

For TOTS and TOL, model development began by fitting a logistic regression model with a drug effect for remimazolam. Addition of a remifentanil interaction on C50_remimazolam_ according to equations 4 and 5 improved the model fit for TOTS (decrease in objective function value, –134.2 points; degrees of freedom, 1; *P* < 0.001) and TOL (decrease in objective function value, –83.1 points; degrees of freedom, 1; *P* < 0.001).

Removal of the competitive antagonism by CNS7054 worsened the model fit for MOAAS (decrease in objective function value, 328.4 points; degrees of freedom, 1; *P* < 0.001) and BIS (decrease in objective function value, 325.3 points; degrees of freedom, 1; *P* < 0.001). Therefore, competitive antagonism by CNS7054 was preserved in both of these models. Competitive antagonism by CNS7054 was not supported by the TOTS data (decrease in objective function value, –1.5 points; degrees of freedom, 1; *P* = 0.22) and TOL data (decrease in objective function value, –1.4 points; degrees of freedom, 1; *P* = 0.24).

Estimated parameters and an overlay of the mean observed and predicted MOAAS score, BIS, TOTS, and TOL stratified by target remifentanil concentration are provided in table 1 and figures 1 to 4, respectively. Model development details, model code, visual predictive checks, and log-likelihood profiles of the population pharmacodynamic models are shown in Supplemental Digital Content 6 (https://links.lww.com/ALN/D791).

**Table 1.** Parameter Estimates of Pharmacodynamic Models for Bispectral Index, Tolerance to Laryngoscopy, Tolerance to Tetanic Stimulation, and Modified Observer’s Assessment of Alertness and Sedation Scores

Parameter Name	Parameter	Estimates	Lower Limit (95% CI)	Upper Limit (95% CI)	Interindividual Variability	Lower Limit (95% CI)	Upper Limit (95% CI)	Shrinkage (%)
Bispectral Index								
Baseline	BASEB	94.0	92.9	95.1	0.07^*^	0.02	0.19	19.8
Maximum drug effect in logit domain	EMAXB	3.5	3.3	3.8	10.3%	5.5%	18.0%	29.2
C50, remimazolam (ng/ml)	EC50B	202	169	243				
C50, CNS7054 (ng/ml)	EC50BM	9,495	7,971	11,387				
C50, remifentanil (ng/ml)	EC50BR	7.96	4.99	17.51				
Additive residual error (logit domain SD)		0.31	0.29	0.64				
Proportional residual error (logit domain %CV)		18.7	15.5	21.9				
Tolerance to laryngoscopy								
C50, remimazolam (ng/ml)	EC50T	54,128	19,930	179,871	231.3%	101.7%	1065.1%	31.2%
C50, remifentanil (ng/ml)	EC50TR	0.02	0.01	0.06				
Tolerance to tetanic stimulation								
C50, remimazolam (ng/ml)	EC50TO	12,133	6,568	24,343	118.8%	67.7%	255.7%	24.2%
C50, remifentanil (ng/ml)	EC50TOR	0.04	0.02	0.08				
MOAAS								
Baseline in logit domain MOAAS ≤ 0	BASE0	–9.7	–8.7	–10.8	0.99	0.52	1.98	7.5%
Baseline in logit domain MOAAS ≤ 1	BASE1	2.0	1.7	2.3				
Baseline in logit domain MOAAS ≤ 2	BASE2	1.1	0.8	1.4				
Baseline in logit domain MOAAS ≤ 3	BASE3	0.8	0.6	1.1				
Baseline in logit domain MOAAS ≤ 4	BASE4	2.2	1.9	2.6				
Maximum drug effect in logit domain	EMAXM	11.7	10.3	13.4				
C50, remimazolam (ng/ml)	EC50M	211	190	242				
C50, CNS7054 (ng/ml)	EC50MM	3,160	2,040	4,739				
C50, remifentanil (ng/ml)	EC50R	2.5	2.0	3.2				
Hill parameter	GAMMAM	1.75	1.5	2.1				

The interindividual variability was expressed as SD in the logit domain. The residual variability of the BIS model was expressed as SD in the logit domain. The lower limit and upper limit of the 95% CI of the parameters were derived using log-likelihood profiling.

* Abbreviations in the Parameter column correspond to the defined model parameters in the supplemental digital content.

%CV, coefficient of variation in percentage; MOAAS, Modified Observer’s Assessment of Alertness and Sedation.

### Simulations

**Fig. 5.**
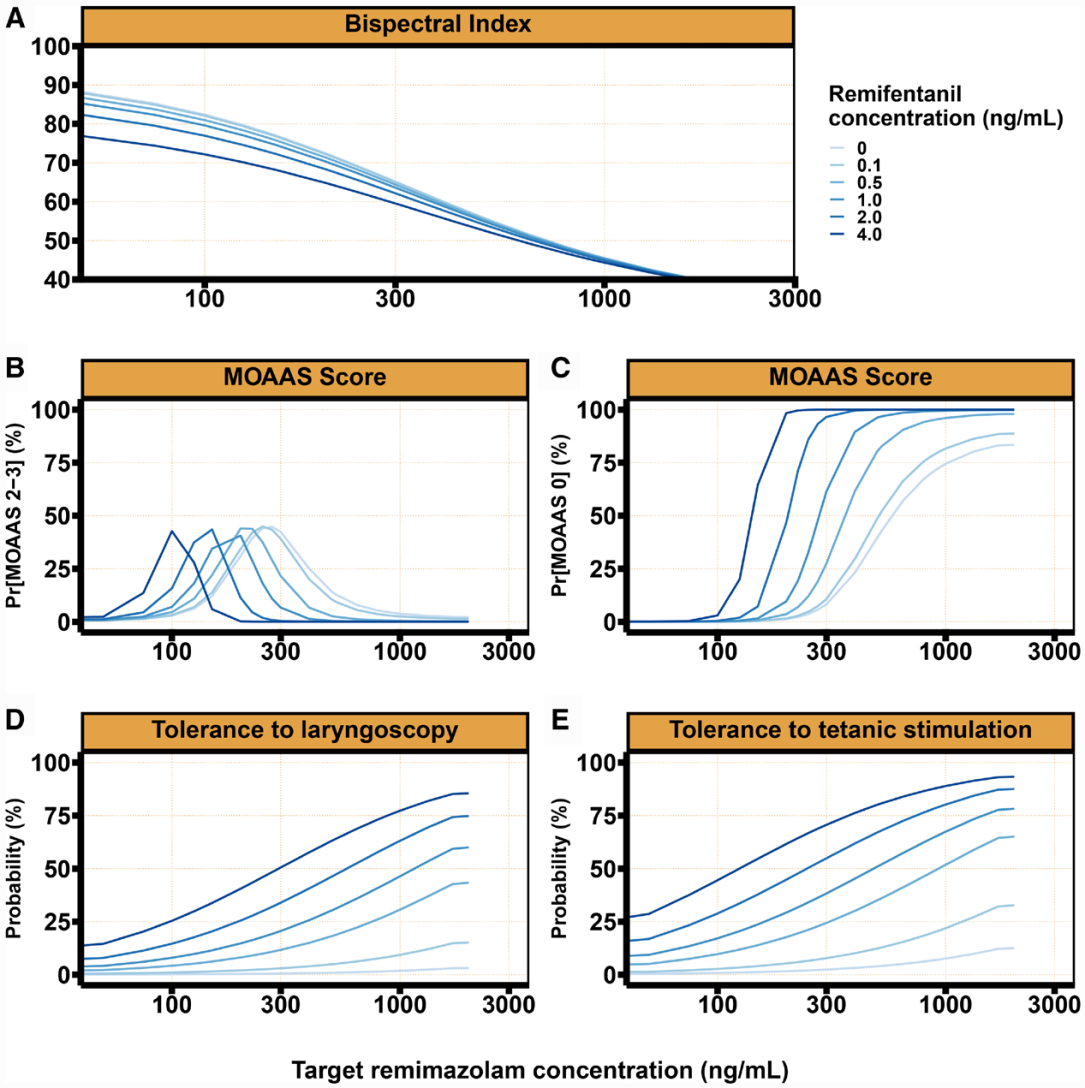
Predicted relationship between remimazolam target concentration and Bispectral Index, Modified Observer’s Assessment of Alertness and Sedation (MOAAS) scores, tolerance to tetanic stimulation, and tolerance to laryngoscopy stratified by remifentanil target concentration after 10 min of target controlled infusion of remimazolam.

Simulation results after 10, 60, and 120 min of remimazolam and remifentanil TCI are summarized in table 2, figure 5, and Supplemental Digital Content 7 (https://links.lww.com/ALN/D792 for the 60- and 120-min scenarios).

**Table 2.** Predicted Relationship between Remimazolam Target Concentration and Bispectral Index, Modified Observer’s Assessment of Alertness and Sedation Scores, Tolerance to Tetanic Stimulation, and Tolerance to Laryngoscopy Stratified by Remifentanil Concentration after 10 Minutes of Target Controlled Infusion of Remimazolam

Remifentanil Target Concentration (ng/ml)	Remimazolam Target Concentration (ng/ml)	BIS	TOL (%)	TOTS (%)	MOAAS 5 (%)	MOAAS 4 (%)	MOAAS 3 (%)	MOAAS 2 (%)	MOAAS 1 (%)	MOAAS 0 (%)
0	150	77	0.3	1.2	38.4	47.1	7.7	4.4	2.1	0.3
	300	65	0.6	2.4	2.3	15.7	15.7	26.6	31.5	8.3
	400	60	0.7	3.2	0.7	5.5	7.1	18.0	45.7	23.1
	800	48	1.5	6.2	0.1	0.9	1.2	4.1	26.6	67.2
	1,300	43	2.3	9.7	0.1	0.4	0.6	2.2	16.8	79.9
	2,000	40	3.1	12.5	0.0	0.3	0.5	1.7	13.9	83.4
0.1	150	77	1.5	4.0	34.8	48.7	8.7	5.1	2.4	0.4
	300	65	3.0	7.8	1.7	12.4	13.5	25.6	36.1	10.7
	400	60	4.0	10.1	0.5	4.0	5.3	14.7	45.9	29.6
	800	48	7.6	18.4	0.1	0.6	0.8	2.8	20.2	75.5
	1,300	43	11.8	26.8	0.0	0.3	0.4	1.4	11.8	86.0
	2,000	40	15.2	32.8	0.0	0.2	0.3	1.1	9.6	88.7
0.5	150	76	6.2	13.9	22.1	50.7	13.3	8.7	4.4	0.7
	300	64	11.7	24.4	0.5	4.4	5.8	15.7	46.0	27.5
	400	59	15.0	30.1	0.1	1.1	1.6	5.1	30.5	61.6
	800	48	26.1	46.3	0.0	0.1	0.2	0.6	5.1	94.1
	1,300	42	36.5	58.3	0.0	0.1	0.1	0.3	2.4	97.2
	2,000	40	43.4	65.1	0.0	0.0	0.1	0.2	1.8	97.9
1	150	75	11.5	23.7	11.4	43.5	19.0	15.5	9.0	1.6
	300	64	20.6	38.3	0.1	1.1	1.6	5.1	30.7	61.4
	400	59	25.7	45.3	0.0	0.2	0.3	1.0	8.9	89.5
	800	48	40.9	62.3	0.0	0.0	0.0	0.1	0.7	99.2
	1,300	42	52.9	72.9	0.0	0.0	0.0	0,0	0.3	99.7
	2,000	40	60.0	78.2	0.0	0.0	0.0	0,0	0.2	99.8
2	150	72	20.4	37.8	2.6	17.6	16.8	26.7	29.1	7.2
	300	62	33.9	54.9	0.0	0.1	0.1	0.3	2.9	96.6
	400	58	40.6	61.9	0.0	0.0	0.0	0.0	0.4	99.6
	800	48	57.8	76.4	0.0	0.0	0.0	0.0	0.0	100.0
	1,300	42	69.0	84.1	0.0	0.0	0.0	0.0	0.0	100.0
	2,000	40	74.8	87.6	0.0	0.0	0.0	0.0	0.0	100.0
4	150	68	33.8	54.6	0.1	1.0	1.4	4.5	28.5	64.5
	300	60	50.5	70.7	0.0	0.0	0.0	0.0	0.0	100.0
	400	56	57.7	76.3	0.0	0.0	0.0	0.0	0.0	100.0
	800	47	73.1	86.5	0.0	0.0	0.0	0.0	0.0	100.0
	1,300	42	81.6	91.3	0.0	0.0	0.0	0.0	0.0	100.0
	2,000	39	85.5	93.3	0.0	0.0	0.0	0.0	0.0	100.0

BIS, Bispectral Index; MOAAS, Modified Observer’s Assessment of Alertness and Sedation; TOL, tolerance to laryngoscopy; TOTS, tolerance to tetanic stimulation.

A small influence of remifentanil on the remimazolam pharmacokinetic–pharmacodynamic relationship with BIS can be observed after 10 min of target controlled remimazolam administration. As seen in table 2, the predicted BIS decreased from 65 to 60 at a remimazolam target concentration of 300 ng/ml when 4.0 ng/ml remifentanil is targeted *versus* no administration of remifentanil. In contrast, simulations indicate a clear influence of remifentanil on the remimazolam pharmacokinetic–pharmacodynamic relationship with TOL, TOTS, and MOAAS. For example, for a 10-min TCI administration targeting a remimazolam concentration of 300 ng/ml, the probability for observing TOL, TOTS, and a MOAAS score of 0 increased from 0.5%, 2.4%, and 8.3% in the absence of remifentanil to 50.5%, 70.6%, and 99.9% at a remifentanil concentration of 4 ng/ml.

The remimazolam drug effect attenuated over time for both MOAAS and BIS. For example, at a target remimazolam concentration of 2,000 ng/ml in the absence of remifentanil, the probability for observing a MOAAS of 0 decreased from 83.4% to 74.4% and 64.3% and the BIS increased from 40 to 45 and 50 after, respectively, 10, 60, and 120 min of target controlled infusion. When a 2,000 ng/ml remimazolam target was combined with 4 ng/ml remifentanil, the predicted probability for observing a MOAAS of 0 remained 100%, whereas the predicted BIS increased from 39 to 45 and 51, respectively, 10, 60, and 120 min after the start of the target controlled infusion. The tolerance development over time induces a rightward shift in the exposure–response relationships for BIS and MOAAS, as seen in figure 5 and supplemental figures 1 and 2 in Supplemental Digital Content 7 (https://links.lww.com/ALN/D792). The remimazolam drug effect for both TOTS and TOL did not change over time.

After 10 min of TCI, the predicted probability of a MOAAS score of 2 or 3 was highest and was 45%, 45%, and 44% for a remimazolam target concentration of 275, 250, or 200 ng/ml, respectively, combined with 0, 0.1, or 0.5 ng/ml remifentanil. At these remimazolam–remifentanil combinations, the predicted BIS values were 67, 68, and 71, respectively.

After 10 min of TCI, the probability of observing a MOAAS score of 0 was higher than 99% when combining remimazolam target concentrations of at least 800 ng/ml remimazolam and 1.0 ng/ml remifentanil. After 120 min, predicted probabilities for MOAAS 0 higher than 99% required remimazolam target concentrations 800 ng/ml or greater and remifentanil concentrations of at least 2.0 ng/ml. The highest probabilities of observing TOTS and TOL were 93.3% and 85.5%, respectively, at a remimazolam target concentration of 2,000 ng/ml combined with a remifentanil target concentration of 4.0 ng/ml.

## Discussion

In this clinical trial, we were able to characterize the influence of remifentanil on the sedative properties of remimazolam as measured by MOAAS, BIS, TOL, and TOTS. On the one hand, apparent clearance of CNS7054 is inhibited by remifentanil; on the other hand, a pharmacodynamic interaction between remifentanil and remimazolam was identified on all studied pharmacodynamic measures (MOAAS score, BIS, TOL, and TOTS). Simulations demonstrated that the probability of observing TOL, TOTS, and a lower BIS or MOAAS score at a certain remimazolam target concentration increased with increasing remifentanil concentrations. Our model-based analysis also indicated development of acute tolerance in sessions 2 and 3 of the trial in the presence of remifentanil for BIS and MOAAS, but not for TOL and TOTS. Remimazolam target concentrations between 275 and 200 ng/ml combined with remifentanil target concentrations between 0 and 0.5 ng/ml, respectively, resulted in the highest probability of observing a MOAAS score of 2 or 3. TOTS and TOL were predicted to be 93.3% and 85.5% at a remimazolam target concentration of 2,000 ng/ml combined with a remifentanil target concentration of 4.0 ng/ml.

No pharmacokinetic drug–drug interactions were expected based on the drug label of remimazolam.^3,4^ While no pharmacokinetic interactions were identified between remimazolam and remifentanil, a pharmacokinetic interaction between remifentanil and remimazolam was quantified on the apparent clearance of CNS7054. A C50 value of 8.0 ng/ml remifentanil was estimated in the population pharmacokinetic analysis, which implies that the apparent CNS7054 clearance is reduced by approximately 40% at the highest remifentanil concentration of 4.0 ng/ml studied in our trial. A study in patients with impaired kidney function indicated that the apparent CNS7054 clearance is predominantly mediated by renal elimination.^10^ Remifentanil is expected to reduce cardiac output.^11–13^ At the same time, remifentanil acid, the principal remifentanil metabolite, is excreted *via* the kidneys. Therefore, we hypothesize that either the reduced renal elimination of CNS7054 originates from a reduction in renal blood flow secondary to a remifentanil-induced reduction in cardiac output, or remifentanil acid competitively inhibits CNS7054 renal excretion.

A synergistic drug–drug interaction between remimazolam and remifentanil has been documented in the literature for BIS by Zhou *et al*.^5^ However, we found no support for a synergistic drug–drug interaction on BIS, but instead identified an additive drug–drug interaction. A direct comparison between both models is inappropriate due to different parameterizations of the models, with Zhou *et al*. using remifentanil infusion rate (mg/kg) as a proxy for remifentanil plasma concentrations.^5^ In addition, the imbalance in the absence or presence of remifentanil between patient populations (general anesthesia *vs.* procedural sedation *vs.* healthy volunteer studies) in the analysis by Zhou *et al*. potentially confounds the estimated interaction and any resulting comparison against our results.

We have previously described an acute tolerance phenomenon for MOAAS and BIS, which we hypothesize originates from competitive antagonism by CNS7054.^7^ The current analysis confirmed acute tolerance development and showed that acute tolerance development was also apparent in periods 2 and 3 of the trial in the presence of remifentanil. Additionally, the pharmacokinetic interaction of remifentanil on apparent CNS7054 clearance indicates that tolerance is more pronounced when remifentanil is coadministered as CNS7054 concentrations increase as a result of the decreased apparent CNS7054 clearance. This could worsen acute tolerance development. However, simulations of 60 and 120 min of TCI administration of remimazolam showed that the acute tolerance phenomenon has limited clinical impact on BIS and MOAAS in the presence or absence of remifentanil.

No acute tolerance was observed for TOL and TOTS, which could call into question acute tolerance development for remimazolam as this appears in contrast to the findings for BIS and the MOAAS score. TOL and TOTS observations were, however, mainly collected at the higher remimazolam target concentrations for ethical reasons, and therefore the time between collection of the first and last observations for TOL and TOTS was shorter compared to the time between first and last observations for MOAAS and BIS. This could have attenuated the apparent influence of the acute tolerance development for TOL and TOTS in our clinical trial. An alternative explanation is that remimazolam has limited analgesic properties, and therefore, acute tolerance development will only be expressed for clinical endpoints related to the hypnotic properties of remimazolam (*i.e.*, MOAAS and BIS).

The interim analysis showed that the initial trial design was not optimal to characterize the exposure–response relationship for MOAAS 2 or 3. The final analysis of this study showed that remimazolam target concentrations between 275 and 200 ng/ml combined with 0 to 0.5 ng/ml remifentanil target concentrations provided the highest probability of observing a MOAAS score of 2 or 3. Therefore, lowering the remifentanil target concentrations to 0.1 or 1.0 ng/ml after the interim analysis translated into a higher probability of observing a MOAAS score of 2 or 3, although it should be noted that this was predicted to be about 45%. This indicates that titrating patients to a MOAAS score of 2 or 3 is not easy with the selected target concentrations. At the same time, the interim analysis reduced the planned number of subjects for the higher remifentanil target concentrations (2.0 and 4.0 ng/ml) and negatively affected our ability to adequately characterize the higher ends of the exposure–response relationships for TOL and TOTS. The highest probabilities of TOTS and TOL were predicted to be 93.3% and 85.5%, and therefore, the studied maximum remimazolam target concentration, 2,000 ng/ml, and remifentanil target concentration, 4.0 ng/ml, were most likely too low. We are therefore not very confident in recommending appropriate target concentrations of remimazolam and remifentanil for TOL and TOTS and consider this a limitation of the clinical trial.

In conclusion, both a pharmacokinetic and a pharmacodynamic drug–drug interaction between remimazolam and remifentanil could be quantified in this clinical trial. Consequently, the probability of observing TOL, TOTS, and a lower MOAAS score and BIS at a certain remimazolam target concentration increased with increasing remifentanil concentrations. Acute tolerance also developed in the presence of remifentanil, which attenuated the hypnotic drug effects of remimazolam. Remimazolam target concentrations between 275 and 200 ng/ml combined with 0 to 0.5 ng/ml remifentanil target concentrations have the highest probability of observing a MOAAS score of 2 or 3.

### Acknowledgments

The authors would like to thank Ernesto Muskiet, M.D., Jop van den Berg, M.D., Ph.D., Rutger Spruit, M.D., and Sascha Meier, M.D., Ph.D. (all from Department of Anesthesiology, University Medical Center Groningen, Groningen, The Netherlands), for their incredible help as study anesthetists. Further, the authors would like to thank Charlotte Beukers, R.N., Froukje Knotnerus, R.N. (both from Department of Anesthesiology, University Medical Center Groningen), Peter Stuijt, M.Sc. (Department of Clinical Pharmacy and Pharmacology, University Medical Center Groningen), and Harry Scheeringa, R.N. (Department of Anesthesiology, University Medical Center Groningen) for their help during screening, preparation, and sample collection during the study. We thank the staff of the holding area of the University Medical Center Groningen for their patience during the study periods and deeply extend appreciation to all the volunteers who participated in this trial. The authors thank Tom de Smet, Ph.D. (Demed Medical, Sinaai, Belgium) for his help with RUGLOOP and Rob Spanjersberg, M.Sc. (Department of Anesthesiology, University Medical Center Groningen), and Danilo Uvalin, M.Sc. Pharmacy (Paion Deutschland GmbH, Aachen, Germany), for their help with the ethical committee and study management. Finally, the authors thank Omair Sahgal, M.B.Ch.B., M.Res., and Adam Morley, M.D., Ph.D. (both from Paion UK Ltd., London, United Kingdom), for their medical sponsor oversight.

### Research Support

The clinical trial was supported by Paion UK Ltd. (London, United Kingdom; Clinical Trial Agreement VIG-NFU-STZ-Acron 2019).

### Competing Interests

Dr. Struys declares that his research group or department received (over the last 3 yr) research grants and consultancy fees from The Medicines Company (Parsippany, New Jersey), Masimo (Irvine, California), Becton Dickinson (Eysins, Switzerland), Fresenius (Bad Homburg, Germany), Dräger (Lübeck, Germany), Paion (Aachen, Germany), and Medtronic (Dublin, Ireland). He receives royalties on intellectual property from Demed Medical (Sinaai, Belgium) and Ghent University (Ghent, Belgium). He is an editorial board member and director for the *British Journal of Anaesthesia*. Dr. Eleveld received travel from Becton Dickinson and is an editorial board member of Anesthesiology. Dr. Pesic and Dr. Stöhr were employees of Paion (Aachen, Germany) at the time of the conduct of this study. Dr. Colin declares that over the last 3 yr, his research group has been involved in contract research for Paion UK Ltd. (London, United Kingdom) and Acacia Pharma Ltd. (Cambridge, United Kingdom). The other authors declare no competing interests.

### Reproducible Science

Full protocol available at: p.j.colin@umcg.nl. Raw data available at: p.j.colin@umcg.nl.

## Supplemental Digital Content

Supplemental Digital Content 1. Sequence of interventions, definition MOAAS categories, https://links.lww.com/ALN/D786

Supplemental Digital Content 2. Clinical trial optimization, https://links.lww.com/ALN/D787

Supplemental Digital Content 3. Hemodynamic effects, https://links.lww.com/ALN/D788

Supplemental Digital Content 4. Evaluation of accuracy of TCI systems, https://links.lww.com/ALN/D789

Supplemental Digital Content 5. Development population pharmacokinetic model, https://links.lww.com/ALN/D790

Supplemental Digital Content 6. Diagnostics population pharmacodynamic models, https://links.lww.com/ALN/D791

Supplemental Digital Content 7. Exposure–response simulations at 60 min, https://links.lww.com/ALN/D792

## Supplementary Material
