# Supplementary material for: Influence of Remifentanil on the Pharmacokinetics and Pharmacodynamics of Remimazolam in Healthy Volunteers
Source: Anesthesiology. 2025 Jan 15;142(4):666–79. doi: 10.1097/ALN.0000000000005348 (PMC11892992; doi:10.1097/ALN.0000000000005348)

Supplemental Digital Content 1

Table 1. *Individual categories of the Modified Observers' Assessment of Alertness and Sedation Score*

Score	Response
5	Responds readily to name spoken in normal tone
4	Lethargic response to name spoken in normal tone
3	Responds only after name is called loudly and/or repeatedly
2	Responds only after mild prodding or shaking
1	Responds only after painful trapezius squeeze
0	No response after painful trapezius squeeze

Figure 1. *Remimazolam and remifentanil concentration steps of the step-up and step-down infusion protocol. infusion regiments. IIIa * and IIIb * where applicable before the evaluation of the first 6 volunteers.*

Period	Dosing & Recovery			Volunteers
I	**Remimazolam step-up** 150, 300, 400, 800, 1300, 2000 ng/mL	**Remimazolam step-down** 1300, 800, 400, 300, 150 ng/mL	Recovery	N=24
II	**Remimazolam step-up** 150, 300, 400, 800, 1300, 2000 ng/mL	**Remimazolam step-down** 1300, 800, 400, 300, 150 ng/mL	Recovery	N=24
	Constant infusion of remifentanil Cp = 0.5 ng/mL			
IIIa	**Remimazolam step-up** 125, 225, 275, 525, 925, 1700 ng/mL	**Remimazolam step-down** 925, 525, 275, 225, 125 ng/mL	Recovery	N=12
	Constant infusion of remifentanil Cp = 0.1 ng/mL			
OR				
IIIb	**Remimazolam step-up** 50, 100, 125, 225, 400, 1300 ng/mL	**Remimazolam step-down** 400, 225, 125, 100, 50 ng/mL	Recovery	N=12
	Constant infusion of remifentanil Cp = 1.0 ng/mL			
IIIa*	**Remimazolam step-up** 100, 200, 250, 500, 850, 1350 ng/mL	**Remimazolam step-down** 850, 500, 250, 200, 100 ng/mL	Recovery	N=12
	Constant infusion of remifentanil Cp = 2.0 ng/mL			
OR				
IIIb*	**Remimazolam step-up** 75, 150, 200, 400, 650, 1000 ng/mL	**Remimazolam step-down** 650, 400, 200, 150, 75 ng/mL	Recovery	N=12
	Constant infusion of remifentanil Cp = 4.0 ng/mL			

Figure 2. *Overview of interventions during each step of the step-up and step-down infusion protocol.*

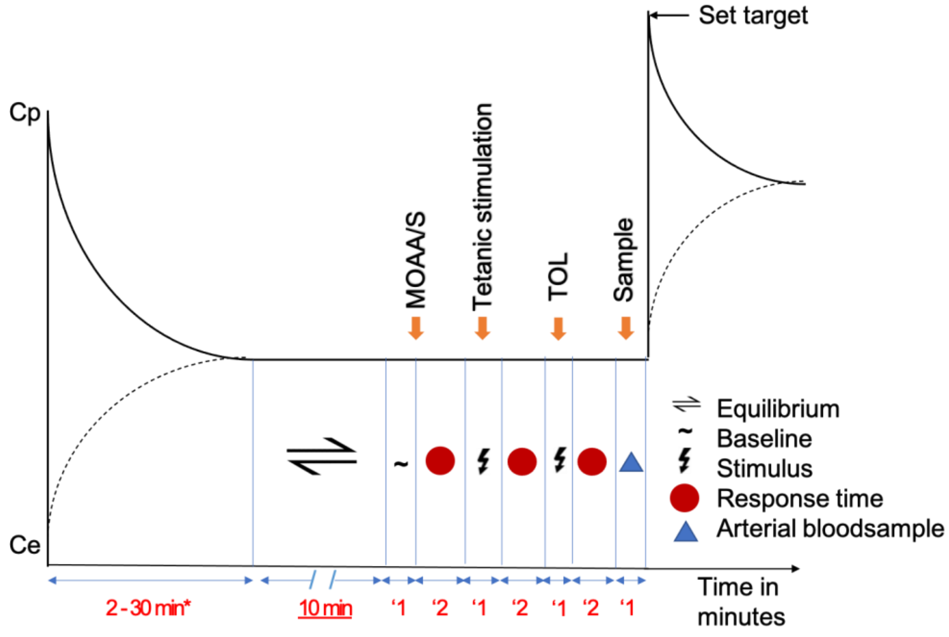

Figure 3. *Overview of remimazolam pharmacokinetic sampling scheme.*

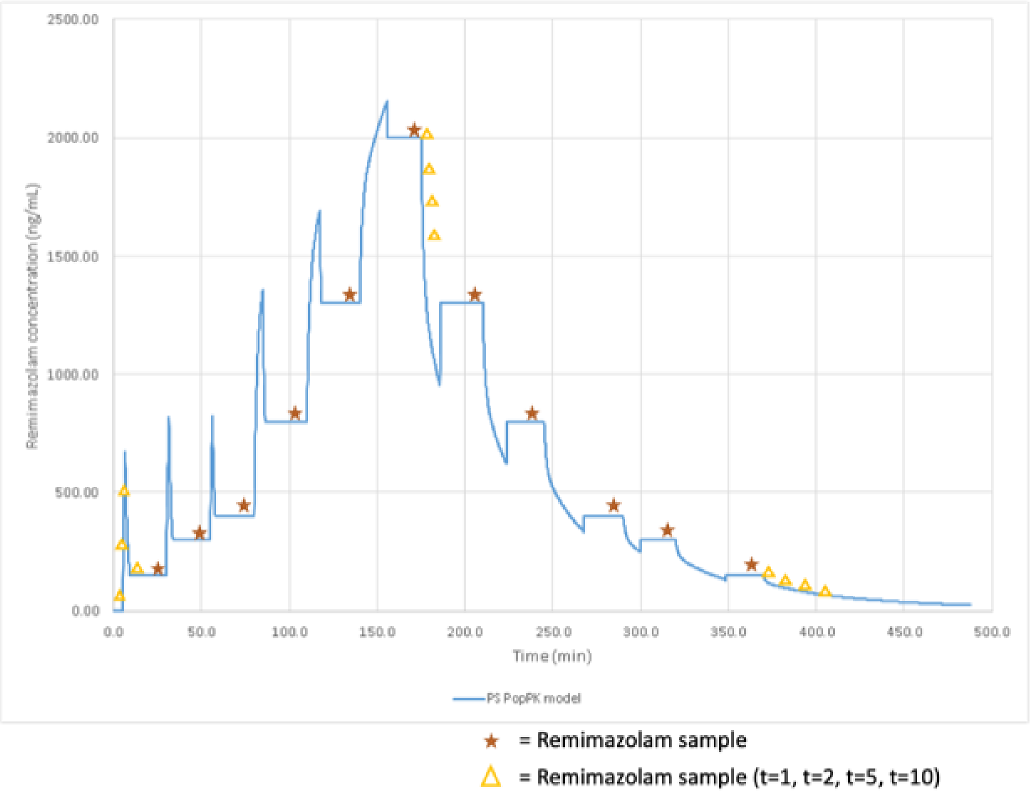

Supplement: Supplementary file 1 [file aln-142-666-s001.pdf]
