# Supplementary material for: Influence of Remifentanil on the Pharmacokinetics and Pharmacodynamics of Remimazolam in Healthy Volunteers
Source: Anesthesiology. 2025 Jan 15;142(4):666–79. doi: 10.1097/ALN.0000000000005348 (PMC11892992; doi:10.1097/ALN.0000000000005348)
Supplement: Supplementary file 2 [file aln-142-666-s002.pdf]

Supplemental digital content 2

Clinical trial design is of crucial importance when studying the interaction between different drugs. Design elements such as (i) the number of volunteers to include, (ii) the dose levels (or the target effect-site concentrations) of the different drugs, (iii) the number of dose levels and (iv) the allocation of volunteers across drugs and dose levels are of particular importance and can, if chosen poorly, result in an uninformative clinical trial.

The primary objective of our trial was to characterize the dose-exposure-response relationship of remimazolam when targeting MOAA/S 2 and 3 and the influence of remifentanil on this relationship. To maximize the information content a simulation study was set up to guide the design of the trial.

To inform our simulations we used a proportional odds logistic regression model for MOAA/S. The parameters for this model were informed by previous work by Zhou and colleagues[1] and Schütler and colleagues[2]. From the discrete-time Markov model presented by Zhou and colleagues[1] we derived, using simulations, the concentrations of remimazolam resulting in a 50 % probability (EC_{50}) for MOAA/S ≤4, MOAA/S ≤3 and MOAA/S ≤1. For this we assumed stationarity and ignored the components in the model describing the opioid – remimazolam interaction. Next, from the model presented by Schütler and colleagues we extracted the EC_{50}s for MOAA/S ≤2 and MOAA/S = 0 and the Hill coefficient (γ) describing the steepness of the exposure-response relationship. The resulting composite proportional odds logistic regression model was supplemented with a hierarchical interaction term (as described by Kuizenga and colleagues[3]) assuming that at 2.0 ng.mL^{-1} remifentanil the remimazolam EC_{50}s were reduced by 50% and assuming a Hill coefficient for the remifentanil-remimazolam interaction of 1.

Based on this model different trial designs were simulated. Each trial design was simulated 1,000 times. Next, the model was re-fitted to the simulated data sets and the parameter estimates were extracted. Trial designs were compared on the basis of the expected bias of the parameter estimates, the type I error (failure to identify between-subject variability in EC_{50}) and the eta-shrinkage on between-subject variability on EC_{50}.

The starting point for the optimization was a comparison between three designs: "Design 1" in Table 1 was a trial design previously described by Kuizenga and coworkers[3], "Design 2" was the "criss-cross design" as proposed by Short and colleagues[4] and "Design 3" was a single-session cross-over design as described by Bouillon and colleagues[5]. The shrinkage on the estimate for the between-subject variability in the EC_{50} for remimazolam was acceptable in designs 1 and 3, with a small median shrinkage across simulations and >80 % of simulations have a shrinkage <20 %, but was unacceptably high for design 2. Although design 3 and 1 performed similarly, design 3 was not pursued further due to the impracticality of doing the cross-over within a single study period. Further modifications of trial design 1, with the intention of reducing the type I error on the parameter describing the between-subject variability on the EC_{50} for remifentanil, led to the final design 4.

According to the final design, age- and gender-stratified volunteers receive a "step-up" and "step-down" remimazolam dosing regimen during period 1 in the absence of remifentanil. In period 2, after an appropriate washout (> 1 week), these volunteers receive the same remimazolam "step-up" and "step-down" dosing regimen with a 0.5 ng.mL^{-1} remifentanil background. Prior to period 3, volunteers are

randomized across 2 treatment arms and receive a modified remimazolam dosing regimen with a remifentanil background of 2 or 4 ng.mL^{-1}, respectively. Based on this design, we found that, in order to obtain reliable estimates of the dose-exposure-response for MOAA/S and the strength of the remifentanil-remimazolam interaction at least 24 volunteers should be included in this two-arm, three-period cross-over study.

References

1. Zhou J, Curd L, Lohmer LRL, et al. A population pharmacodynamic Markov mixed-effects model for determining remimazolam-induced sedation when co-administered with fentanyl in procedural sedation. Clin Transl Sci. 2021;14(4):1554-1565.

2. Schüttler J, Eisenried A, Lerch M, Fechner J, Jeleazcov C, Ihmsen H. Pharmacokinetics and Pharmacodynamics of Remimazolam (CNS 7056) after Continuous Infusion in Healthy Male Volunteers: Part I. Pharmacokinetics and Clinical Pharmacodynamics. Anesthesiology. 2020;132(4):636-651.

3. Kuizenga MH, Colin PJ, Reyntjens KMEM, Touw DJ, Nalbat H, Knotnerus FH, Vereecke HEM, Struys MMRF. Population Pharmacodynamics of Propofol and Sevoflurane in Healthy Volunteers Using a Clinical Score and the Patient State Index: A Crossover Study. Anesthesiology. 2019 Dec;131(6):1223-1238.

4. Short TG, Ho TY, Minto CF, Schnider TW, Shafer SL; Efficient Trial Design for Eliciting a Pharmacokinetic–Pharmacodynamic Model–based Response Surface Describing the Interaction between Two Intravenous Anesthetic Drugs. Anesthesiology 2002 Feb; 96(2):400–408.

5. Bouillon TW, Bruhn J, Radulescu L, Andresen C, Shafer TJ, Cohane C, Shafer SL; Pharmacodynamic Interaction between Propofol and Remifentanil Regarding Hypnosis, Tolerance of Laryngoscopy, Bispectral Index, and Electroencephalographic Approximate Entropy. Anesthesiology 2004 Jun; 100(6):1353–1372.

Table 1

	Design 1	Design 2	Design 3	Design 4
**Design features**				
# of patients	30	60	60	24
# of periods	2	1	1	3
Period 1 RMZ TCI targets	150, 300, 400, 800, 1300,2000	RMZ targets: 150, 300, 400, 800, 1300,2000 (50% reduced for 2 highest RME groups) with RME 0 ng/mL (n=6), 0.5 (n=6), 2 (n=6), 3 (n=6), 4 (n=6) & RME targets: 0.5, 1.0, 2.0, 2.5, 3.0, 4.0 (50% reduced for 2 highest RMZ groups) with RMZ 150 ng/mL (n=6), 300 (n=6), 400 (n=6), 800 (n=6), 1300 (n=6)	150, 300, 400, 800, 1300,2000	150, 300, 400, 800, 1300,2000
Period 2 RMZ TCI targets	150, 300, 400, 800, 1300,2000<br>100, 200, 250, 500, 850, 1350<br>75, 150, 200, 400, 650, 1000		150, 300, 400, 800, 1300,2000<br>100, 200, 250, 500, 850, 1350<br>75, 150, 200, 400, 650, 1000	150, 300, 400, 800, 1300, 2000<br>100, 200, 250, 500, 850, 1350<br>75, 150, 200, 400, 650, 1000
Period 2 RME TCI targets (# of patients)	0.5 (10)<br>2.0 (10)<br>4.0 (10)		0.5 (20)<br>2.0 (20)<br>4.0 (20)	0.5 (24)<br>2.0 (12)<br>4.0 (12)
**Bias (%; median, 5th and 95th quantile) & Type I error [%]**				
EC_{50} MOAA/S ≤4	0.0 (-16; 18)	0.0 (-21; 23)	0.7 (-13; 14)	-0.7 (-18; 20)
ΔEC_{50} MOAA/S 4-3	-0.9 (-21; 23)	-0.7 (-32; 31)	-0.8 (-20; 20)	-1.2 (-23; 24)
ΔEC_{50} MOAA/S 3-2	0.0 (-21; 23)	0.0 (-27; 31)	0.8 (-17; 21)	-0.8 (-21; 22)
ΔEC_{50} MOAA/S 2-1	0.0 (-21, 26)	-0.8 (-29; 35)	-0.4 (-22; 23)	0.0 (-22; 26)
ΔEC_{50} MOAA/S 1-0	-1.0 (-17; 18)	-0.5 (-22; 28)	-0.6 (-14; 15)	-1.6 (-19; 20)
Hill coefficient RMZ	-4.2 (-15; 9.2)	-4.7 (-17; 11)	-4.2 (-14; 5.6)	-4.7 (-15; 7.8)
EC_{50} RME	0.0 (-21; 23)	1.5 (-36; 46)	0.2 (-19; 20)	0.5 (-19; 23)
Hill coefficient RME	2.0 (-30; 44)	3.0 (-31; 55)	2.0 (-25; 39)	4.0 (-18; 34)
BSV EC_{50} RMZ	-4.9 (-44; 44) [0.0]	-5.1 (-47; 46) [0.0]	-1.8 (-33; 35) [0.0]	-6.5 (-45; 51) [0.0]
BSV EC_{50} RME	-3.9 (-100; 285) [13]	-34 (-100; 415) [40]	-3.4 (-100; 272) [18]	13 (-100; 199) [8.3]
**Eta-shrinkage (%; median & [percentage of cases <20 %])**				
BSV EC_{50} RMZ	5.6 (82)	17 (58)	7.8 (85)	5.0 (84)
BSV EC_{50} RME	43 (29)	45 (29)	49 (21)	27 (42)

RMZ: remimazolam; RME: remifentanil; BSV: Between-subject variability
