# Supplementary material for: Influence of Remifentanil on the Pharmacokinetics and Pharmacodynamics of Remimazolam in Healthy Volunteers
Source: Anesthesiology. 2025 Jan 15;142(4):666–79. doi: 10.1097/ALN.0000000000005348 (PMC11892992; doi:10.1097/ALN.0000000000005348)

Supplemental Digital Content 3

Table 1. Haemodynamic and respiratory variables (mean [95% confidence interval]) at each target concentration step – Session 1

TCI target (ng.mL^{-1})	HR (bpm)	MAP (mm Hg)	SpO_2 (%)	$EtCO_2$ (mm Hg)
0	66.0 [60.8; 71.2]	90.8 [83.2; 98.4]	98.8 [98.0; 99.7]	23.5 [13.1; 34.0]
150	66.4 [63.5; 69.3]	81.4 [78.1; 84.7]	99.2 [98.8; 99.5]	26.4 [22.1; 30.7]
300	72.2 [67.6; 76.8]	71.3 [67.0; 75.6]	97.6 [96.7; 98.5]	23.6 [16.3; 30.8]
400	74.1 [69.2; 79.1]	70.6 [66.5; 74.7]	97.4 [96.8; 98.1]	25.8 [18.8; 32.8]
800	80.7 [74.6; 86.7]	69.9 [65.2; 74.6]	97.3 [96.4; 98.2]	23.0 [15.9; 30.1]
1300	83.6 [77.7; 89.4]	70.0 [66.1; 74.0]	97.1 [96.2; 98.1]	23.2 [15.7; 30.6]
2000	83.2 [77.2; 89.1]	69.3 [65.8; 72.9]	97.3 [96.4; 98.2]	25.2 [18.8; 31.5]
1300	80.0 [74.5; 85.6]	75.6 [71.5; 79.7]	97.8 [96.9; 98.8]	24.5 [18.1; 30.9]
800	76.5 [71.3; 81.7]	82.8 [78.0; 87.5]	98.8 [98.1; 99.5]	24.3 [18.1; 30.6]
400	74.4 [69.4; 79.4]	86.0 [80.4; 91.6]	99.5 [99.1; 99.9]	24.9 [18.9; 30.8]
300	73.2 [68.6; 77.7]	84.6 [79.6; 89.5]	99.5 [99.1; 99.8]	23.2 [16.9; 29.6]
150	71.4 [66.8; 76.0]	85.7 [80.8; 90.7]	99.1 [98.7; 99.6]	24.5 [17.5; 31.5]
0	72.3 [69.7; 74.9]	83.2 [78.3; 88.1]	99.2 [98.9; 99.4]	22.6 [18.6; 26.5]

Table 2. Haemodynamic and respiratory variables (mean [95% confidence interval]) at each target concentration step – Session 2 – 0.5 ng/mL remifentanil

TCI target (ng.mL^{-1})	HR (bpm)	MAP (mm Hg)	SpO_2 (%)	$EtCO_2$ (mm Hg)
0	67.4 [61.8; 73.0]	88.6 [84.5; 92.6]	98.0 [95.8; 100.0]	25.8 [13.6; 37.9]
150	65.6 [62.0; 69.3]	77.6 [75.0; 80.3]	97.6 [97.0; 98.1]	32.3 [28.8; 35.9]
300	68.7 [63.8; 73.6]	66.8 [63.3; 70.3]	96.5 [95.6; 97.4]	32.1 [26.8; 37.3]
400	70.5 [65.9; 75.2]	70.0 [66.4; 73.6]	97.0 [96.3; 97.8]	33.9 [28.4; 39.5]
800	75.0 [69.7; 80.4]	67.8 [62.8; 72.7]	96.7 [96.0; 97.5]	35.6 [30.5; 40.8]
1300	75.7 [69.9; 81.6]	65.7 [61.1; 70.4]	96.6 [95.8; 97.4]	34.6 [28.8; 40.5]
2000	77.8 [71.2; 84.4]	68.8 [64.0; 73.5]	97.0 [96.3; 97.7]	35.5 [29.1; 41.8]
1300	75.1 [68.8; 81.5]	75.7 [72.0; 79.3]	97.8 [97.2; 98.4]	33.9 [27.9; 39.8]
800	72.3 [66.2; 78.4]	80.8 [76.6; 85.1]	98.6 [98.0; 99.3]	34.8 [28.8; 40.7]
400	68.2 [62.2; 74.1]	82.8 [79.5; 86.2]	99.1 [98.6; 99.6]	32.6 [27.1; 38.0]
300	67.8 [62.2; 73.3]	82.3 [78.9; 85.7]	99.0 [98.4; 99.5]	33.8 [28.9; 38.8]
150	70.4 [63.8; 77.0]	88.0 [83.7; 92.4]	98.9 [98.4; 99.4]	31.8 [26.4; 37.2]
0	73.8 [69.7; 78.0]	85.1 [82.2; 88.0]	99.0 [98.6; 99.4]	30.4 [26.7; 34.1]

Table 3. Haemodynamic and respiratory variables (mean [95% confidence interval]) at each target concentration step – Session 3 – 0.1 ng/mL remifentanil

TCI target (ng.mL^{-1})	HR (bpm)	MAP (mm Hg)	SpO_2 (%)	$EtCO_2$ (mm Hg)
0	61.1 [56.2; 66.0]	89.4 [83.6; 95.1]	98.7 [97.5; 99.9]	28.4 [11.9; 44.9]
125	61.4 [57.1; 65.7]	79.6 [76.4; 82.8]	98.8 [98.1; 99.5]	33.7 [28.0; 39.4]
225	66.0 [52.5; 79.5]	71.0 [65.2; 76.8]	97.9 [96.5; 99.4]	32.5 [19.6; 45.3]
275	67.3 [56.4; 78.3]	72.9 [67.4; 78.4]	97.4 [95.7; 99.2]	33.2 [22.1; 44.4]
525	74.2 [59.6; 88.9]	65.1 [59.6; 70.7]	96.6 [94.4; 98.8]	32.8 [18.2; 47.3]
925	78.2 [60.9; 95.6]	63.0 [56.9; 69.1]	97.5 [96.0; 98.9]	34.0 [21.2; 46.8]
1700	80.6 [62.2; 99.0]	65.1 [58.3; 71.9]	97.0 [95.3; 98.7]	36.1 [23.0; 49.3]
925	72.2 [57.7; 86.6]	73.2 [67.2; 79.3]	98.4 [97.2; 99.6]	33.9 [23.3; 44.5]
525	67.5 [54.9; 80.1]	82.8 [73.3; 92.2]	99.3 [98.4; 100.]	34.7 [26.0; 43.3]
275	61.9 [52.5; 71.3]	84.7 [79.5; 90.0]	99.0 [97.4; 101.]	32.6 [21.2; 44.0]
225	62.9 [54.2; 71.7]	83.4 [76.3; 90.5]	98.7 [97.0; 100.]	34.3 [24.4; 44.2]
125	63.4 [56.0; 70.7]	87.6 [82.1; 93.1]	98.6 [96.8; 100.]	33.4 [27.2; 39.6]
0	63.2 [54.0; 72.4]	85.0 [79.4; 90.6]	99.0 [97.8; 100.]	34.1 [28.3; 39.9]

Table 4. Haemodynamic and respiratory variables (mean [95% confidence interval]) at each target concentration step – Session 3 – 1.0 ng/mL remifentanil

TCI target (ng.mL^{-1})	HR (bpm)	MAP (mm Hg)	SpO_2 (%)	$EtCO_2$ (mm Hg)
0	69.2 [54.7; 83.8]	88.9 [79.3; 98.4]	99.7 [99.2; 100.]	39.0 [39.0; 39.0]
50	63.8 [58.6; 68.9]	78.3 [74.5; 82.0]	97.9 [97.2; 98.7]	37.5 [31.4; 43.7]
100	63.9 [55.4; 72.4]	72.9 [65.0; 80.8]	97.5 [95.9; 99.1]	36.9 [25.6; 48.2]
125	62.2 [55.8; 68.7]	67.9 [62.6; 73.2]	96.4 [94.2; 98.6]	38.4 [26.8; 49.9]
225	65.3 [58.8; 71.8]	63.8 [59.2; 68.3]	96.0 [94.7; 97.2]	39.8 [28.2; 51.3]
400	68.9 [60.7; 77.1]	59.2 [52.8; 65.7]	95.7 [94.1; 97.4]	39.7 [28.3; 51.1]
1300	74.9 [65.9; 83.9]	58.8 [53.5; 64.1]	96.3 [95.5; 97.2]	43.8 [32.1; 55.4]
400	65.3 [57.6; 73.0]	71.5 [65.2; 77.8]	98.6 [97.7; 99.4]	41.3 [32.4; 50.2]
225	67.8 [57.1; 78.4]	76.1 [70.0; 82.2]	99.1 [98.3; 100.]	41.2 [30.9; 51.4]
125	64.9 [56.1; 73.7]	75.4 [69.6; 81.1]	99.1 [98.3; 99.9]	38.8 [28.8; 48.8]
100	62.0 [53.1; 70.9]	74.0 [69.0; 79.0]	99.1 [97.9; 100.]	41.7 [33.4; 50.0]
50	62.0 [55.0; 69.0]	76.7 [70.8; 82.6]	98.8 [97.6; 99.9]	39.0 [30.7; 47.2]
0	65.6 [60.9; 70.4]	74.8 [70.1; 79.5]	98.7 [97.9; 99.5]	34.9 [28.8; 41.1]

Table 5. Haemodynamic and respiratory variables (mean [95% confidence interval]) at each target concentration step – Session 3 – 2.0 ng/mL remifentanil

TCI target (ng.mL^{-1})	HR (bpm)	MAP (mm Hg)	SpO_2 (%)	$EtCO_2$ (mm Hg)
0	64.4 [56.8; 72.0]	86.6 [75.7; 97.5]	98.7 [95.4; 102.]	33.5 [-155.; 222.]*
100	61.8 [58.3; 65.2]	77.4 [69.9; 84.8]	95.4 [92.8; 97.9]	32.9 [18.0; 47.8]
200	65.5 [60.6; 70.4]	64.5 [60.1; 68.9]	96.8 [92.0; 102.]	30.8 [-4.08; 65.6]
250	73.8 [48.4; 99.1]	65.2 [51.9; 78.6]	97.3 [92.3; 102.]	38.2 [5.89; 70.6]
500	72.0 [65.8; 78.2]	59.5 [56.3; 62.7]	98.6 [97.0; 100.]	43.2 [21.1; 65.4]
850	69.8 [64.1; 75.4]	57.8 [50.8; 64.7]	99.0 [96.9; 101.]	34.5 [15.9; 53.1]
1350	69.2 [60.8; 77.7]	64.8 [46.8; 82.7]	99.2 [98.0; 100.]	26.2 [1.31; 51.2]
850	65.0 [56.6; 73.4]	74.8 [61.6; 87.9]	99.8 [99.3; 100.]	23.7 [-2.32; 49.7]
500	64.8 [47.9; 81.6]	79.2 [75.8; 82.7]	100. [100.; 100.]	32.8 [16.5; 49.0]
250	65.0 [55.9; 74.1]	84.8 [69.0; 101.]	99.9 [99.4; 100.]	34.4 [18.3; 50.5]
200	70.2 [58.2; 82.3]	82.0 [61.1; 103.]	99.2 [96.4; 102.]	39.0 [19.7; 58.3]
100	71.0 [57.4; 84.6]	83.0 [76.8; 89.2]	99.0 [96.8; 101.]	43.0 [21.9; 64.1]
0	74.1 [70.0; 78.3]	83.7 [79.0; 88.3]	99.6 [98.9; 100.]	29.6 [19.1; 40.1]

* Only 2 measurements available

Table 6. Haemodynamic and respiratory variables (mean [95% confidence interval]) at each target concentration step – Session 3 – 4.0 ng/mL remifentanil

TCI target (ng.mL^{-1})	HR (bpm)	MAP (mm Hg)	SpO_2 (%)	$EtCO_2$ (mm Hg)
0	71.0*	81.0*	96.6*	**
75	70.2 [51.1; 89.4]	85.8 [58.4; 113.]	96.9 [93.5; 100.]	32.2 [-8.77; 73.3]
150	64.0 [46.0; 82.0]	66.5 [-50.3; 183.]	98.0 [88.2; 108.]	41.5 [-219.; 302.]
200	70.0 [34.1; 106.]	57.5 [-131.; 246.]	97.8 [95.1; 100.]	49.0 [-40.8; 139.]
400	64.5 [1.61; 127.]	64.0 [46.0; 82.0]	99.0 [95.4; 103.]	28.2 [-111.; 168.]
650	62.0 [44.0; 80.0]	64.5 [-16.4; 145.]	98.8 [96.1; 101.]	30.2 [-55.1; 116.]
1000	58.0 [40.0; 76.0]	66.0 [-5.88; 138.]	98.4 [94.0; 103.]	26.0 [-63.8; 116.]
650	57.0 [57.0; 57.0]	69.0 [-2.88; 141.]	97.8 [87.9; 108.]	23.0 [-157.; 203.]
400	56.0 [38.0; 74.0]	74.0 [38.1; 110.]	99.3 [96.1; 102.]	26.0 [-118.; 170.]
200	65.5 [-87.2; 218.]	82.0 [64.0; 100.]	95.2 [9.90; 181.]	35.0 [-235.; 305.]
150	66.5 [-32.3; 165.]	75.5 [-59.3; 210.]	99.6 [91.5; 108.]	36.0 [-234.; 306.]
75	71.0 [35.1; 107.]	80.0 [80.0; 80.0]	98.2 [66.8; 130.]	40.0 [-319.; 399.]
0	78.5 [66.6; 90.4]	80.8 [77.0; 84.5]	98.8 [96.7; 101.]	39.5 [13.8; 65.2]

* No 95% CI is reported because there was only 1 observation available; ** No measurements available

Figure 1: SpO2 versus time: Solid black lines denote the arterial SpO2 (%), vertical red lines denote the timepoints when a 5 mg bolus of ephedrine was administered, vertical green, blue or yellow lines denote timepoints when the pharmacodynamic endpoints were assessed (respectively: tolerance to laryngoscopy, MOAA/S and tolerance to a tetanic stimulus).

ID 1 – Session 1

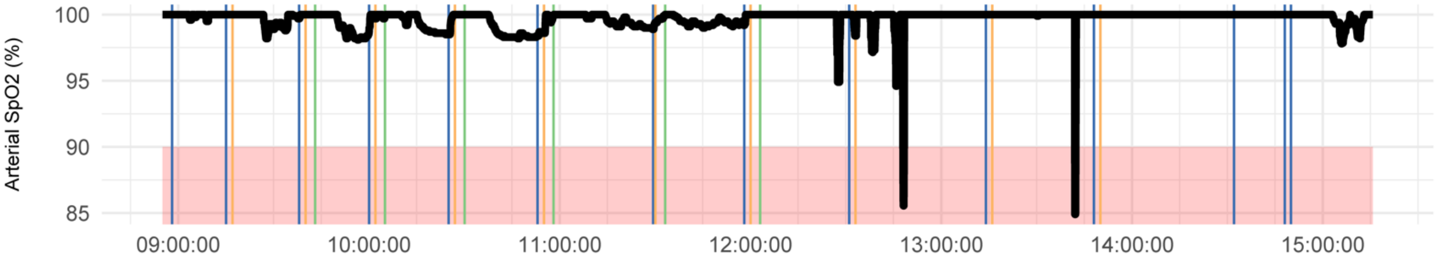

ID 1 – Session 2

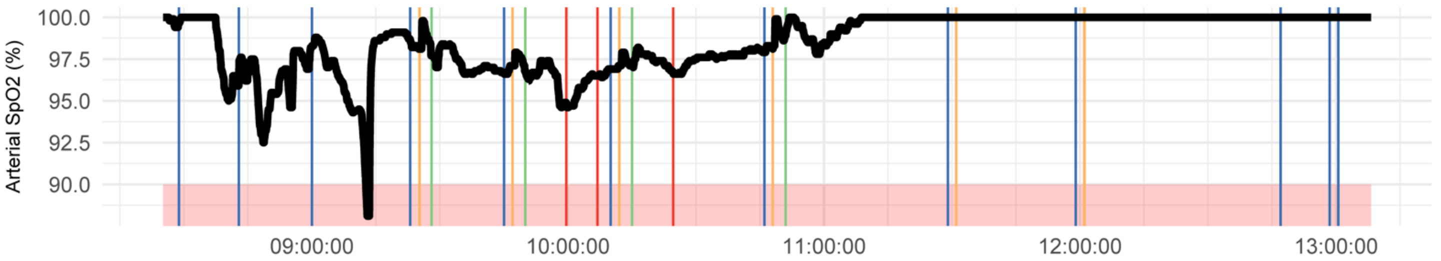

ID 1 – Session 3

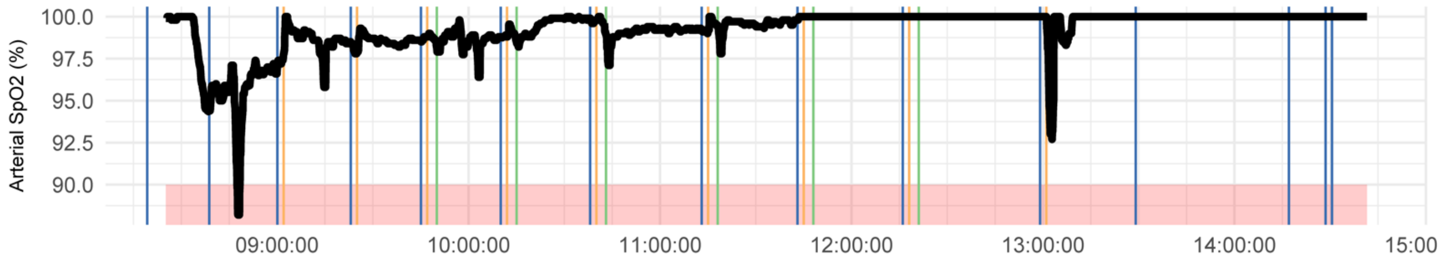

ID 2 – Session 1

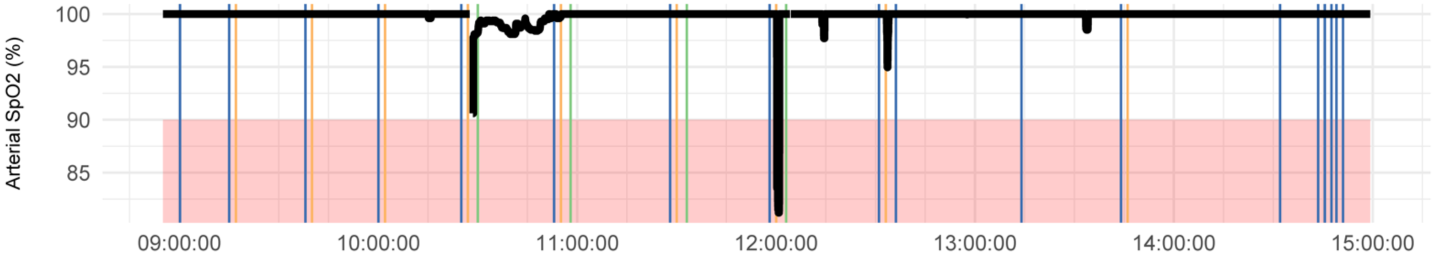

ID 2 – Session 2

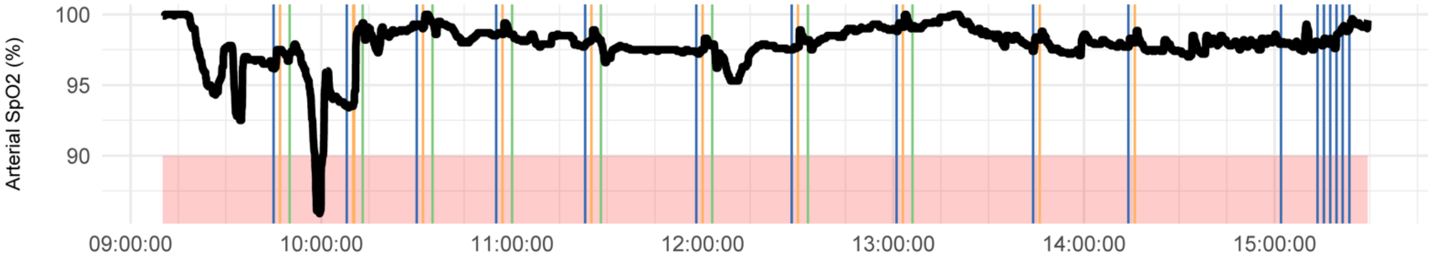

ID 2 – Session 3

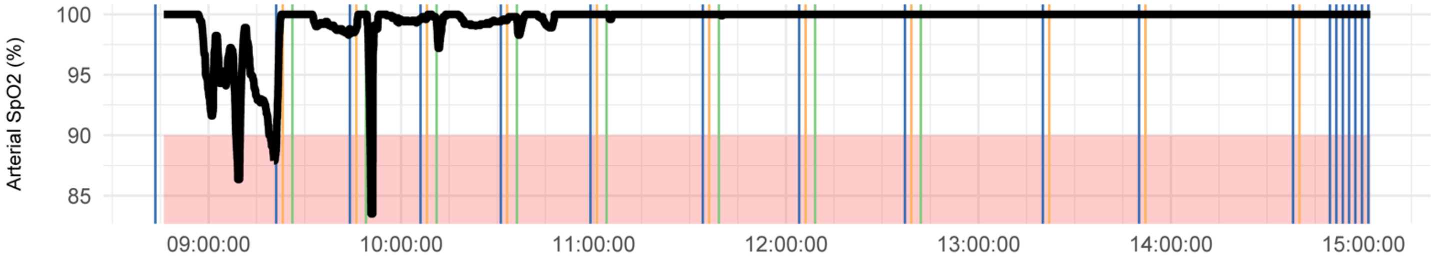

ID 3 – Session 1

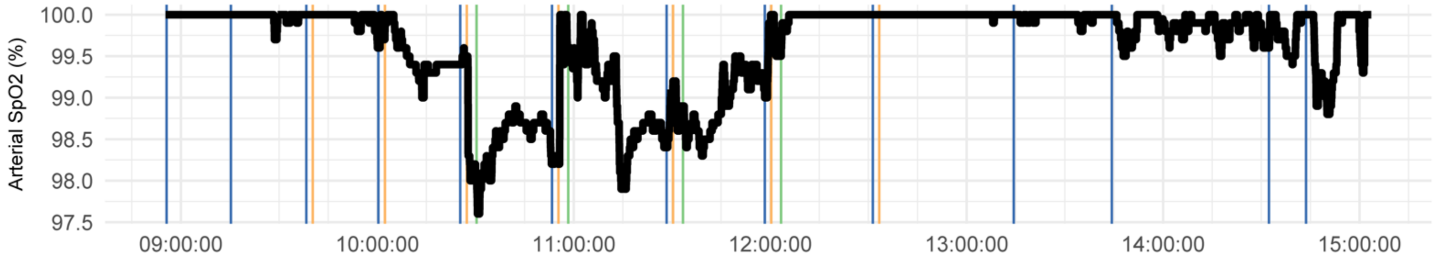

ID 3 – Session 2

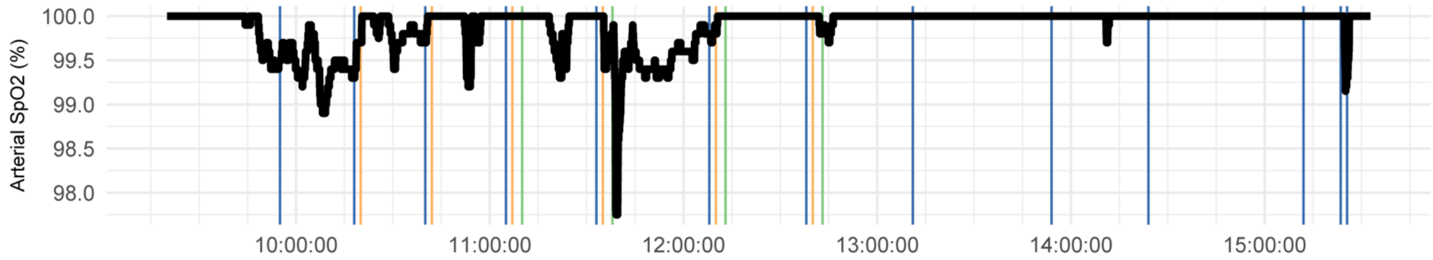

ID 3 – Session 3

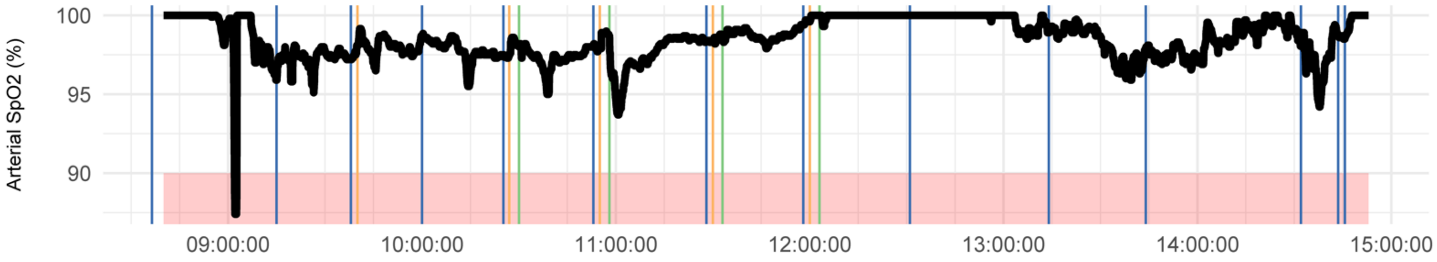

ID 4 – Session 1

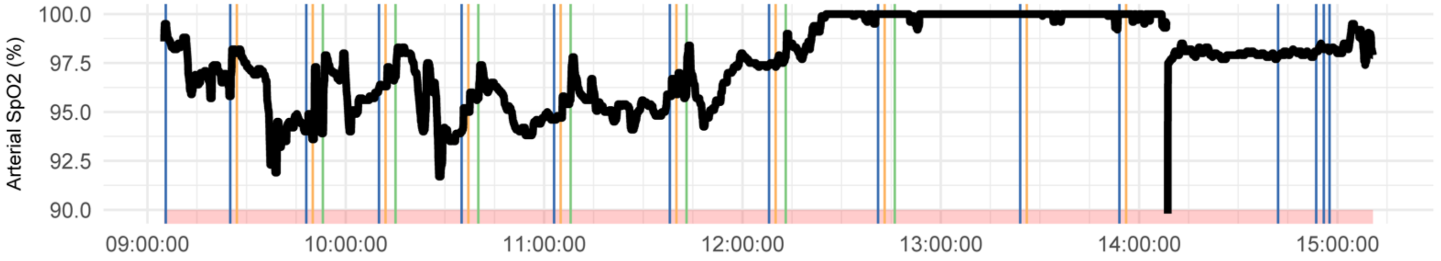

ID 4 – Session 2

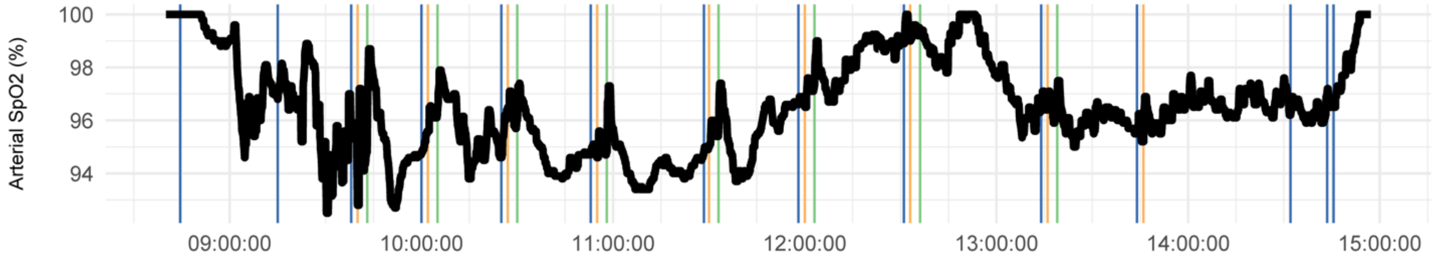

ID 4 – Session 3

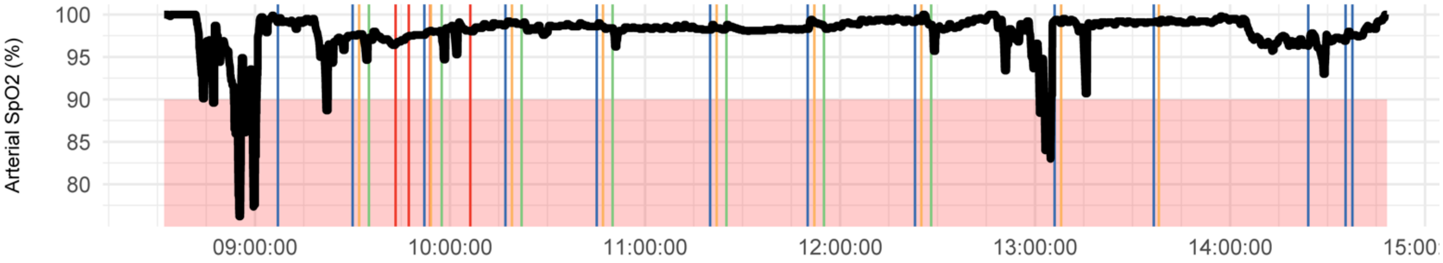

ID 5 – Session 1

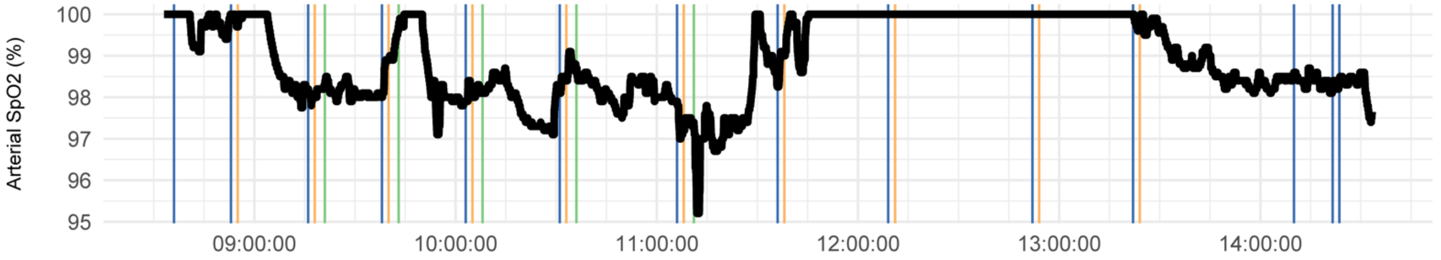

ID 5 – Session 2

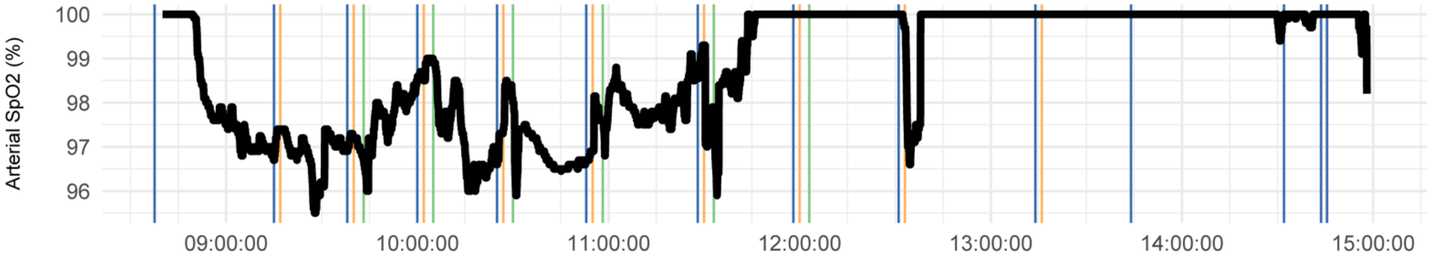

ID 5 – Session 3

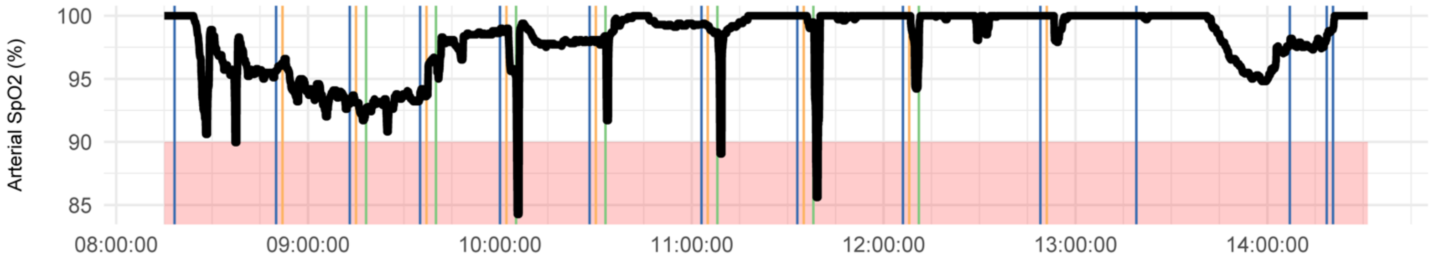

ID 6 – Session 1

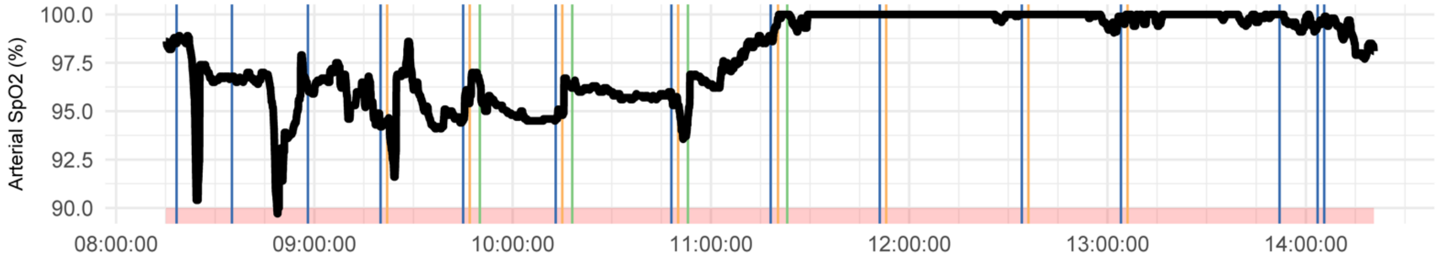

ID 6 – Session 2

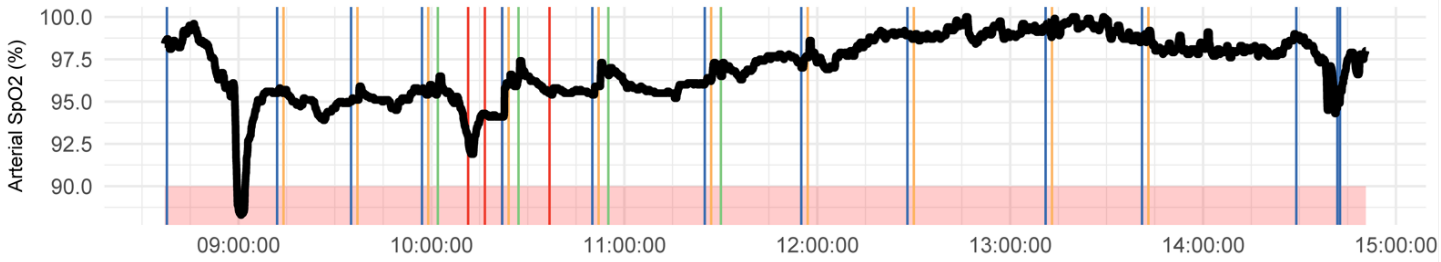

ID 6 – Session 3

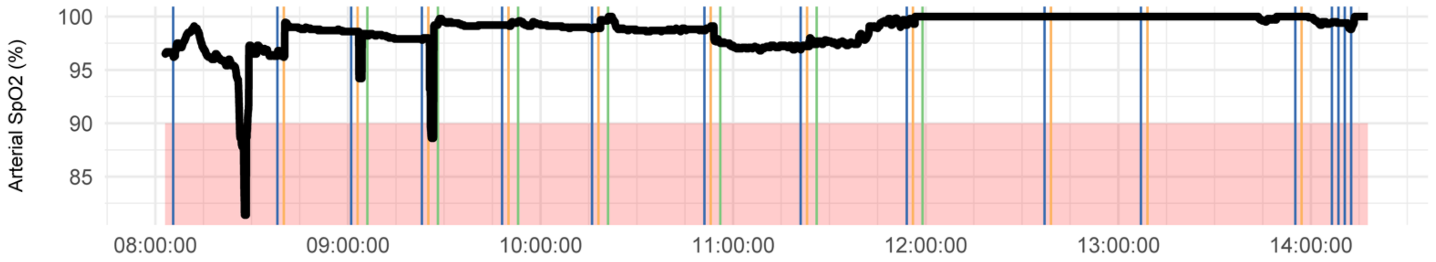

ID 8 – Session 1

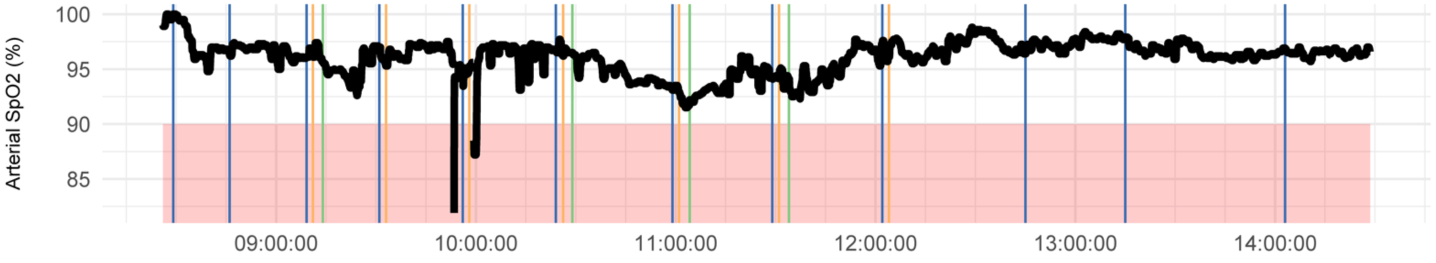

ID 8 – Session 2

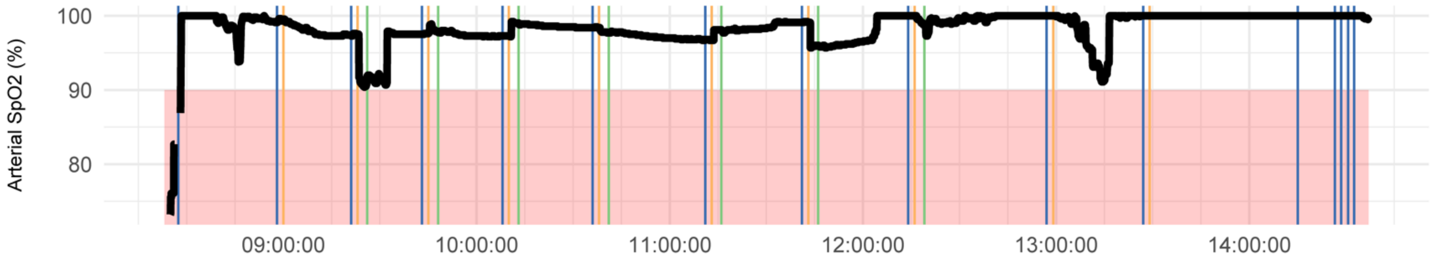

ID 8 – Session 3

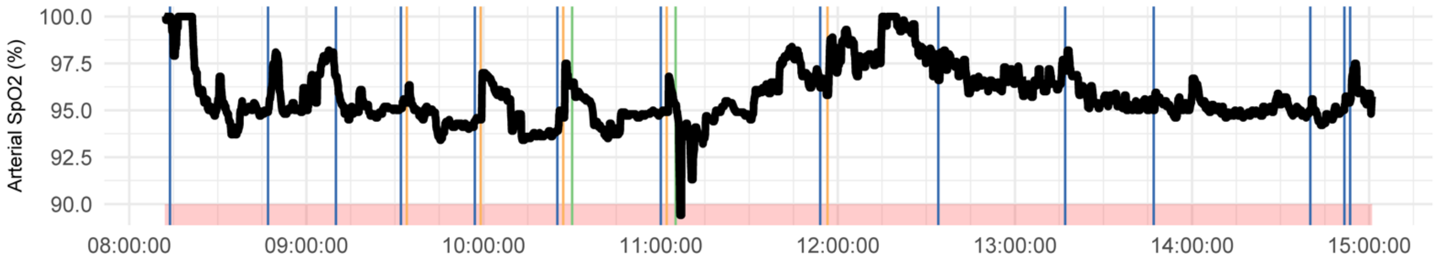

ID 9 – Session 1

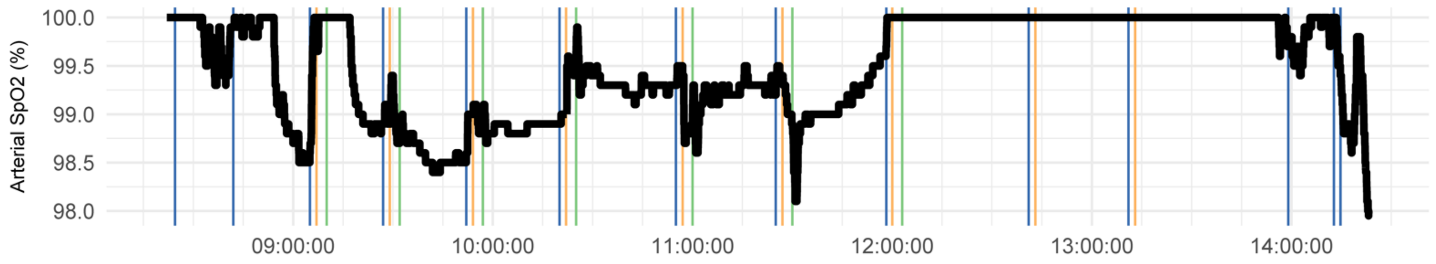

ID 9 – Session 2

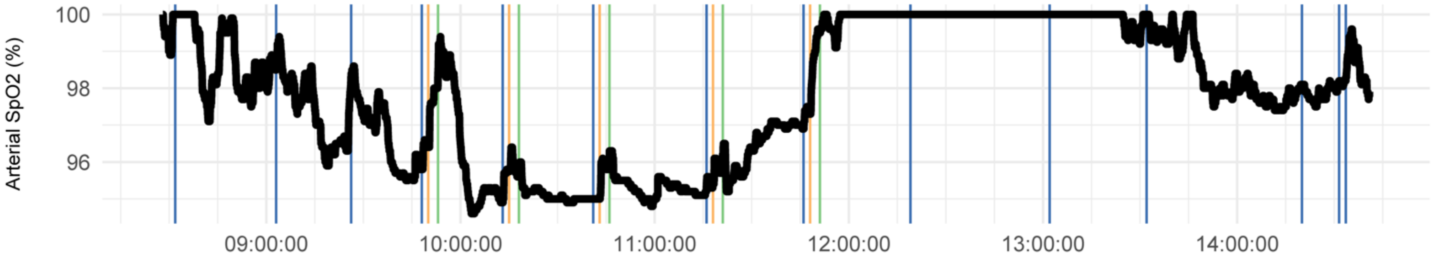

ID 9 – Session 3

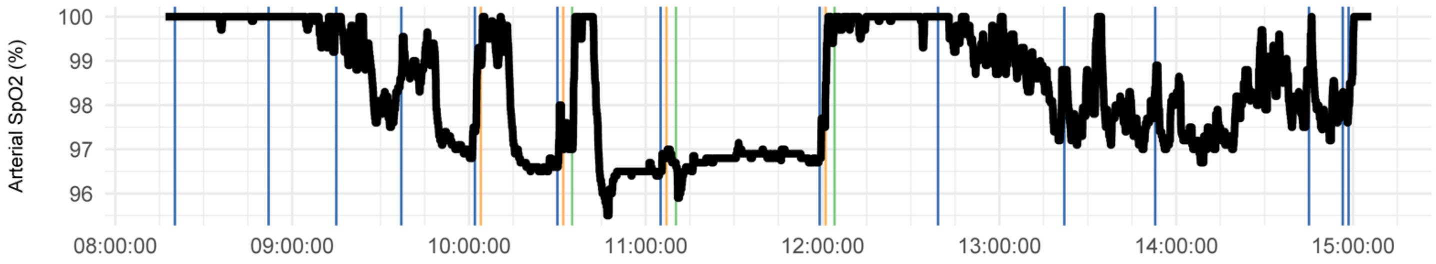

ID 10 – Session 1

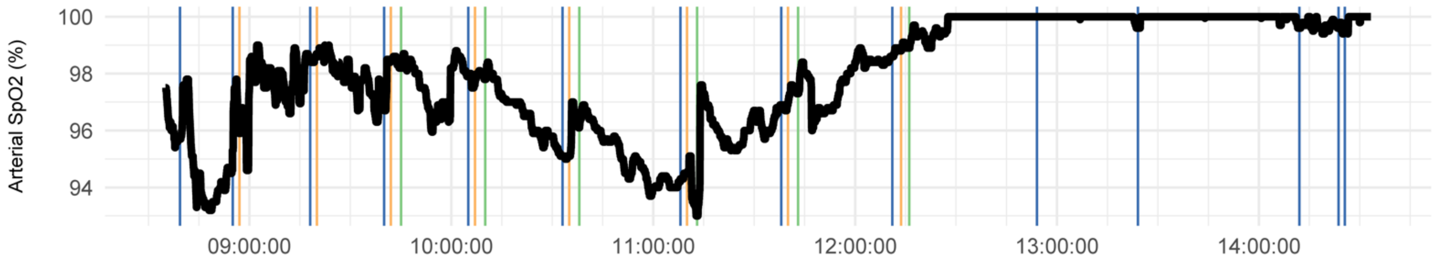

ID 10 – Session 2

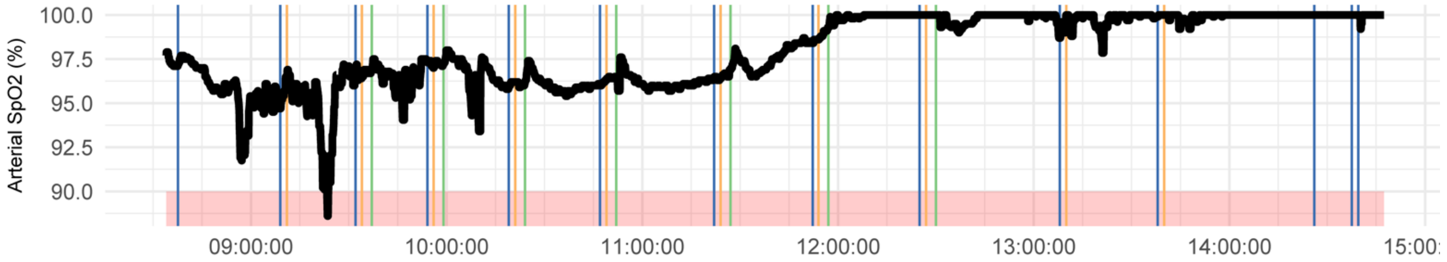

ID 10 – Session 3

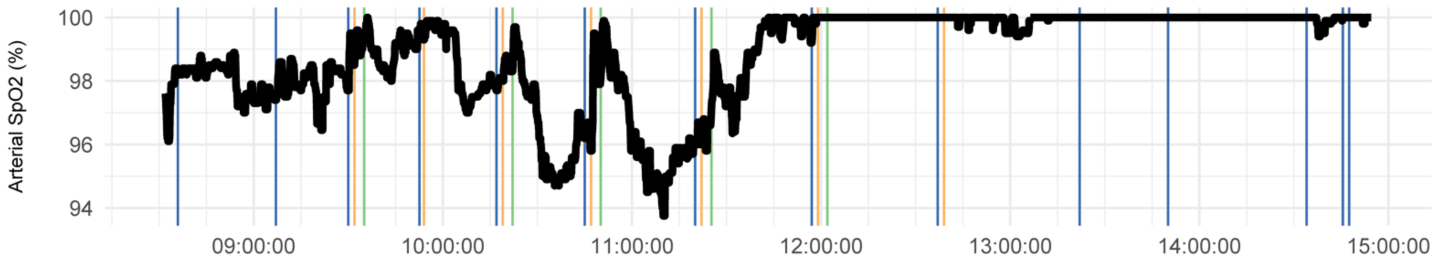

ID 11 – Session 1

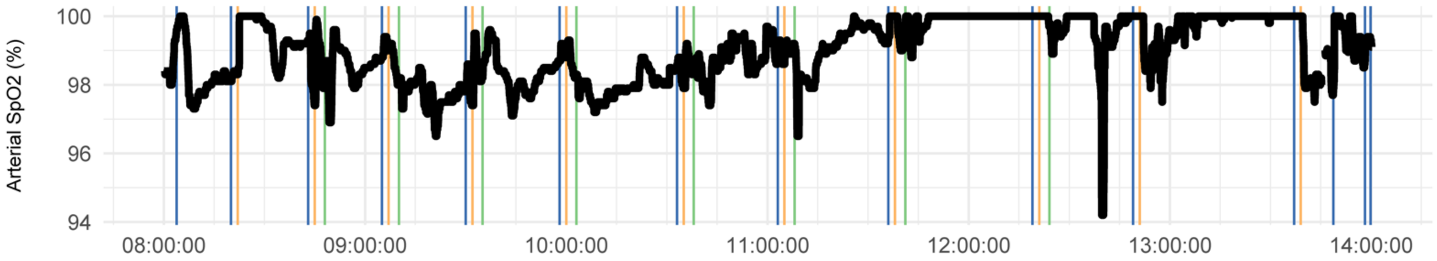

ID 11 – Session 2

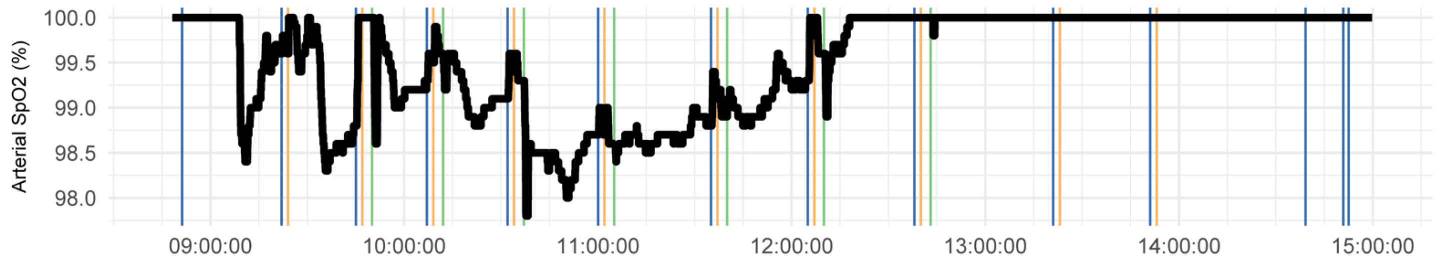

ID 11 – Session 3

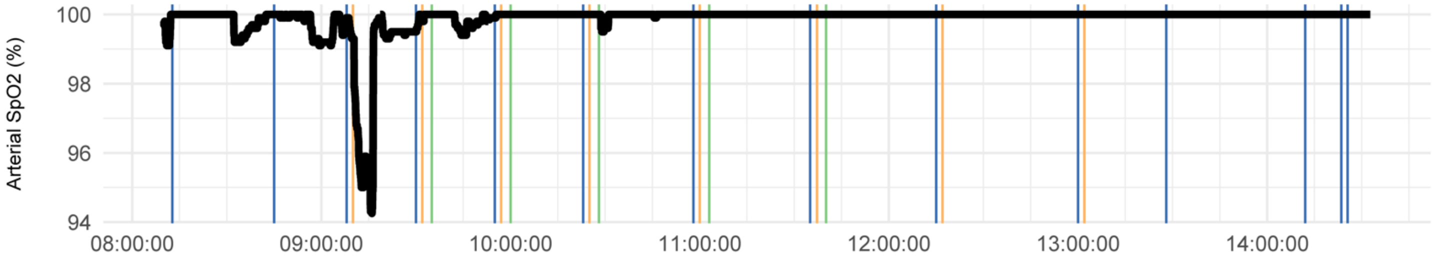

ID 12 – Session 1

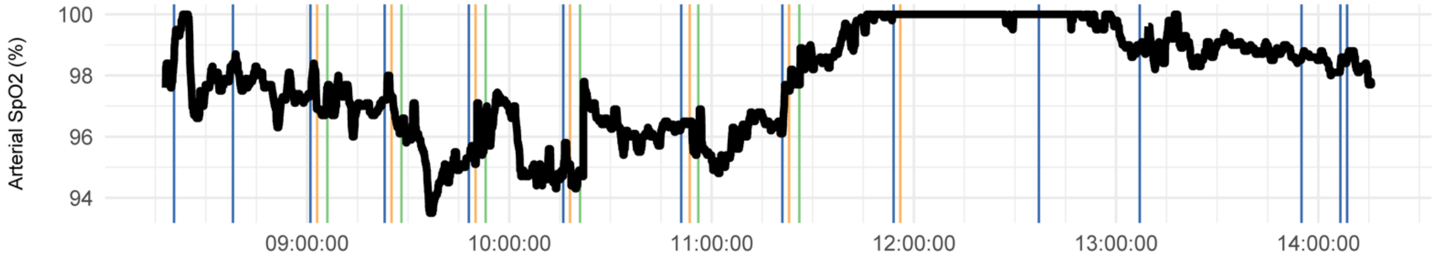

ID 12 – Session 2

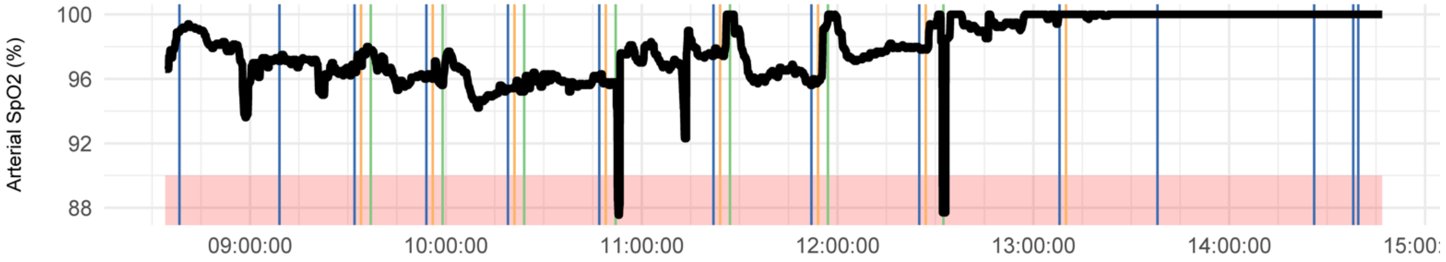

ID 12 – Session 3

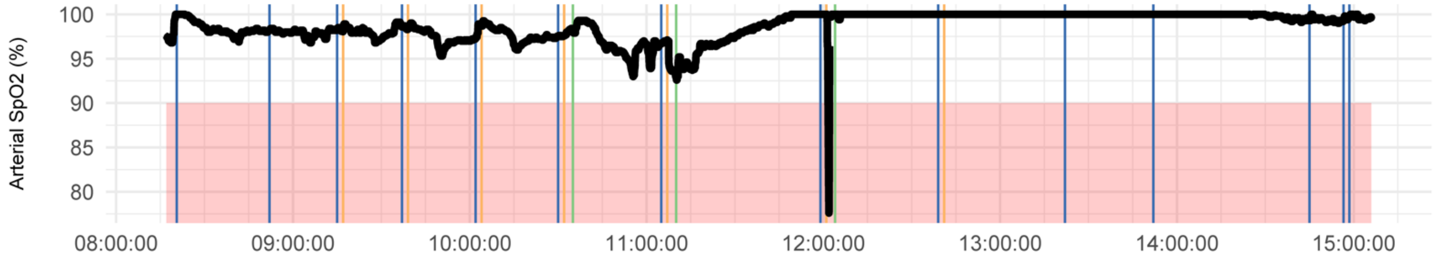

ID 13 – Session 1

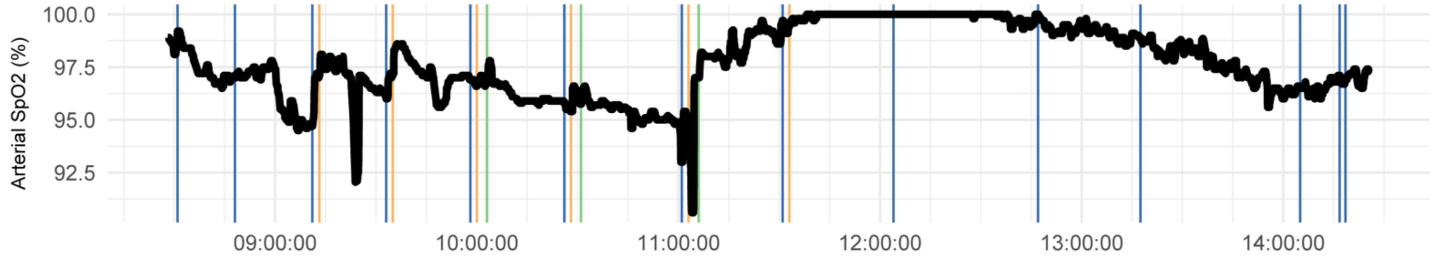

ID 13 – Session 2

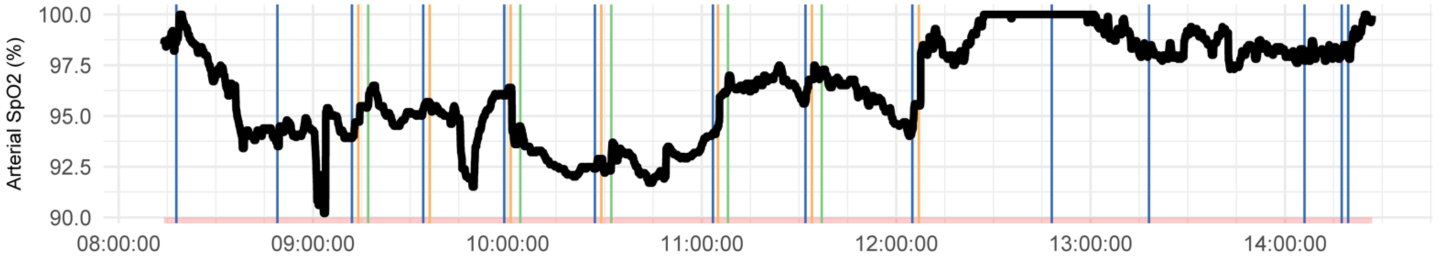

ID 13 – Session 3

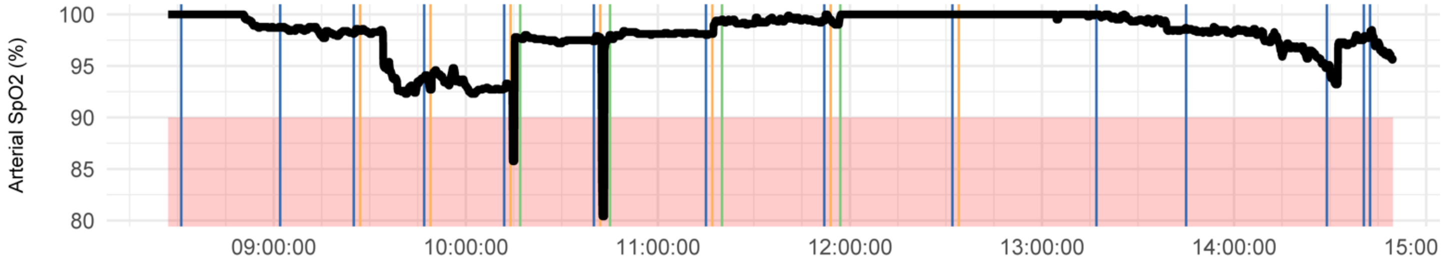

ID 15 – Session 1

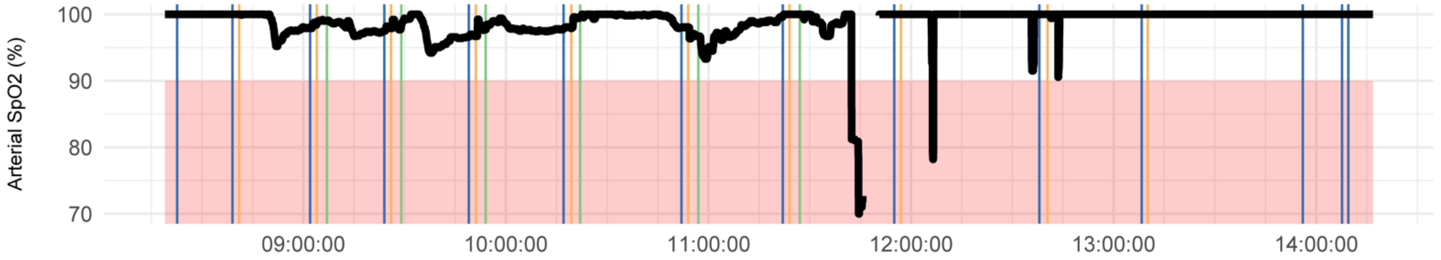

ID 15 – Session 2

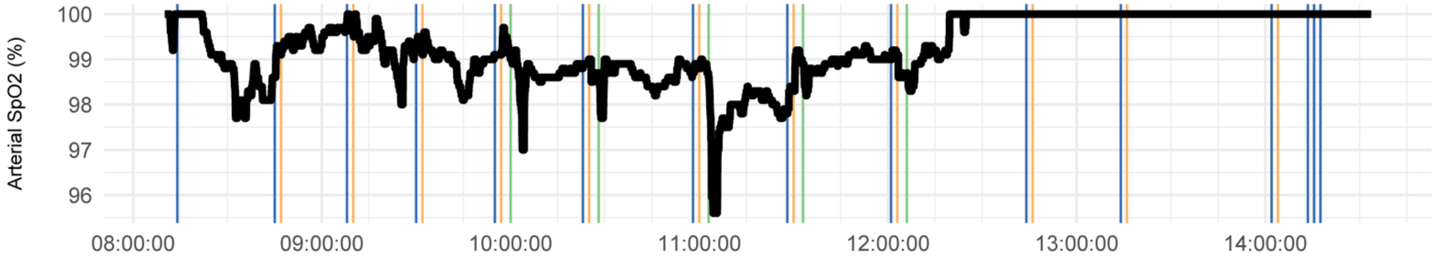

ID 15 – Session 3

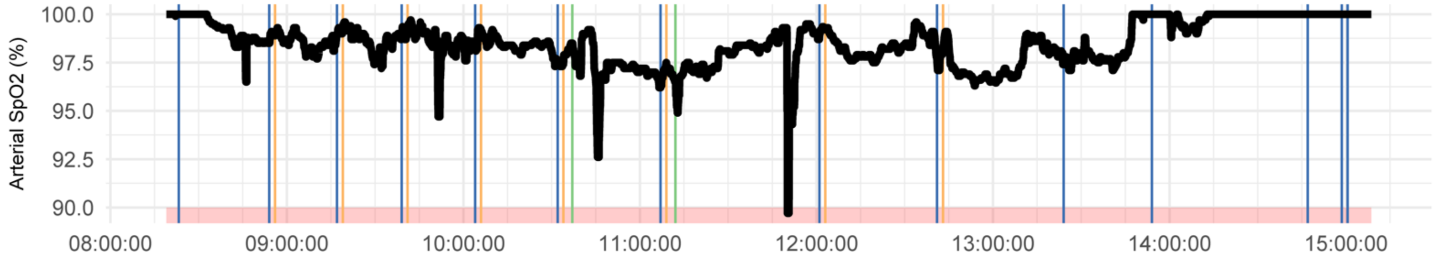

ID 17 – Session 1

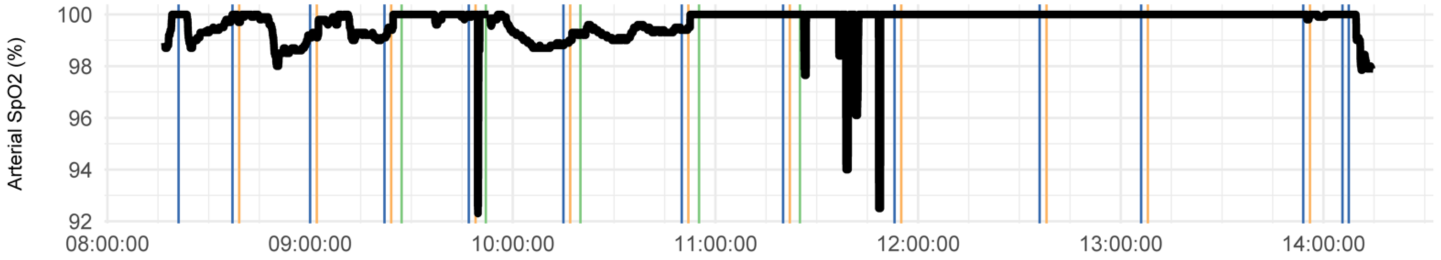

ID 17 – Session 2

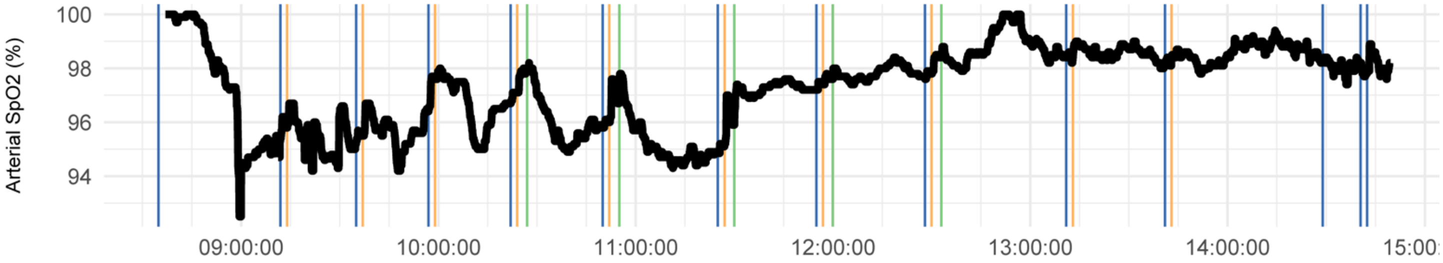

ID 17 – Session 3

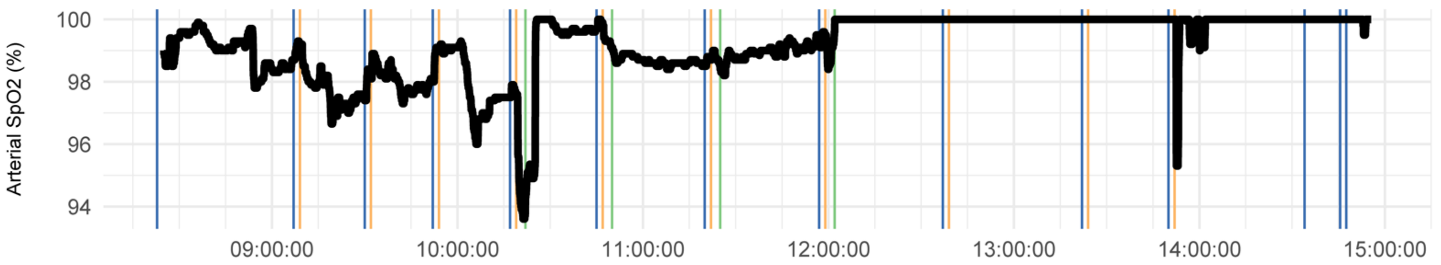

ID 18 – Session 1

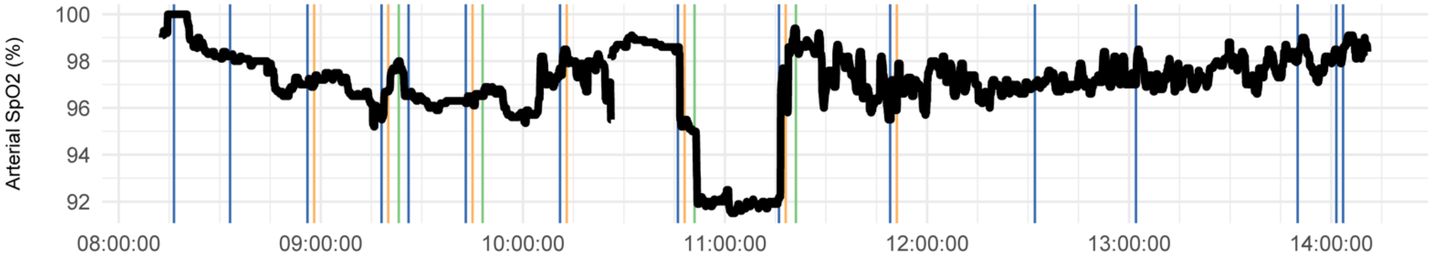

ID 18 – Session 2

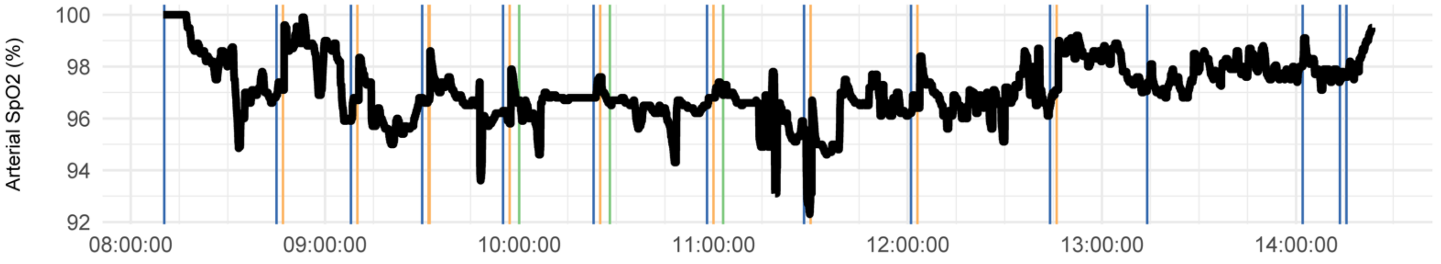

ID 18 – Session 3

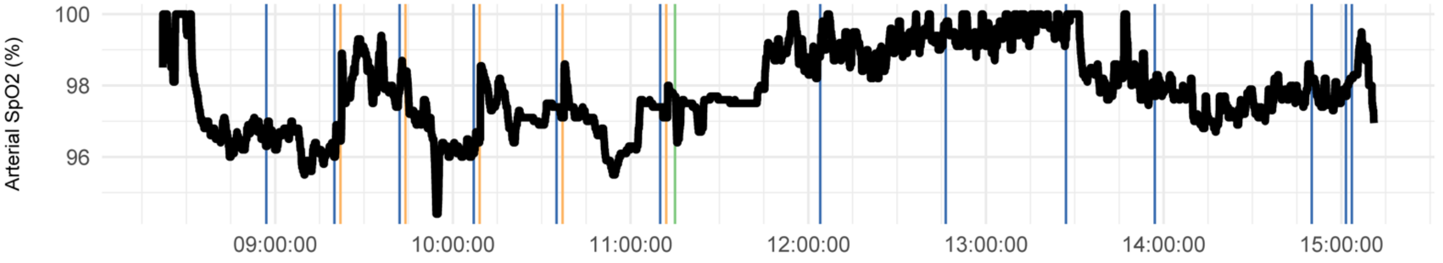

ID 19 – Session 1

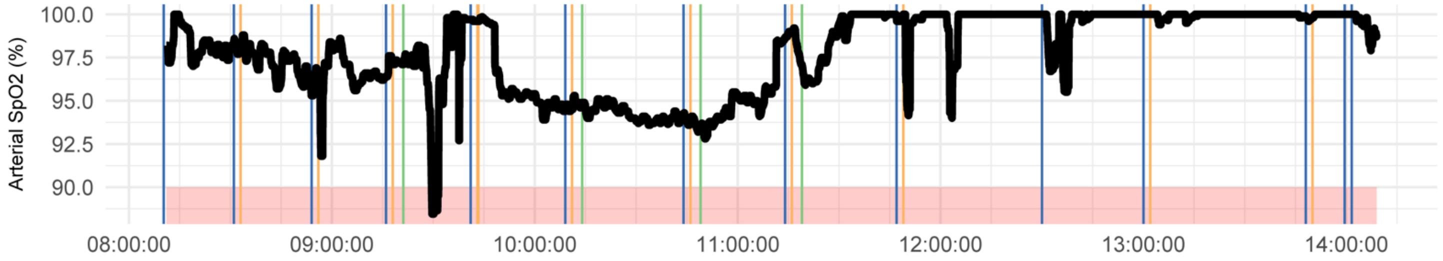

ID 19 – Session 2

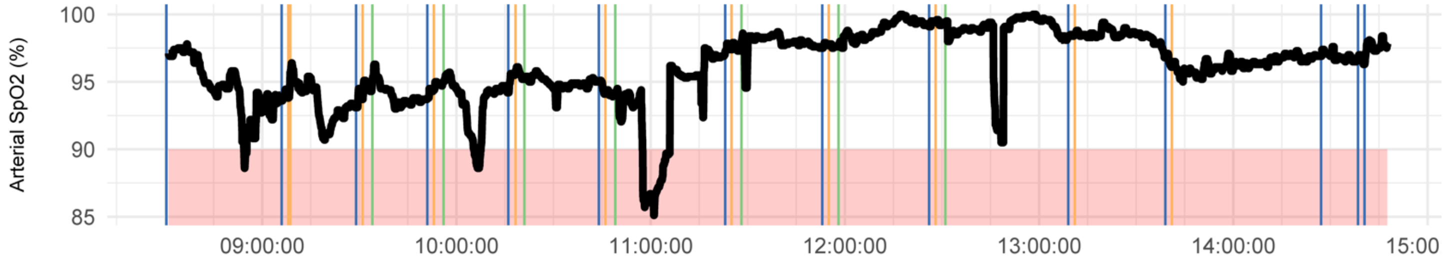

ID 19 – Session 3

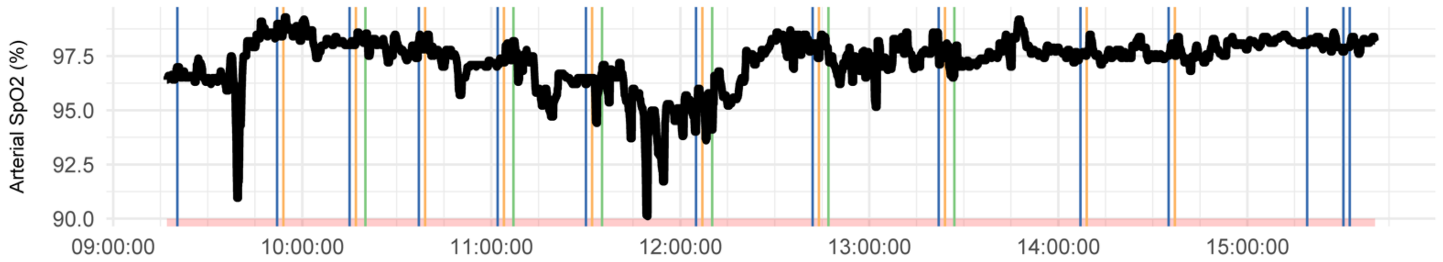

ID 21 – Session 1

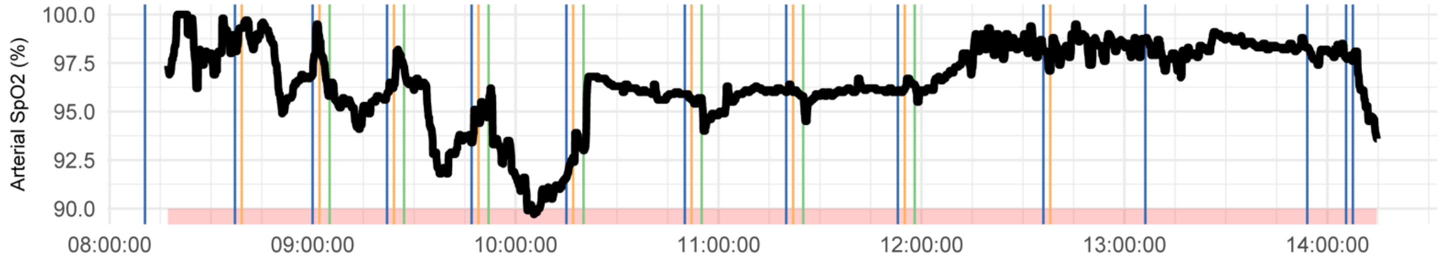

ID 21 – Session 2

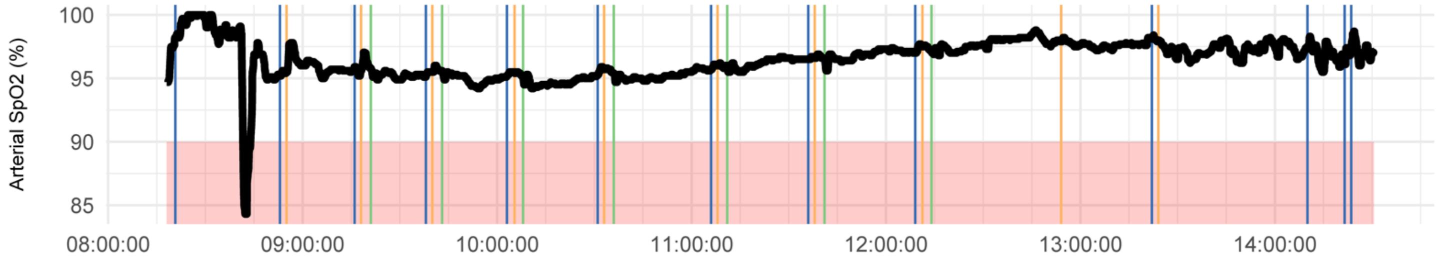

ID 21 – Session 3

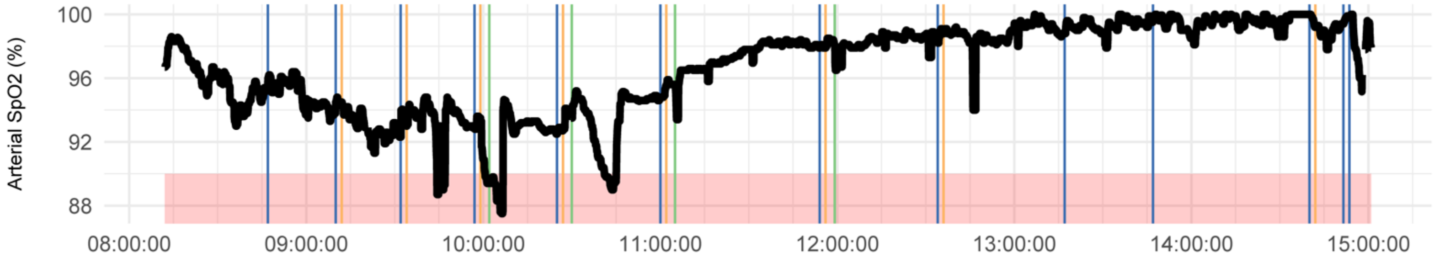

ID 22 – Session 1

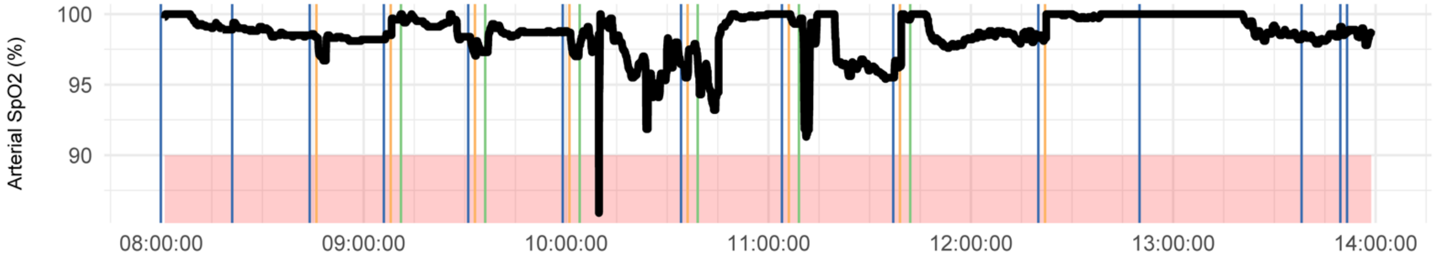

ID 22 – Session 2

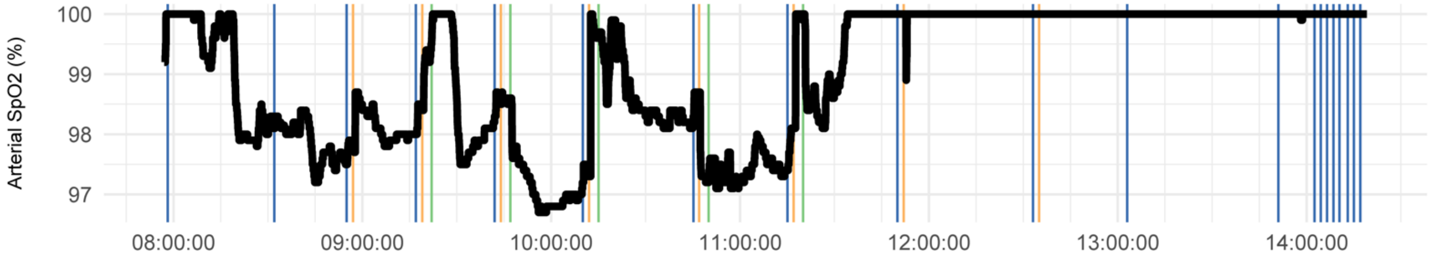

ID 22 – Session 3

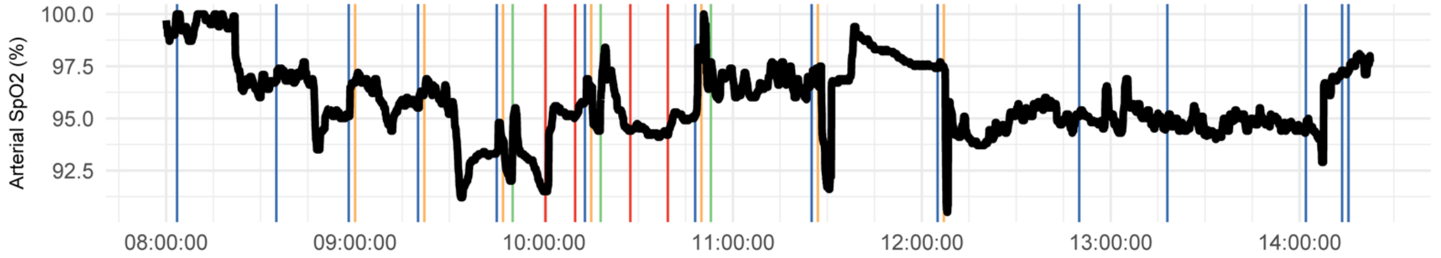

ID 23 – Session 1

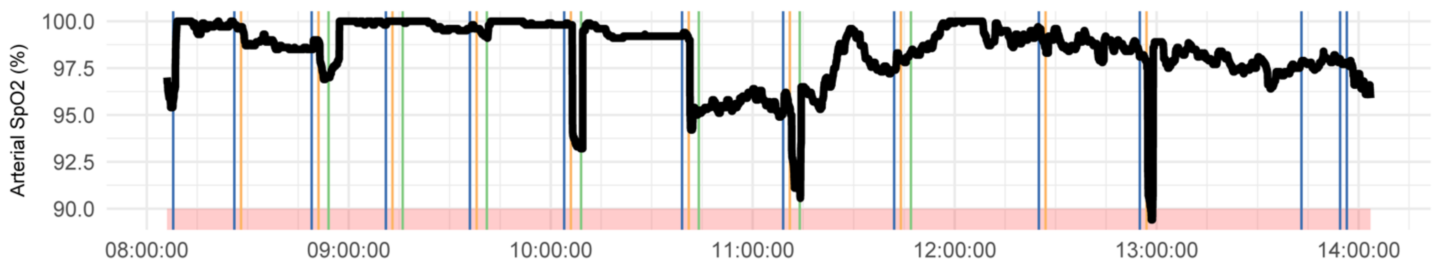

ID 23 – Session 2

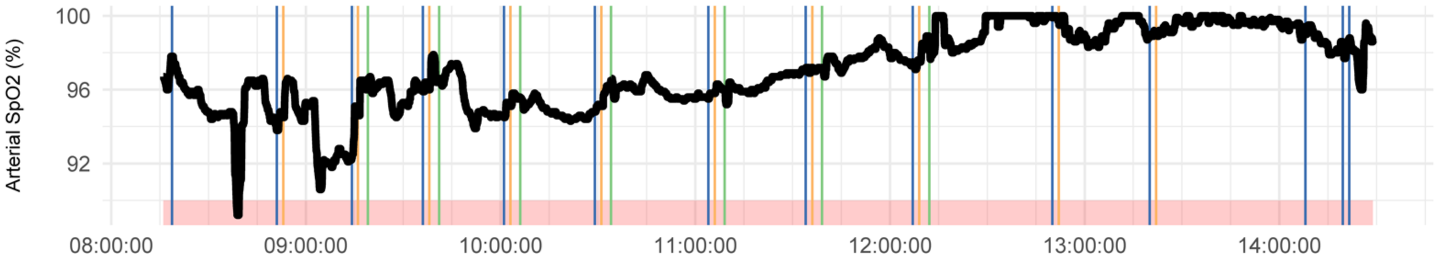

ID 23 – Session 3

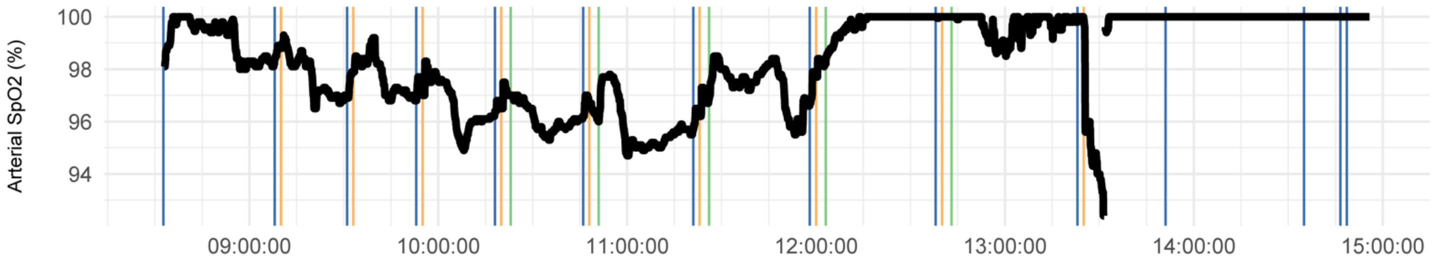

ID 24 – Session 1

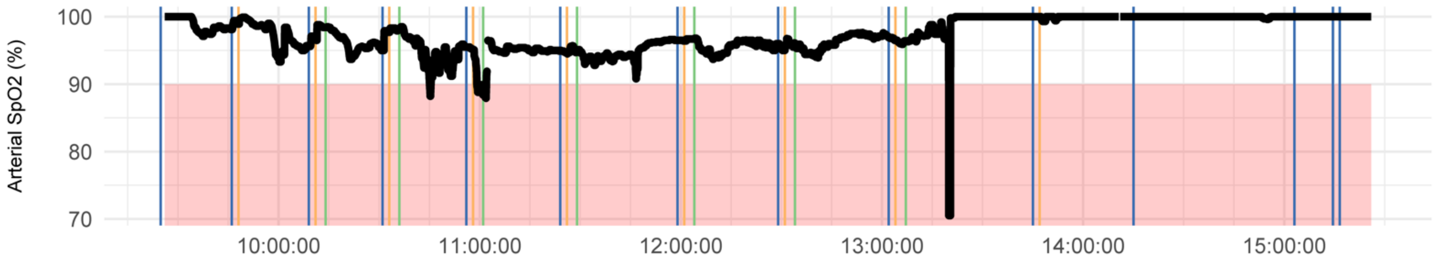

ID 24 – Session 2

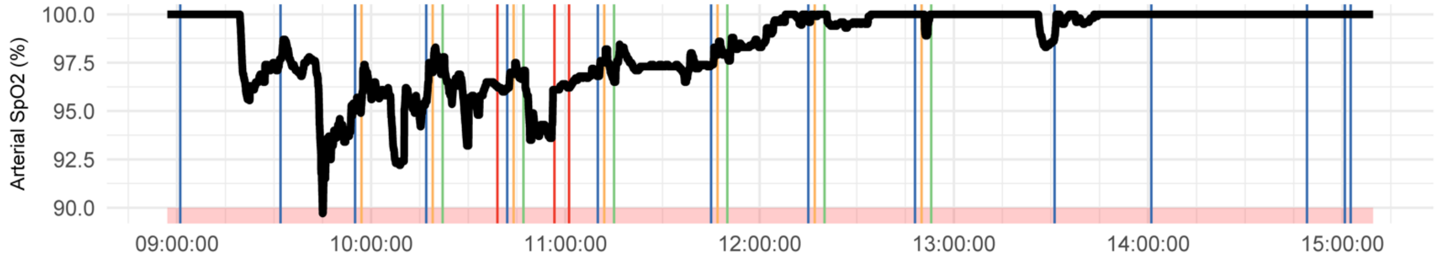

ID 24 – Session 3

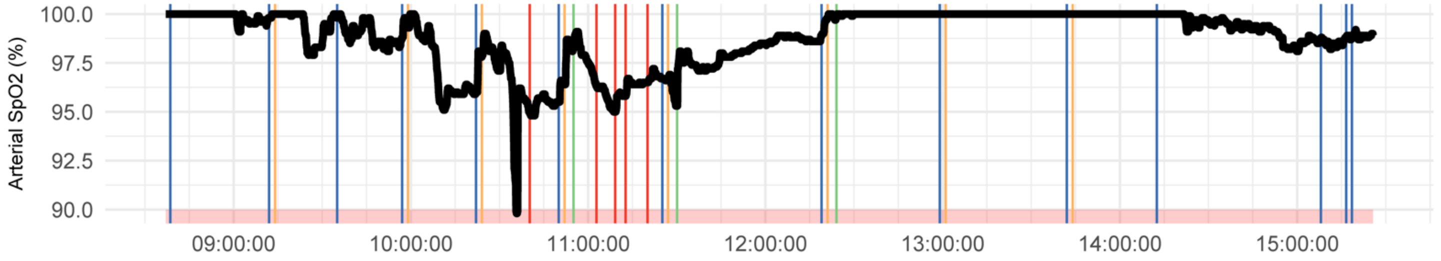

ID 111 – Session 1

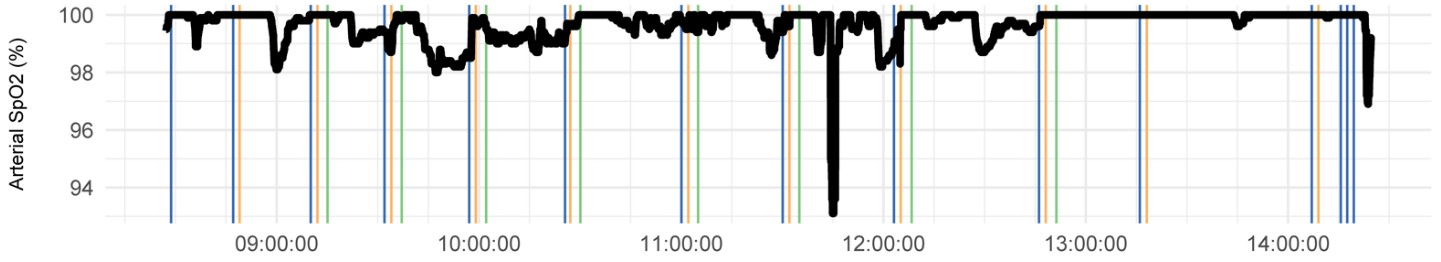

ID 111 – Session 2

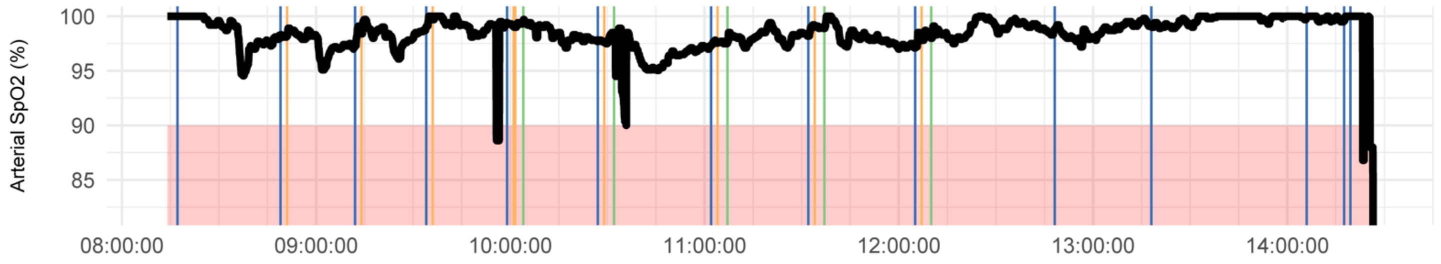

ID 111 – Session 3

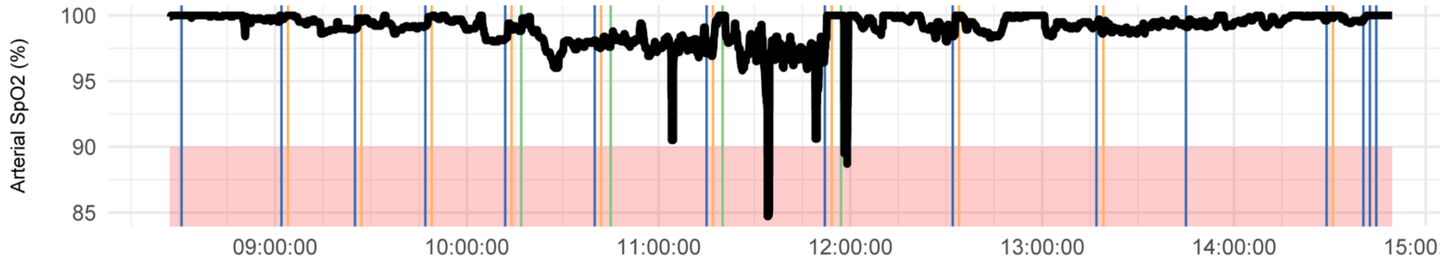

ID 114 – Session 1

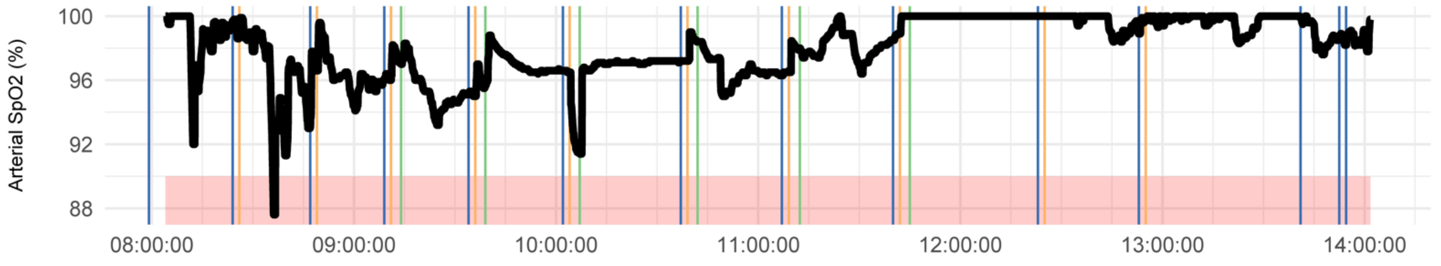

ID 114 – Session 2

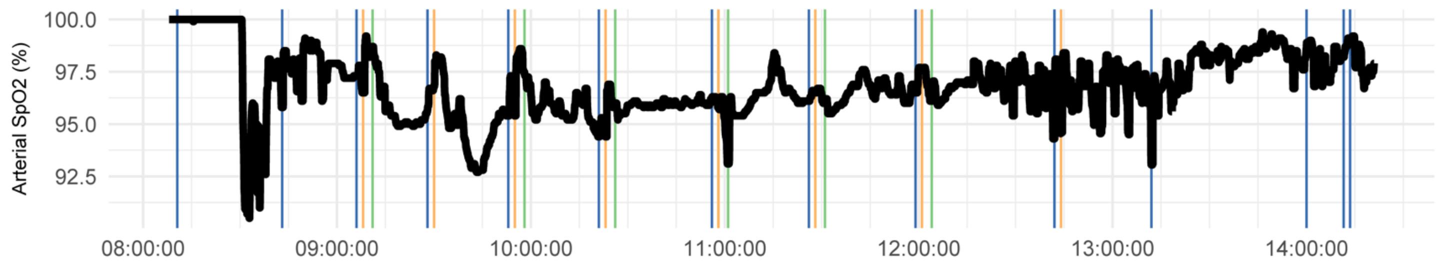

ID 114 – Session 3

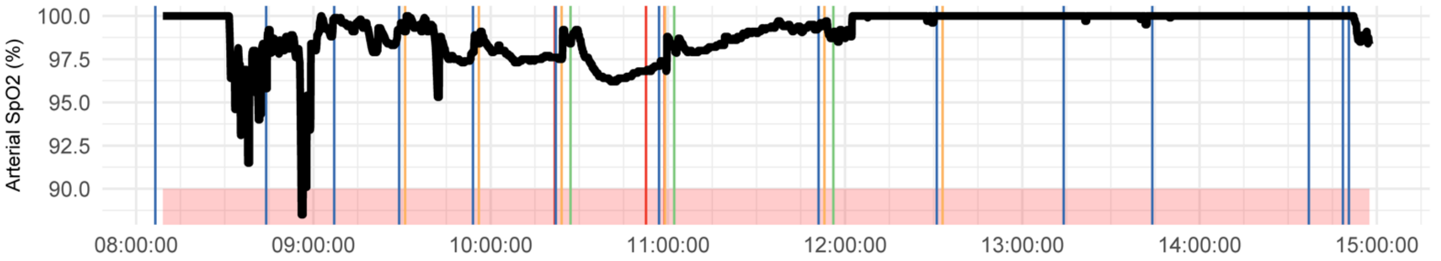

ID 116 – Session 1

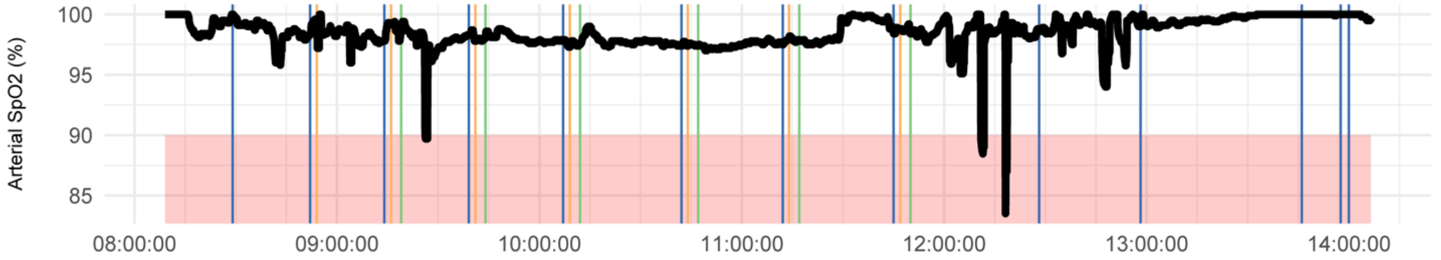

ID 116 – Session 2

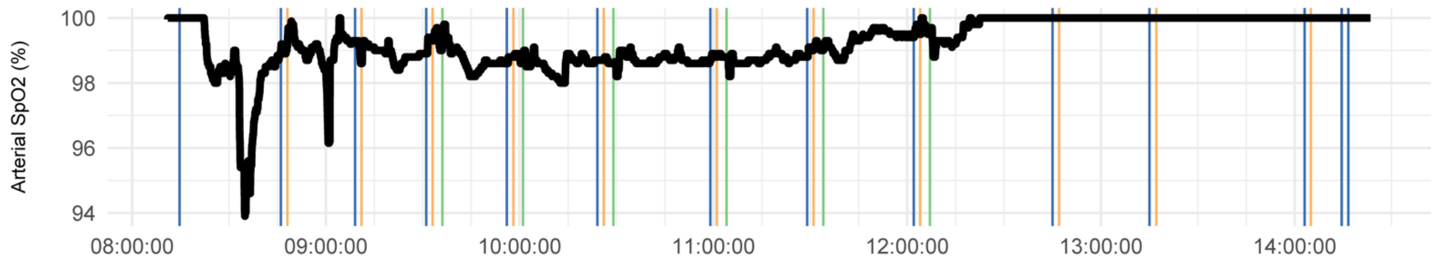

ID 116 – Session 3

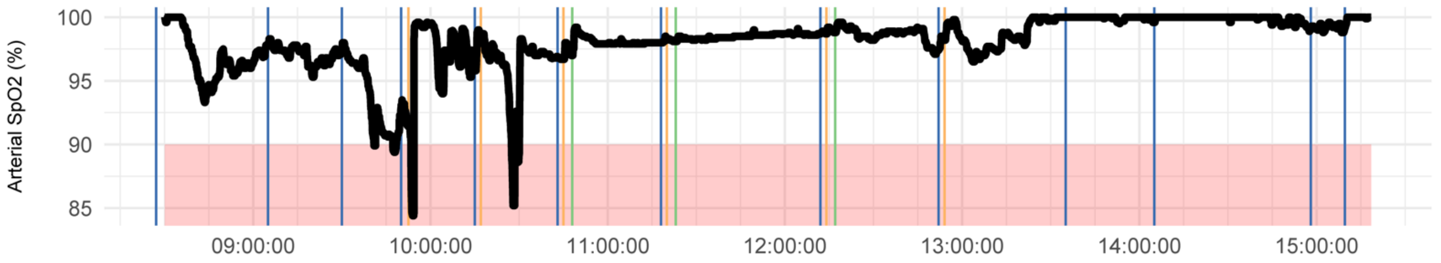

ID 120 – Session 1

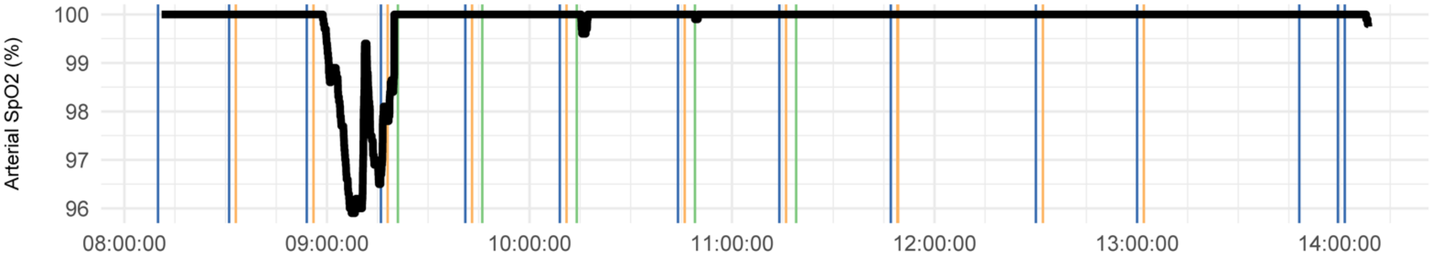

ID 120 – Session 2

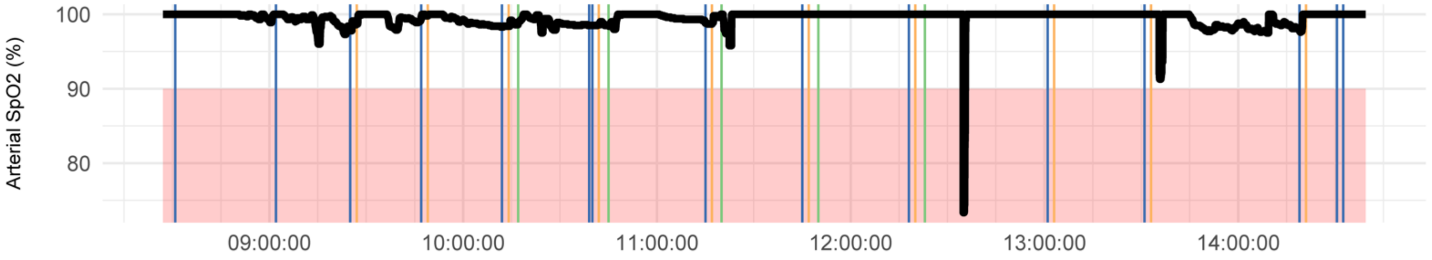

ID 120 – Session 3

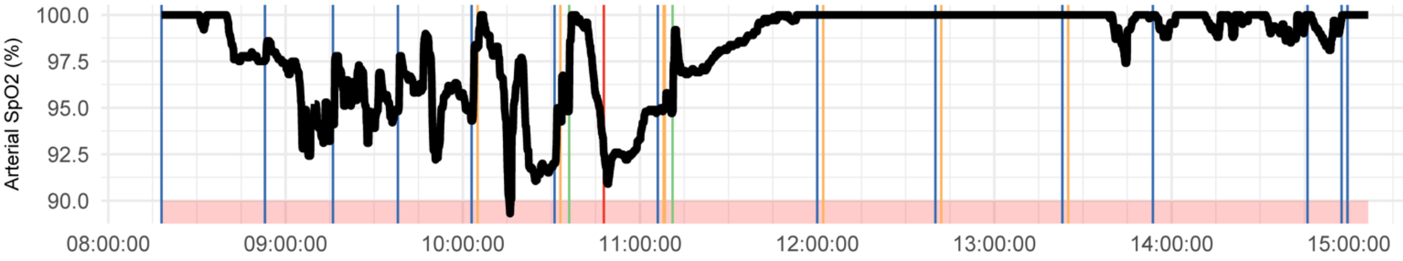

Supplement: Supplementary file 3 [file aln-142-666-s003.pdf]
