# Supplementary material for: Influence of Remifentanil on the Pharmacokinetics and Pharmacodynamics of Remimazolam in Healthy Volunteers
Source: Anesthesiology. 2025 Jan 15;142(4):666–79. doi: 10.1097/ALN.0000000000005348 (PMC11892992; doi:10.1097/ALN.0000000000005348)

Supplemental Digital Content 4

Figure 1. Comparison of measured remimazolam (panels A - D) and remifentanil concentrations (panels E & F) by TCI targets separated by session (2 vs. 3) and step-up versus step-down phase of the study. Measured concentrations for remimazolam and remifentanil are depicted by solid black circles. Measured CNS7054 concentrations are shown with solid grey circles. Geometric mean measured concentrations by TCI target are shown by horizontal black and grey solid lines, the TCI targets are denoted by horizontal orange lines. The black solid line is the line of unity.

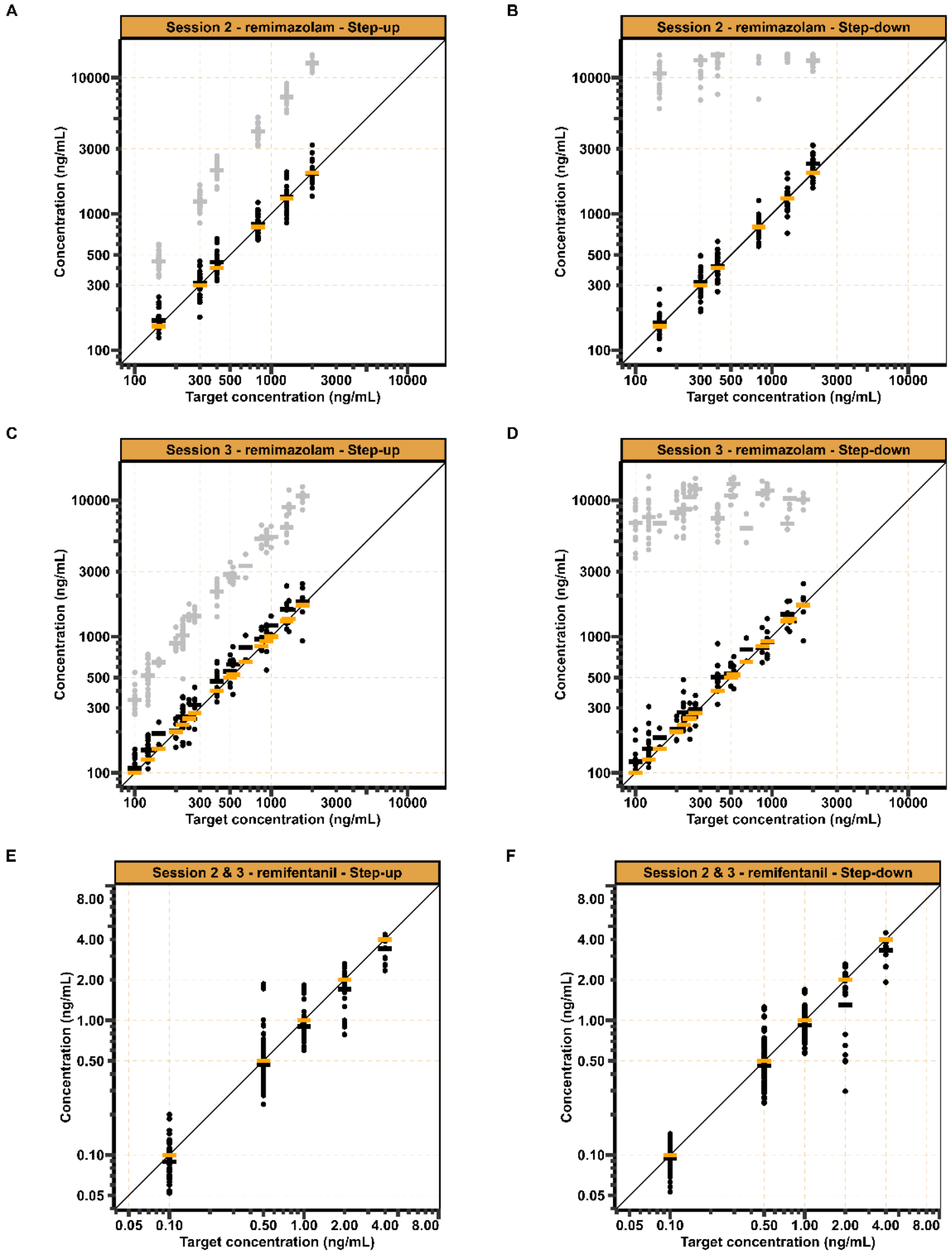

Supplement: Supplementary file 4 [file aln-142-666-s004.pdf]
