# Supplementary material for: Influence of Remifentanil on the Pharmacokinetics and Pharmacodynamics of Remimazolam in Healthy Volunteers
Source: Anesthesiology. 2025 Jan 15;142(4):666–79. doi: 10.1097/ALN.0000000000005348 (PMC11892992; doi:10.1097/ALN.0000000000005348)
Supplement: Supplementary file 5 [file aln-142-666-s005.pdf]

Supplemental Digital Content 5

<u>Development of the population pharmacokinetic model</u>

A total of 551, 549, and 552 plasma samples of remimazolam and CNS7054 collected in sessions 1, 2, and 3, respectively, were eligible for inclusion in the analyses. A total of 316 and 318 plasma samples of remifentanil collected in sessions 2 and 3 were eligible for inclusion in the analysis. A total of 9, 7, 4 remimazolam samples in session 1, 2, and 3, respectively, and 28, 26 and 33 CNS7054 samples in session 1, 2, and 3, respectively and 3 and 5 remifentanil samples in session 2, and 3, were below the lower limit of quantification. It was decided to remove these samples from the analysis for computational reasons and was expected to have minimal impact on the model development due to the relatively low number of samples below the lower limit of quantification for remimazolam, CNS7054 and remifentanil.

Non-linear mixed-effects modelling methodology was used to develop population pharmacokinetic models for remimazolam, CNS7054 and remifentanil. Model development was conducted using NONMEM version 7.5.0 (ICON Development Solutions, Ellicott City, MD, USA). Pre- and post-processing of the data was performed using R version 4.0.3 (R Core Team, 2020, Vienna, Austria) and Rstudio (RStudio Team, 2020, Boston, USA). The first order conditional estimation with interaction algorithm was used as implemented in NONMEM.

Model development initiated by re-fitting the joint remimazolam – CNS7054 population pharmacokinetic model that was developed using data of session 1 alone. This model has been reported previously and assumed that 80% of remimazolam was converted to CNS7054 (if incorrect, this will affect values of the model parameters of the metabolite) [1]. A prediction-corrected visual predictive check indicated reasonable performance (Figure 1). Comparison of model parameters between the model fitted to session 1 and the model fitted to all sessions indicated only slight differences in parameter estimates, except for the proportional error of the metabolite (104.3% increase in parameter estimate when estimated on all sessions, Table 1).

Remifentanil was administered using target-controlled infusion in sessions 2 and 3 targeting a single target concentration level per patient per session. It was *a priori* decided that the collected remifentanil plasma concentrations would not be informative enough to quantify a complex pharmacokinetic model. The model developed by Eleveld and colleagues was therefore used to quantify the pharmacokinetics of remifentanil in sessions 2 and 3. [2] The model adequately described the data with an MdAPE of 20.4% (minimum-maximum: 8.9% to 54.6%). A study effect in the residual error was implemented in the Eleveld remifentanil model, which made it difficult to assess which estimate to use for the residual error model for obtaining individual model predictions. [2] It was therefore decided to re-estimate the residual error using the available data while keeping all the structural model parameters fixed. This resulted in an additive error (Standard Deviation [SD]) of 0.03 ng/mL and a proportional error (Coefficient of Variation [CV%]) of 23.4%, which were considered plausible estimates.

The influence of remifentanil on the pharmacokinetics of remimazolam and CNS7054 was then formally tested using a sigmoid equation on model parameters for remimazolam clearance and apparent CNS7054 clearance. No influence was detected of remifentanil on the clearance of remimazolam (Δ Objective Function Value [OFV]: 0.0 points), but a clear influence of remifentanil was detected on the apparent clearance of CNS7054 (ΔOFV: -315.4). After inclusion of the influence of remifentanil on apparent CNS7054 clearance, the influence of remifentanil on remimazolam clearance was re-evaluated. An improvement was observed in the overall model fit (ΔOFV: -68.1 points), but this also resulted in a model with a very large condition number (8555.7 versus 156.5) indicating that this model was very unstable. The model was therefore reduced by removing the influence of remifentanil on remimazolam clearance.

The final model structure (Figure 2), parameter estimates (Table 2), goodness-of-fit plots (Figure 3a-c, 4a-c and 5a-b), prediction-corrected visual predictive checks (Figure 6), log-likelihood profiles (Figure 7 and 8) and model code are provided below. In general, model parameters were estimated with accurate precision. Relatively high inter-individual variability is estimated for Q3 and ktr. Shrinkage

values were low and the condition number (156.5) is well below 1000. Some structural deviation from the population mean can be observed in the CWRES versus Time plot of session 3 for remimazolam. This trend was however not as clear in the prediction-corrected visual predictive check plots. All in all, it can be concluded that the model is able to describe the observed remimazolam, CNS7054 and remifentanil concentrations.

References

[1] A remimazolam dose finding study in healthy volunteers using target controlled infusion. Vellinga R, Koomen JV, Eleveld DJ, Stöhr T, Struys MMRF, Colin PJ. Anesthesiology [Submitted]

[2] An Allometric Model of Remifentanil Pharmacokinetics and Pharmacodynamics. Eleveld DJ, Proost JH, Vereecke H, Absalom AR, Olofsen E, Vuyk J, Struys MMRF. Anesthesiology. 2017 Jun;126(6):1005-1018.

Figure 1. Prediction-corrected Visual Predictive Checks for Remimazolam and CNS7054 (metabolite) stratified for session on linear scale. *Red solid line represents the median observations, blue dashed lines represent the 5th and 95th percentiles of the observations. Red shaded area represents the 95% confidence interval of the median prediction. Blue shaded areas are 95% confidence intervals of the model predictions of the 5th and 95th percentiles.*

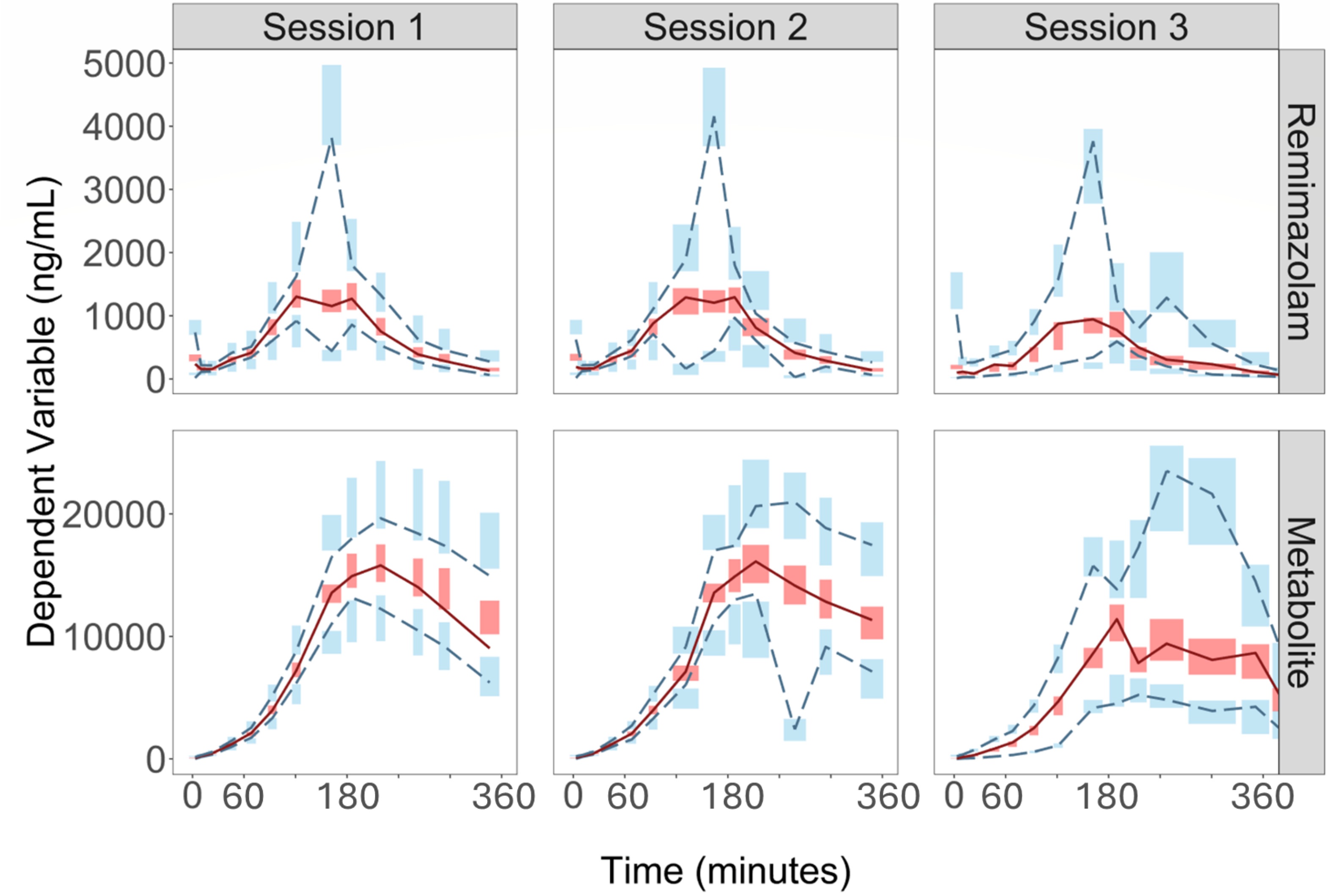

Table 1. Comparison model parameters

Parameter	Model (session 1)	Model (All)	Model (session 1) versus Model (All)	Model (All + interaction)	Model (All) versus Model (All + interaction)
Remimazolam					
Volume of distribution of central compartment - parent (L.70 kg^{-1})	5.36	5.54	3.3%	5.31	-4.0%
Volume of distribution of peripheral 1 compartment - parent (L.70 kg^{-1})	11.67	12.16	4.2%	12.81	5.3%
Volume of distribution of peripheral 2 compartment - parent (L.70 kg^{-1})	32.73	41.67	27.3%	40.04	-3.9%
Clearance of central compartment - parent (L.min-1.70 kg^{-1})	1.25	1.17	-6.6%	1.15	-1.4%
Intercompartmental clearance between compartment 1 & 2 - parent (L.min^{-1}.70 kg^{-1})	0.74	0.90	22.5%	0.91	0.9%
Intercompartmental clearance between compartment 2 & 3 (L.min^{-1}.70 kg^{-1})	0.39	0.30	-22.4%	0.29	-3.2%
Proportional residual error - parent (*100% CV)	0.27	0.27	0.3%	0.24	-10.7%
CNS7054					
Apparent clearance of central compartment - metabolite (L.min^{-1}.70 kg^{-1})	0.05	0.04	-18.9%	0.05	16.2%
Apparent volume of distribution of central compartment - metabolite (L.70 kg^{-1})	7.32	7.90	8.0%	7.77	-1.7%
Transit rate constant (min^{-1}.70 kg^{-1})	0.21	0.28	31.3%	0.27	-2.9%
Fraction metabolised	0.80	0.80	0.0%	0.80	0.0%
Proportional residual error - metabolite (*100% CV)	0.06	0.13	104.3%	0.12	-11.0%
Additive residual error (SD)	23.70	22.94	-3.2%	25.28	10.2%
Interaction CNS7054-Remifentanil					
Inhibitory concentration 50 (ng/mL)				8.00	
Hill factor				0.61	
Between-subject variability					
Clearance of central compartment - parent (L.min-1.70 kg^{-1})	0.04	0.04	7.2%	0.04	2.3%
Intercompartmental clearance between compartment 2 & 3 (L.min^{-1}.70 kg^{-1})	0.52	0.28	-46.5%	0.29	1.9%
Clearance of central compartment - metabolite (L.min^{-1}.70 kg^{-1})	0.08	0.08	4.0%	0.09	4.0%
Transit rate constant (min^{-1}.70 kg^{-1})	0.49	0.33	-32.9%	0.37	13.8%

Figure 2. Model structure and equations for rate constants

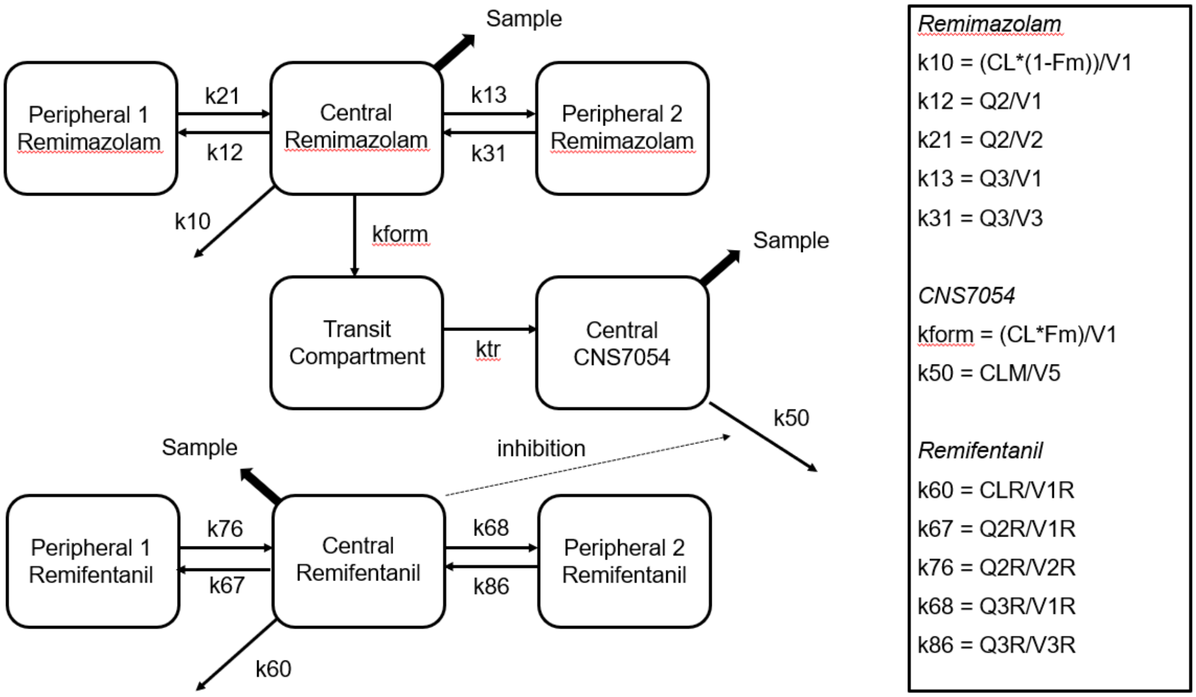

Table 2. Parameter estimates

Parameter Name	Parameter	Estimates	LL (95% CI)	UL (95% CI)	IIV (%)	LL (95% CI)	UL (95% CI)	Shrinkage (%)
Remimazolam								
Volume of distribution of central compartment - parent (L.70 kg^{-1})	V1	5.3	4.9	5.8				
Volume of distribution of peripheral 1 compartment - parent (L.70 kg^{-1})	V2	12.8	11.3	14.6				
Volume of distribution of peripheral 2 compartment - parent (L.70 kg^{-1})	V3	40.0	36.6	43.7				
Clearance of central compartment - parent (L.min-1.70 kg^{-1})	CL	1.15	1.06	1.25	19.8	14.9	27.5	<0.01
Intercompartmental clearance between compartment 1 & 2 - parent (L.min^{-1}.70 kg^{-1})	Q2	0.91	0.84	0.99				
Intercompartmental clearance between compartment 2 & 3 (L.min^{-1}.70 kg^{-1})	Q3	0.29	0.23	0.37	57.5	42.2	86.7	0.4
Proportional residual error - parent (% CV)		24.2	23.2	25.0				
CNS7054								
Apparent clearance of central compartment - metabolite (L.min^{-1}.70 kg^{-1})	CLM	0.05	0.04	0.05	30.3	22.8	42.5	<0.01
Apparent volume of distribution of central compartment - metabolite (L.70 kg^{-1})	V5	7.8	7.6	7.9				
Transit rate constant (min^{-1}.70 kg^{-1})	KTR	0.3	0.2	0.4	67.0	46.9	106.0	3.4
Fraction metabolized*	FM	0.8 (fixed)						
Proportional residual error - metabolite (% CV)		11.6	11.2	12.1				
Additive residual error (SD)		25.3	22.0	28.9				
Interaction CNS7054-Remifentanil								
Inhibitory concentration 50 (ng/mL)	IC50	8.0	5.5	13.5				
Hill factor	GAM	0.6	0.5	0.7				

*The inter-individual variability (IIV) was expressed as coefficient of variation (CV) and calculated as $\sqrt{(e^{IIV} - 1)}$*100%. The residual variability was expressed as standard deviation (SD) or CV for the additive and proportional residual error terms, respectively. The CV for the proportional residual error was calculated as √(residual error)*100%. The lower limit (LL) and upper limit (UL) of the 95% confidence intervals (CI) of the parameters were derived using log-likelihood profiling. Eps shrinkage was 2.5%.*

** The fraction metabolized was fixed in the model based on the information provided in the European Public Assessment Report for Byfavo stating that 80% of remimazolam is metabolised to CNS7054 (European Public Assessment Report - Byfavo (EMA/160756/2021))*

Figure 3a. Goodness-of-fit plots – remimazolam in session 1. *Observations are expressed as points (•), blue dotted line is a locally estimated scatterplot (LOESS) smoother.*

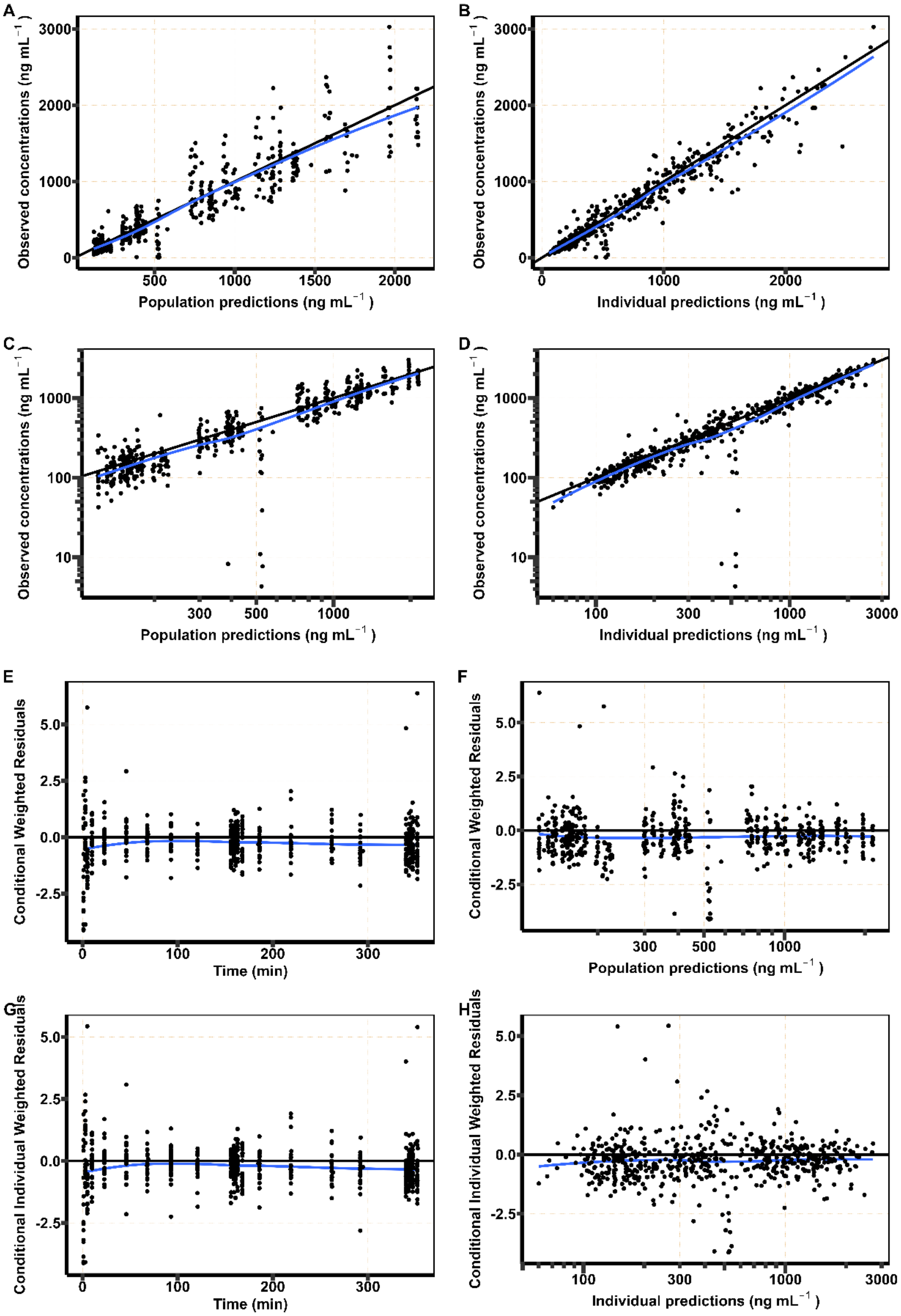

Figure 3b. Goodness-of-fit plots – remimazolam in session 2. *Observations are expressed as points (•), blue dotted line is a LOESS smoother.*

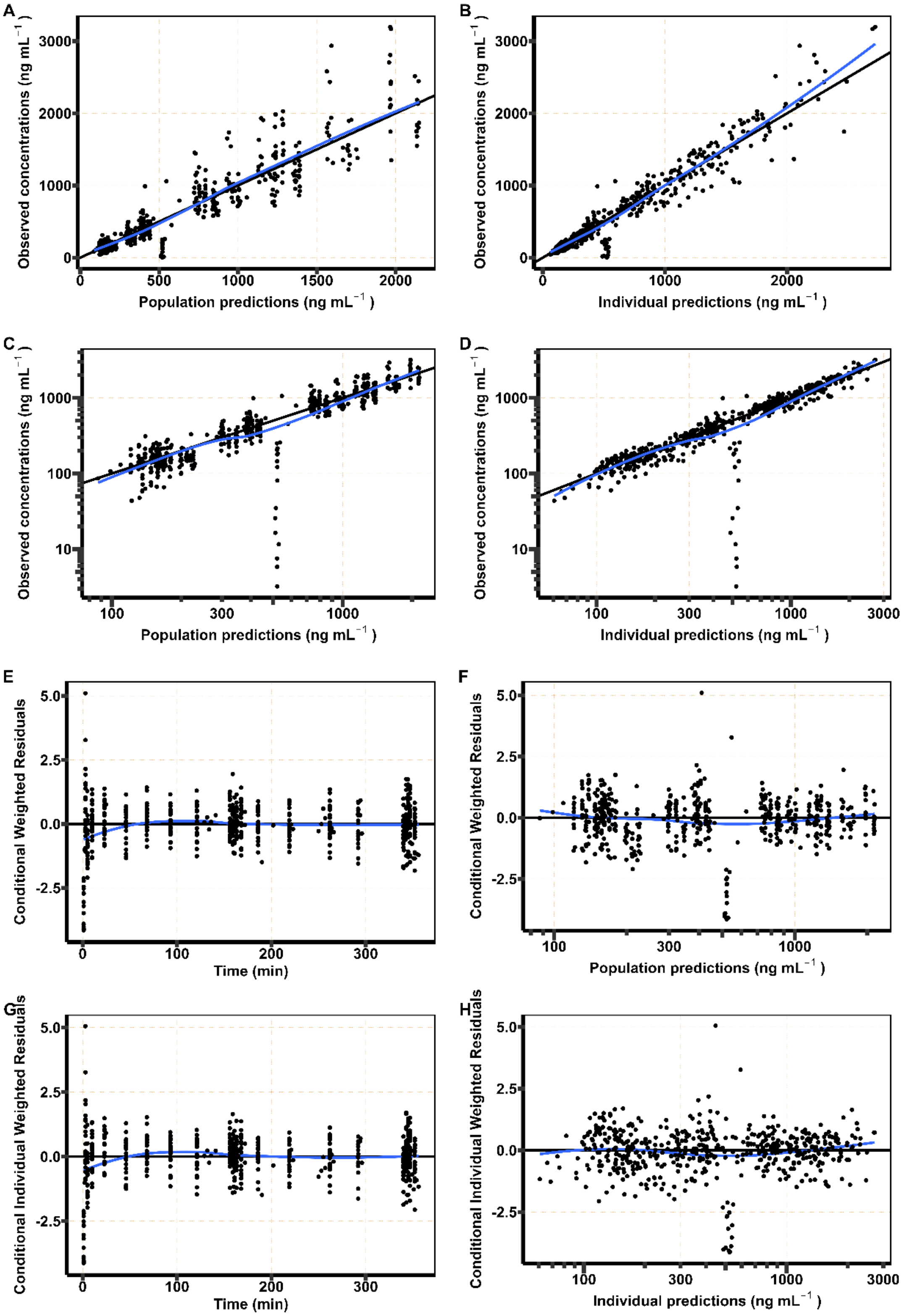

Figure 3c. Goodness-of-fit plots – remimazolam in session 3. *Observations are expressed as points (•), blue dotted line is a LOESS smoother.*

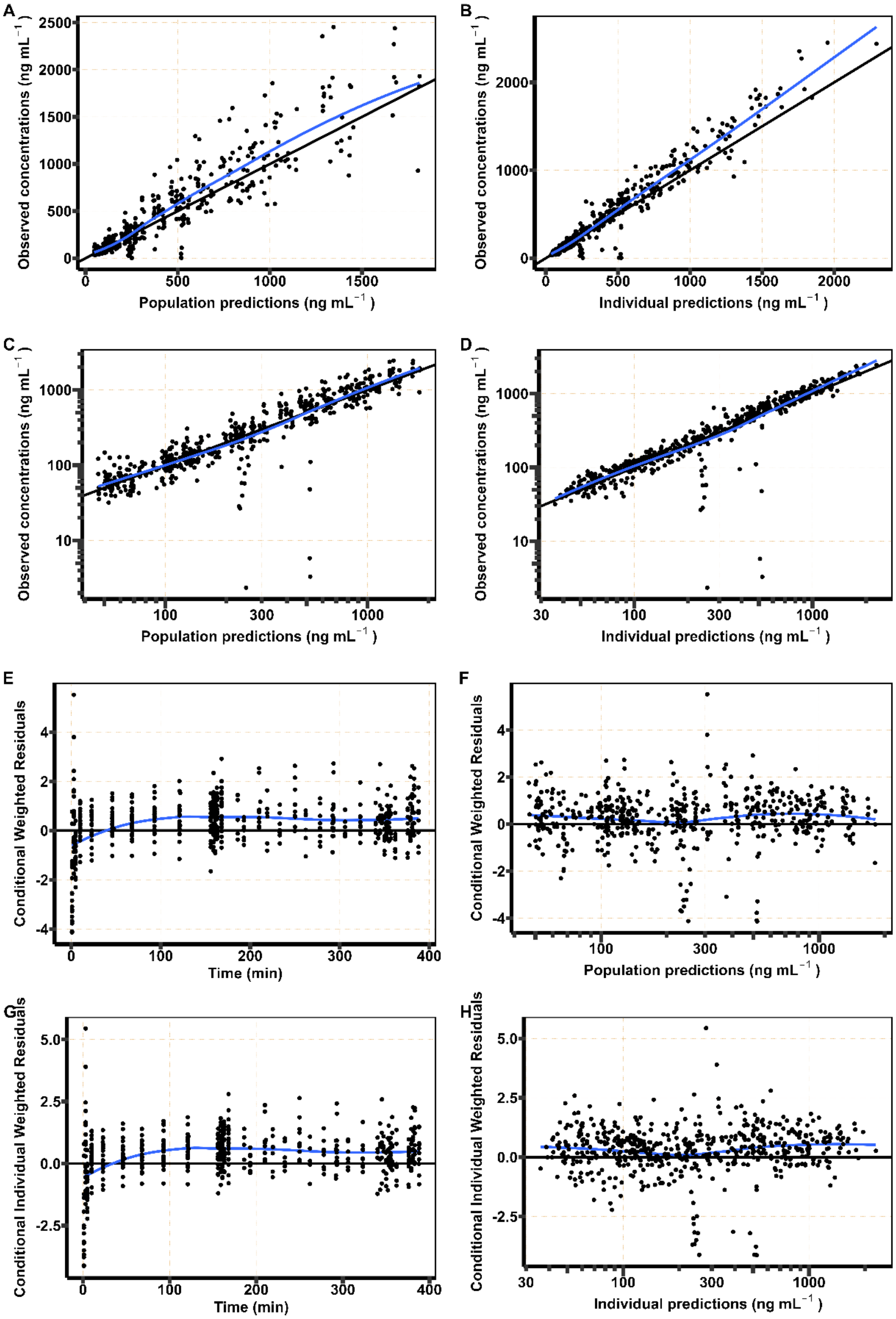

Figure 4a. Goodness-of-fit plots – CNS7054 in session 1. *Observations are expressed as points (•), blue dotted line is a LOESS smoother.*

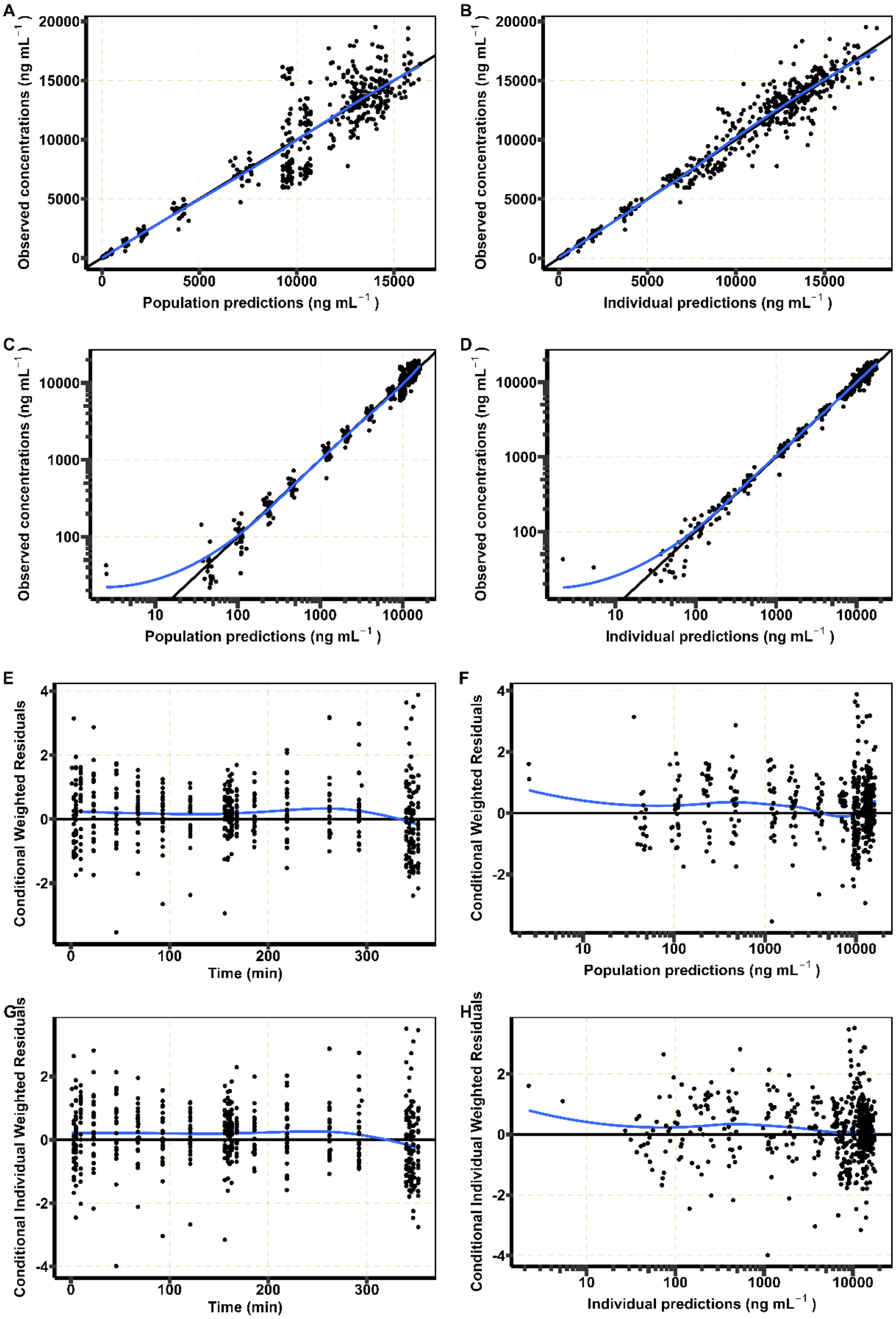

Figure 4b. Goodness-of-fit plots – CNS7054 in session 2. *Observations are expressed as points (•), blue dotted line is a LOESS smoother.*

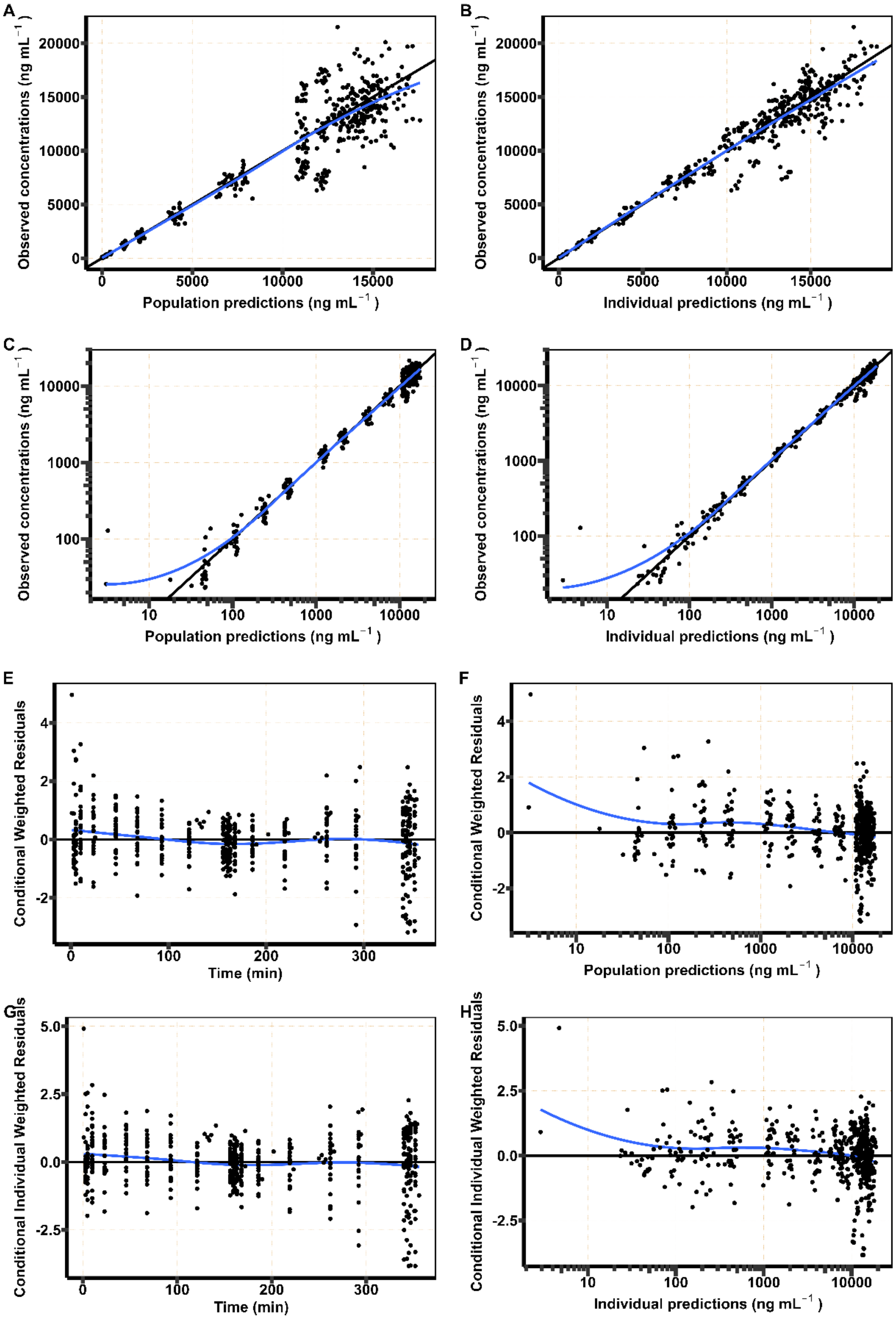

Figure 4c. Goodness-of-fit plots – CNS7054 in session 3. *Observations are expressed as points (•), blue dotted line is a LOESS smoother.*

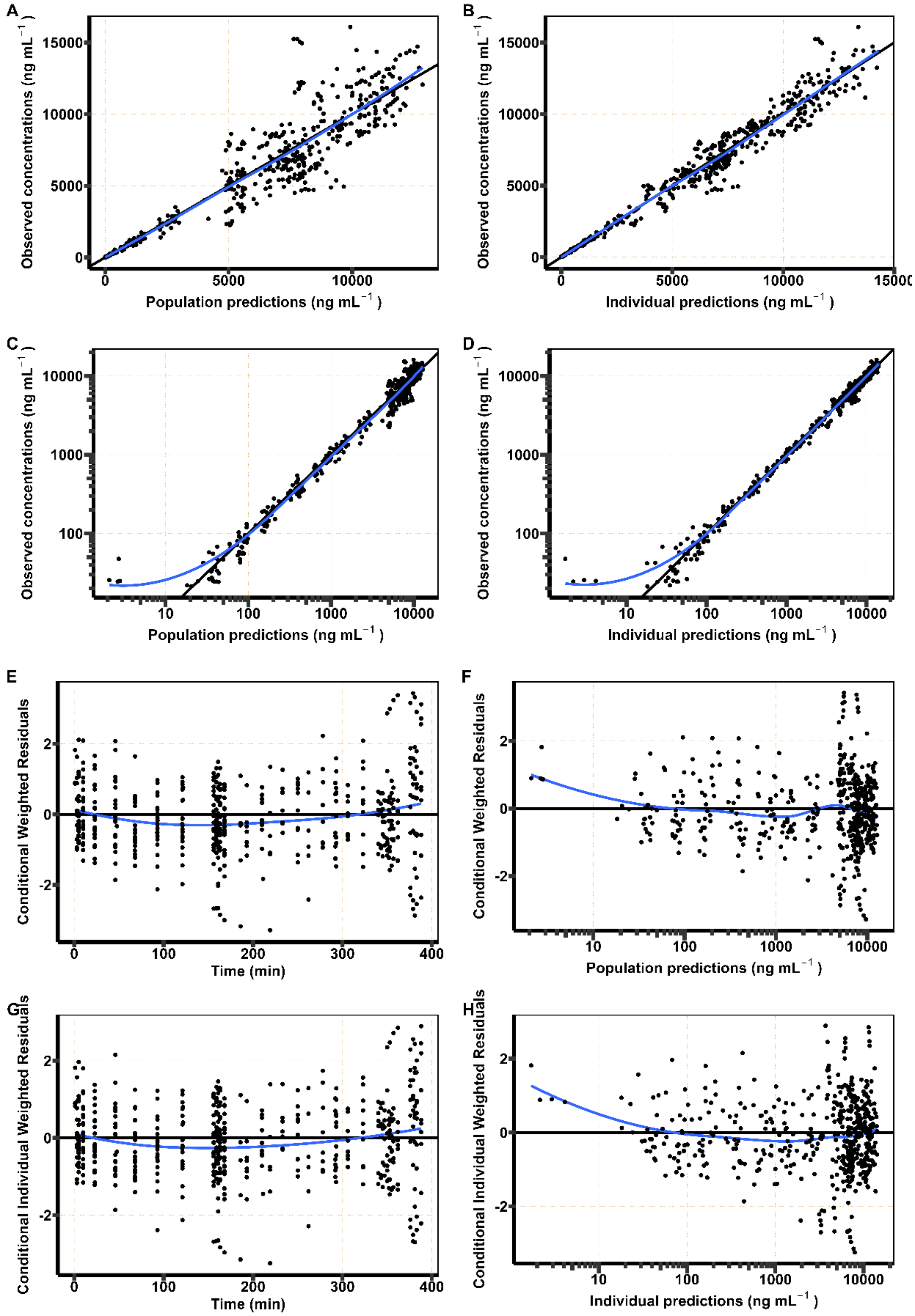

Figure 5a. Goodness-of-fit plots – Remifentanil in session 2. *Observations are expressed as points (•), blue dotted line is a LOESS smoother.*

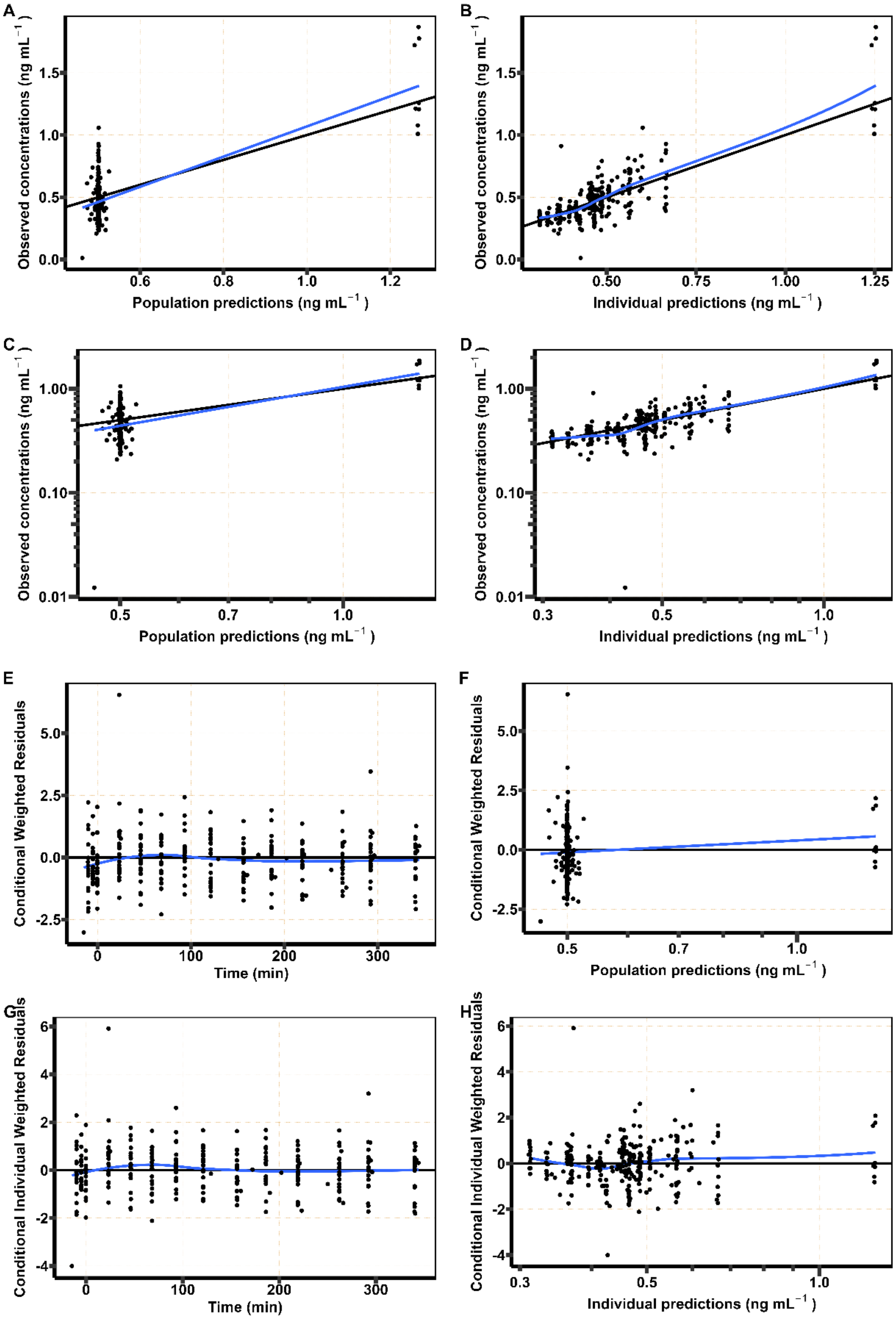

Figure 5b. Goodness-of-fit plots – Remifentanil in session 3. *Observations are expressed as points (•), blue dotted line is a LOESS smoother.*

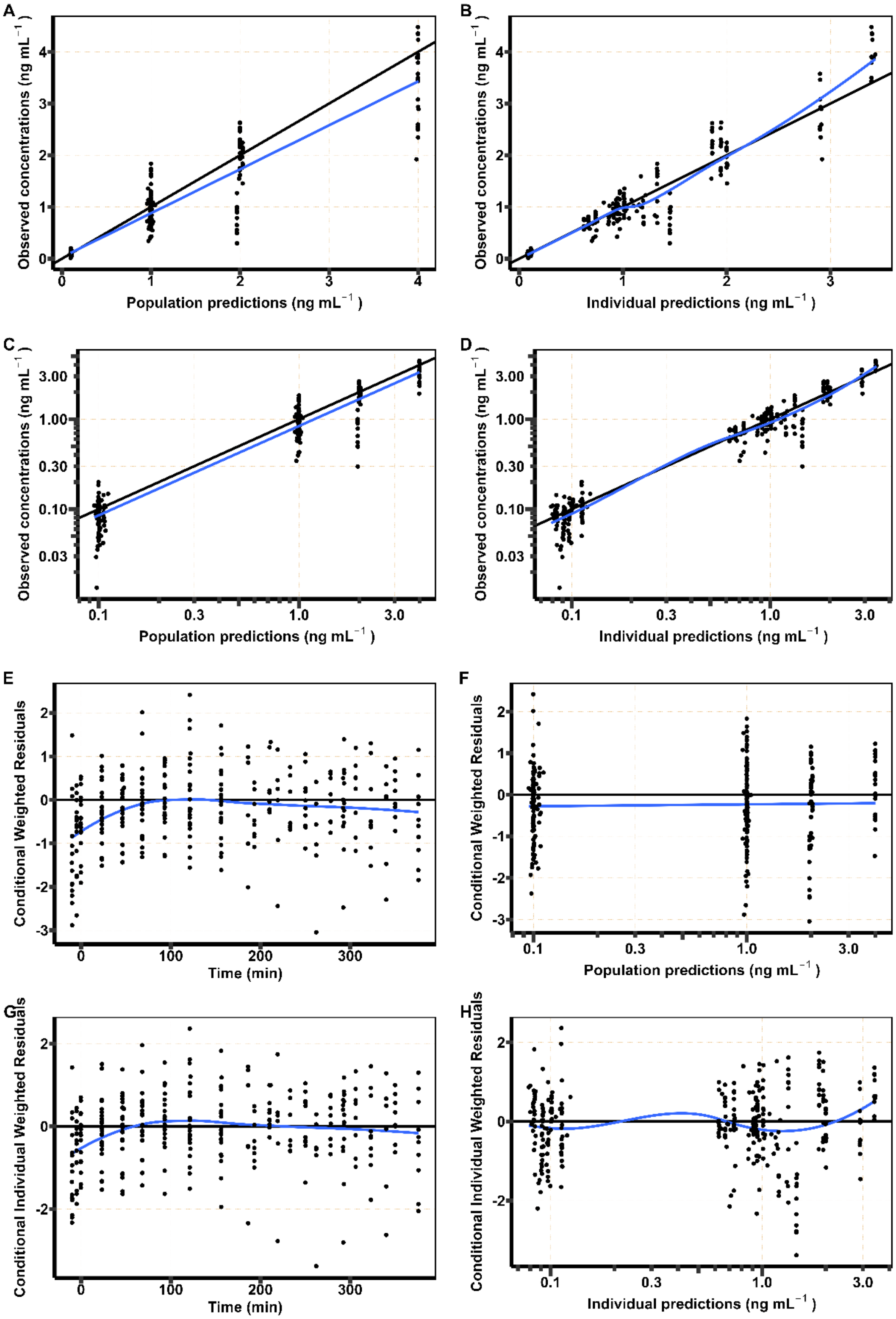

Figure 6a. Prediction-corrected Visual Predictive Checks for Remimazolam and CNS7054 (metabolite) stratified for session on linear scale. *Red solid line represents the median observations, blue dashed lines represent the 5th and 95th percentiles of the observations. Red shaded area represents the 95% confidence interval of the median prediction. Blue shaded areas are 95% confidence intervals of the model predictions of the 5th and 95th percentiles.*

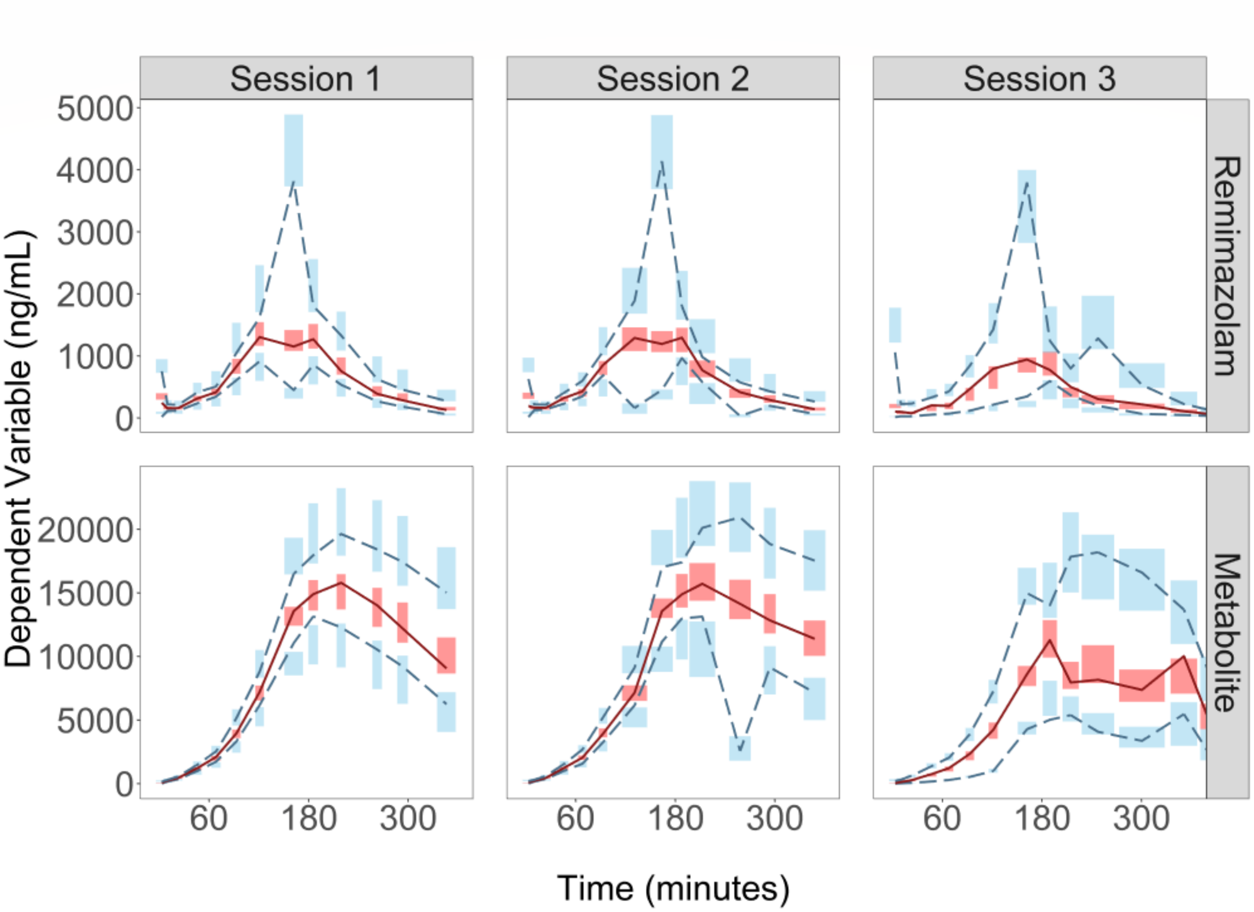

Figure 6b. Prediction-corrected Visual Predictive Checks for Remifentanil stratified for session on log-linear scale. *Black solid line represents the median observations, black dashed lines represent the 5th and 95th percentiles of the observations. Red shaded area represents the 95% confidence interval of the median prediction. Blue shaded areas are 95% confidence intervals of the model predictions of the 5th and 95th percentiles.*

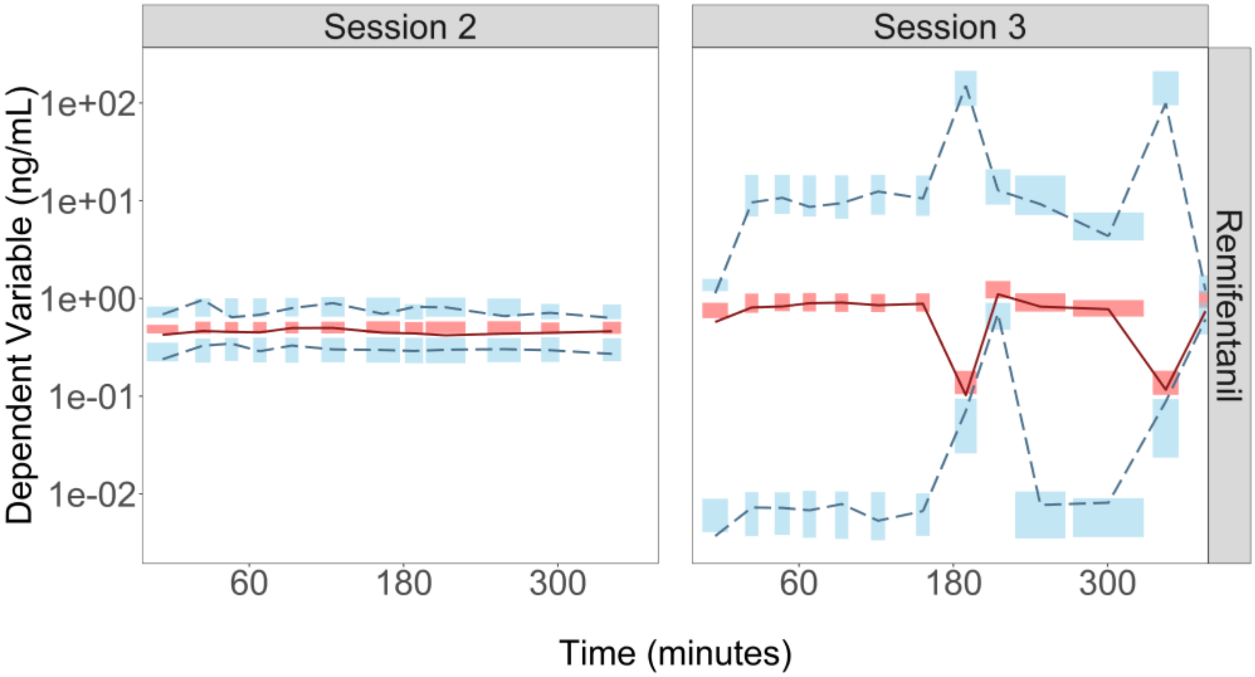

Figure 7. Log-likelihood profiles – THETAs and SIGMAs – pharmacokinetic model

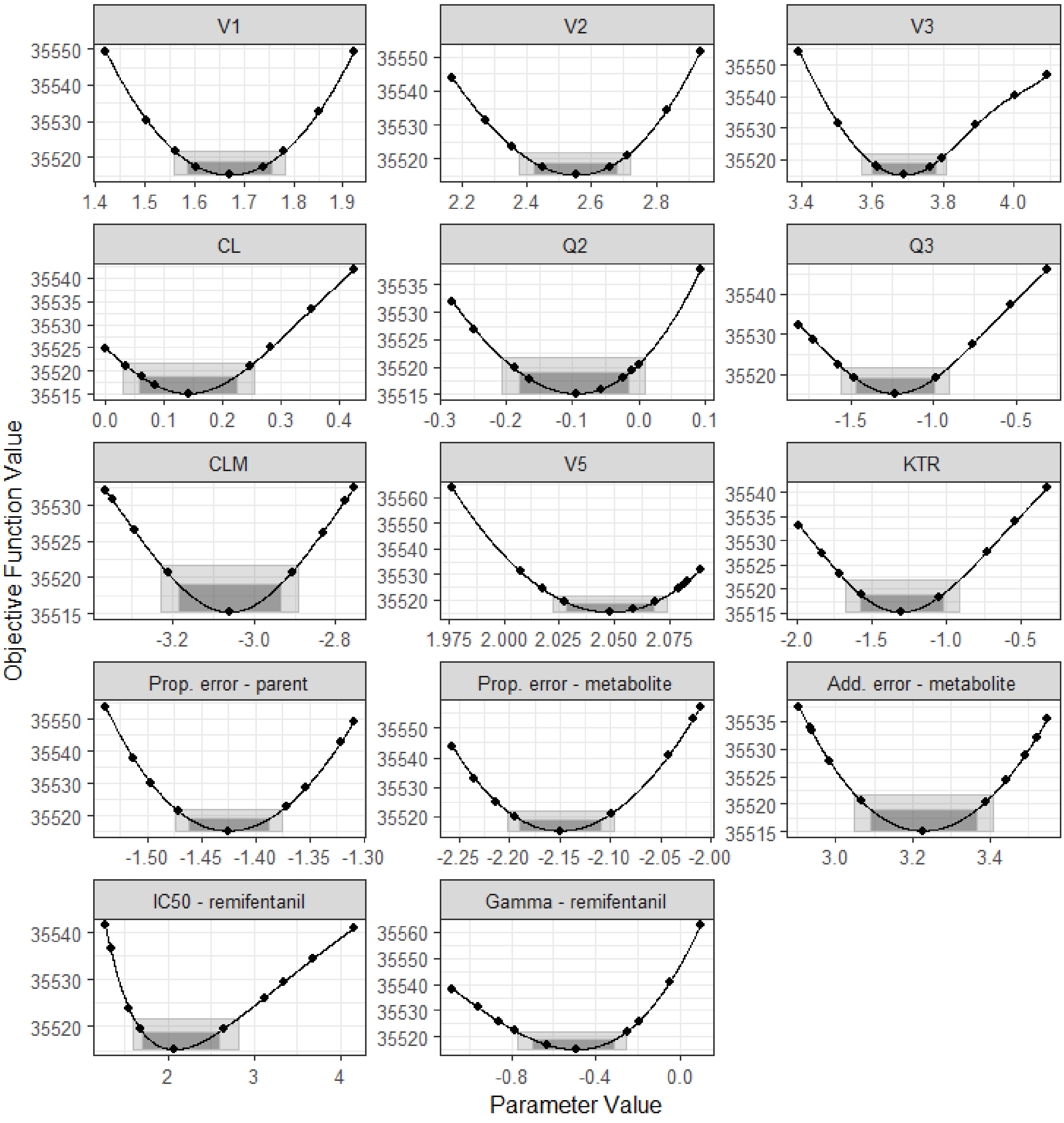

Figure 8. Log-likelihood profiles – OMEGAs – pharmacokinetic model

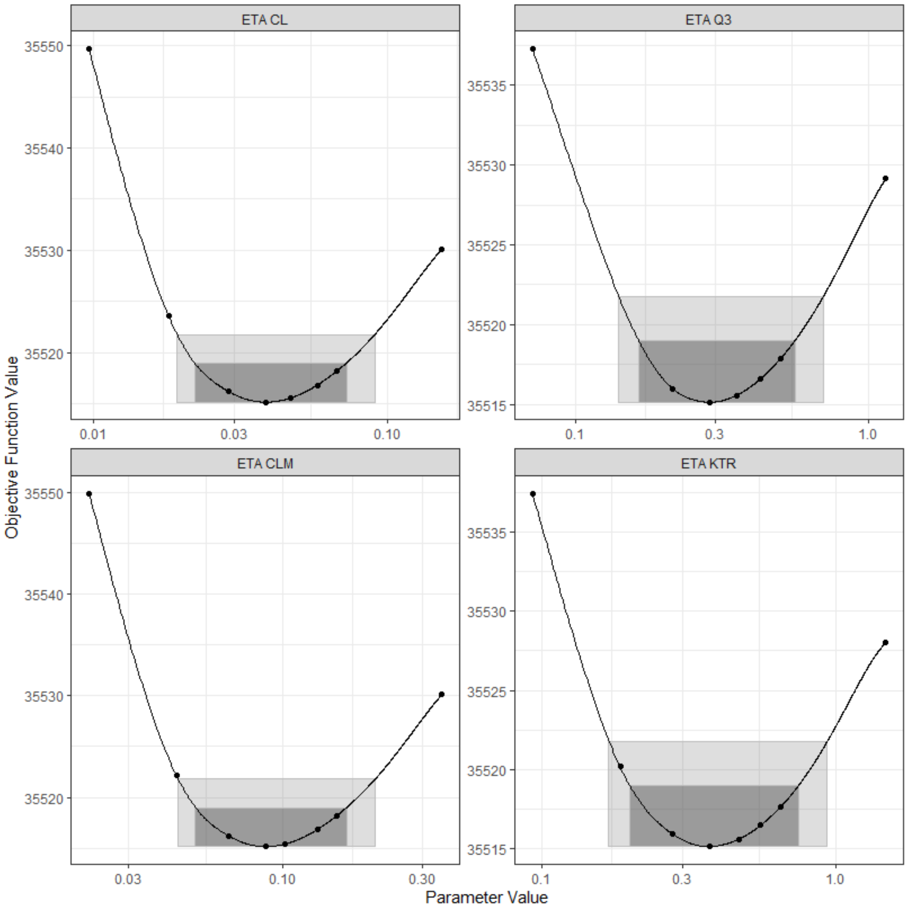

Model code – pharmacokinetic model

```
$PROB   …
$INPUT NMID=ID SESS RTIME DVTY AMT RATE EVID DV MDV BLQ AGE HEIGHT WEIGHT BMI SEX CMT
TIME
$DATA …
$SUBROUTINES ADVAN6 TOL=9
$MODEL
COMP=(CENTRAL)
COMP=(PERIPHERAL1)
COMP=(PERIPHERAL2)
COMP=(TRANSIT)
COMP=(METABOLITE)
COMP=(REMIFENTANIL)
COMP=(PERIREM1)
COMP=(PERIREM2)

;Pharmacokinetics
$PK (ONCE PER IR)
        ;Parameters:
                ;Covariates
                        ;Allometric Scaling
                        ALLCL =(WEIGHT/70)**0.75
                        ALLV = (WEIGHT/70)**1
                        ALLR = (WEIGHT/70)**(-0.25)
                        ALLT = (WEIGHT/70)**0.25

                ;Structural - Remimazolam:
                TVV1 = EXP(THETA(15)) * ALLV
                V1 = TVV1*EXP(ETA(7))

                TVV2 = EXP(THETA(16)) * ALLV
                V2 = TVV2*EXP(ETA(8))
```

```
TVV3 = EXP(THETA(17)) * ALLV
V3 = TVV3*EXP(ETA(9))

TVCL = EXP(THETA(18)) * ALLCL
CL = TVCL*EXP(ETA(10))

TVQ2 = EXP(THETA(19)) * ALLCL
Q2 = TVQ2*EXP(ETA(11))

TVQ3 = EXP(THETA(20)) * ALLCL
Q3 = TVQ3*EXP(ETA(12))

;Structural – CNS7054:
TVCLM = EXP(THETA(21)) * ALLCL
CLM = TVCLM*EXP(ETA(13))

TVV5 = EXP(THETA(22)) * ALLV
V5 = TVV5 * EXP(ETA(14))

TVKTR = EXP(THETA(23)) * ALLR
KTR = TVKTR * EXP(ETA(15))

;Remifentanil model (Eleveld Model)
        ;Maturation
        SE50=THETA(7)
        ADLT=(WEIGHT**2)/((WEIGHT**2)+(SE50**2))
        AREF=(70.**2)/((70.**2)+(SE50**2))
        KMAT=ADLT/AREF

        ; scaling using Al-sallami FFM
        HT2=(HEIGHT/100.)*(HEIGHT/100.)
        MATM=0.88+((1-0.88)/(1+(AGE/13.4)**(-12.7)))
        MATF=1.11+((1-1.11)/(1+(AGE/7.1)**(-1.1)))
```

```
FFMM=MATM*42.92*(HT2)*WEIGHT/(30.93*(HT2)+WEIGHT)
FFMF=MATF*37.99*(HT2)*WEIGHT/(35.98*(HT2)+WEIGHT)
FFMF=MATF*37.99*(HT2)*WEIGHT/(35.98*(HT2)+WEIGHT)
FFMR=42.92*(1.7*1.7)*70./(30.93*(1.7*1.7)+70.)
MAL=2-SEX
FEM=SEX-1
BSIZ=(MAL*FFMM + FEM*FFMF)/FFMR

; aging for v1/q2/q2, v3 and v2/cl
KV1=EXP(THETA(8)*(AGE-35.))
KV2=EXP(THETA(9)*(AGE-35.))
KV3=EXP(THETA(10)*(AGE-35.))

KCL=KV2
KQ2=KV1
KQ3=KV1

; sex correction for cl, v2and q2
PPUB=(AGE**6)/(AGE**6 + 12**6)
ELDY=(AGE**6)/(AGE**6 + 45**6)
KSEX=1+(SEX-1)*PPUB*(1-ELDY)*THETA(11)

; weight correction for V3W
WV3=EXP(THETA(12)*(WEIGHT-70.))

; compartmental allometric scaling
M1 =(BSIZ)**1 * KV1
M2 =(BSIZ)**1 * KV2 * KSEX
M3 =(BSIZ)**1 * KV3 * WV3

;Structural Remifentanil:
V1R =EXP(THETA(1)+ETA(1)) * M1
V2R =EXP(THETA(2)+ETA(2)) * M2
```

```
        V3R =EXP(THETA(3)+ETA(3)) * M3

        RV2=EXP(THETA(2))
        RV3=EXP(THETA(3))

        M4 =(BSIZ)**0.75 * KCL * KSEX * KMAT
        M5 =(V2R/RV2)**0.75 * KQ2 * KSEX
        M6 =(V3R/RV3)**0.75 * KQ3

        CLR =EXP(THETA(4)+ETA(4)) * M4
        Q2R =EXP(THETA(5)+ETA(5)) * M5
        Q3R =EXP(THETA(6)+ETA(6)) * M6

        ;Interaction
        IC50 = EXP(THETA(28))
        GAM = EXP(THETA(29))

;Scaling Parameters
        ;Parent:
        S1 = V1/1000
        FM = THETA(24)

        ;Metabolite:
        S5 = V5/1000

        ;Remifentanil:
        S6 = V1R

;Rate constants:
        ;Remimazolam:
        K10 = (CL*(1-FM))/V1
        K12 = Q2/V1
        K21 = Q2/V2
```

```
        K13 = Q3/V1
        K31 = Q3/V3

        ;Metabolite:
        K14 = (CL*FM)/V1
        K50 = CLM/V5
        K54 = KTR

        ;Remifentanil:
        K60 = CLR/V1R
        K67 = Q2R/V1R
        K76 = Q2R/V2R
        K68 = Q3R/V1R
        K86 = Q3R/V3R

    ;Error
        ;Remimazolam:
        PROP = EXP(THETA(25))
        ADD = 0

        ;CNS7054:
        PROPM = EXP(THETA(26))
        ADDM = EXP(THETA(27))

        ;Remifentanil:
        PROPR = EXP(THETA(13))
        ADDR = EXP(THETA(14))

;Differential Equations:
$DES
CONC = A(6)/V1R
INH = CONC**GAM / (CONC**GAM + IC50**GAM)
```

```
;Remimazolam:
DADT(1) = -K10*A(1) - K12*A(1) + K21*A(2) - K13*A(1) + K31*A(3) - K14*A(1)
DADT(2) =                   K12*A(1) - K21*A(2)
DADT(3) =                                                K13*A(1) - K31*A(3)

;CNS7054:
DADT(4) =                                                                               K14*A(1) - K54*A(4)
DADT(5) = -K50*A(5)*(1 - INH)                                                           + K54*A(4)

;Remifentanil:
DADT(6) = -K60*A(6) - K67*A(6) + K76*A(7) - K68*A(6) + K86*A(8)
DADT(7) =                   K67*A(6) - K76*A(7)
DADT(8) =                                                K68*A(6) - K86*A(8)

$ERROR
;Redefine variables in DES:
CP = A(1)/V1*1000
RATIO = 425.3/439.3                    ;MW[Metabolite]425.3 / MW[Parent] 439.3
CM = A(5)/V5*1000*RATIO
CR = A(6)/V1R

;Remimazolam - above LOQ
IF(DVTY.EQ.1.AND.BLQ.EQ.0) THEN
F_FLAG = 0
MDVRES = 0
IPRED = CP
SD = SQRT(ADD*ADD+PROP*PROP*CP*CP)
Y = CP + SD*EPS(1)
ENDIF

;Remimazolam - below LOQ
IF(DVTY.EQ.1.AND.BLQ.EQ.1) THEN
F_FLAG = 1
```

```
MDVRES = 1
IPRED = CP
SD = SQRT(ADD*ADD+PROP*PROP*CP*CP)
DUM = (2-IPRED)/SD
CDUM = PHI(DUM)
Y = CDUM
ENDIF

;CNS7054 - above LOQ
IF(DVTY.EQ.2.AND.BLQ.EQ.0) THEN
F_FLAG = 0
MDVRES = 0
IPRED = CM
SDM = SQRT(ADDM*ADDM+PROPM*PROPM*CM*CM)
Y = CM + SDM*EPS(1)
ENDIF

;CNS7054 - below LOQ
IF(DVTY.EQ.2.AND.BLQ.EQ.1) THEN
F_FLAG = 1
MDVRES = 1
IPRED = CM
SDM = SQRT(ADDM*ADDM+PROPM*PROPM*CM*CM)
DUM2 = (20-IPRED)/SDM
CDUM2 = PHI(DUM2)
Y = CDUM2
ENDIF

;Remifentanil - above LOQ
IF(DVTY.EQ.3.AND.BLQ.EQ.0) THEN
F_FLAG = 0
MDVRES = 0
IPRED = CR
```

```
SDR = SQRT(ADDR*ADDR+PROPR*PROPR*CR*CR)
Y = CR + SDR*EPS(1)
ENDIF

;Remifentanil - below LOQ
IF(DVTY.EQ.3.AND.BLQ.EQ.1) THEN
F_FLAG = 1
MDVRES = 1
IPRED = CR
SDR = SQRT(ADDR*ADDR+PROPR*PROPR*CR*CR)
DUM3 = (0.01-IPRED)/SDR
CDUM3 = PHI(DUM3)
Y = CDUM3
ENDIF

$THETA
(-1, 1.759110e+00, 3) FIX              ;V1=5.81
(-1, 2.177170e+00, 3) FIX              ;V2=8.82
(-1, 1.614450e+00, 3) FIX              ;V3=5.03
(0, 9.462570e-01, 2)  FIX              ;CL=2.58
(0, 5.403150e-01, 2)  FIX              ;Q2=1.72
(-4, -2.083880e+00, 1)FIX              ;Q3=0.12
(0.1, 2.878980e+00, 50) FIX            ;E50 for CL maturation
(-0.1, -5.544810e-03, 0.1) FIX         ;Aging v1/q2/q3
(-0.1, -3.269850e-03, 0.1) FIX         ;Aging v2/cl
(-0.1, -3.151350e-02, 0.1) FIX         ;Aging v3
(-1, 4.704050e-01, 1) FIX              ;Increase cl/q2/v2 in females 12-45 years
(-0.1, -2.604960e-02, 0.1) FIX         ;Weight correction v3
-1.4506 FIX                            ;Proportional error - remifentanil
-3.6352 FIX                            ;Add error - remifentanil
(-10, 1.7, 10)                         ;V1 - remimazolam parameters
(-10, 2.6, 10)                         ;V2
(-10, 3.7, 10)                         ;V3
```

```
(-10, 0.15, 10)              ;CL
(-10, -0.1, 10)              ;Q2
(-10, -1.2, 10)              ;Q3
(-10, -3.0, 10)              ;CLM
(-10, 2.0, 10)               ;V5
(-10, -1.3, 10)              ;KTR
0.8 FIX                      ;Fraction metabolised to EPAR (80%)
(-10, -1.40, 10)             ;PROP
(-10, -2.1, 10)              ;PROPM
(-10, 3.2, 10)               ;ADDM
(-10, 2.1, 10)               ;IC50
(-10, -0.5, 10)              ;GAMMA

$OMEGA
1.035760e-01 FIX        ;V1 CV=33.0%
1.153660e-01 FIX        ;V2 CV=35.0%
8.097060e-01 FIX        ;V3 CV=111.7%
1.973920e-02 FIX        ;CL CV=14.1%
5.466160e-02 FIX        ;Q2 CV=23.7%
2.854850e-01 FIX        ;Q3 CV=57.5%
0.0 FIX                 ;V1
0.0 FIX                 ;V2
0.0 FIX                 ;V3
0.04                    ;CL
0.0 FIX                 ;Q2
0.3                     ;Q3
0.08                    ;CLM
0.0 FIX                 ;V5
0.4                     ;KTR

$SIGMA
1.0 FIX                 ;Error
```

```
$ESTM METHOD=1 INTER NOABORT POSTHOC PRINT=1 MAXEVAL=9999

$COV …

$TABLE …
```
