# Supplementary material for: Influence of Remifentanil on the Pharmacokinetics and Pharmacodynamics of Remimazolam in Healthy Volunteers
Source: Anesthesiology. 2025 Jan 15;142(4):666–79. doi: 10.1097/ALN.0000000000005348 (PMC11892992; doi:10.1097/ALN.0000000000005348)
Supplement: Supplementary file 6 [file aln-142-666-s006.pdf]

Supplemental Digital Content 6

Development of the population pharmacodynamic model (BIS)

In total 891 BIS observations were collected in three periods. The population pharmacodynamic model for BIS that was previously developed based on data of period 1 was refitted to the BIS observations collected in all periods of the trial. The addition of a remifentanil interaction on *Drug effect* according to equation 2 improved the model (ΔOFV: -8.9 points, df = 1, p=0.002). EC50 (32.3 ng/mL) for remifentanil was estimated to be far higher than the maximum observed remifentanil concentration and the remifentanil effect was therefore, at this stage, removed from the model. Removal of the competitive antagonism by CNS7054 worsened the model fit (ΔOFV: 285.6 points, df = 1, p<0.001) and was therefore preserved in the model. Addition of inter-individual variability on the maximum drug effect parameter and changing the residual error model from an additive in the logit domain to a combined additive and proportional error model in the logit domain further improved the overall model fit (ΔOFV: -107.0 points, df = 2, p<0.001). Finally, the remifentanil interaction was re-evaluated. An additive interaction model on *Drug effect,* according to equation 6, did improve the model fit (ΔOFV: -26.3 points, df = 1, p<0.001). Again, removal of the competitive antagonism by CNS7054 was evaluated, but resulted in a worsening of model fit (ΔOFV: 325.3 points, df = 1, p<0.001). Visual predictive checks and likelihood profiles of the population pharmacodynamic model for BIS are shown in Figures 1 and 2, followed by the model code.

Figure 1. Prediction-corrected Visual Predictive Checks for BIS stratified by session. *Red solid line represents the median observations, blue dashed lines represent the 5th and 95th percentiles of the observations. Red shaded area represents the 95% confidence interval of the median prediction. Blue shaded areas are 95% confidence intervals of the model predictions of the 5th and 95th percentiles.*

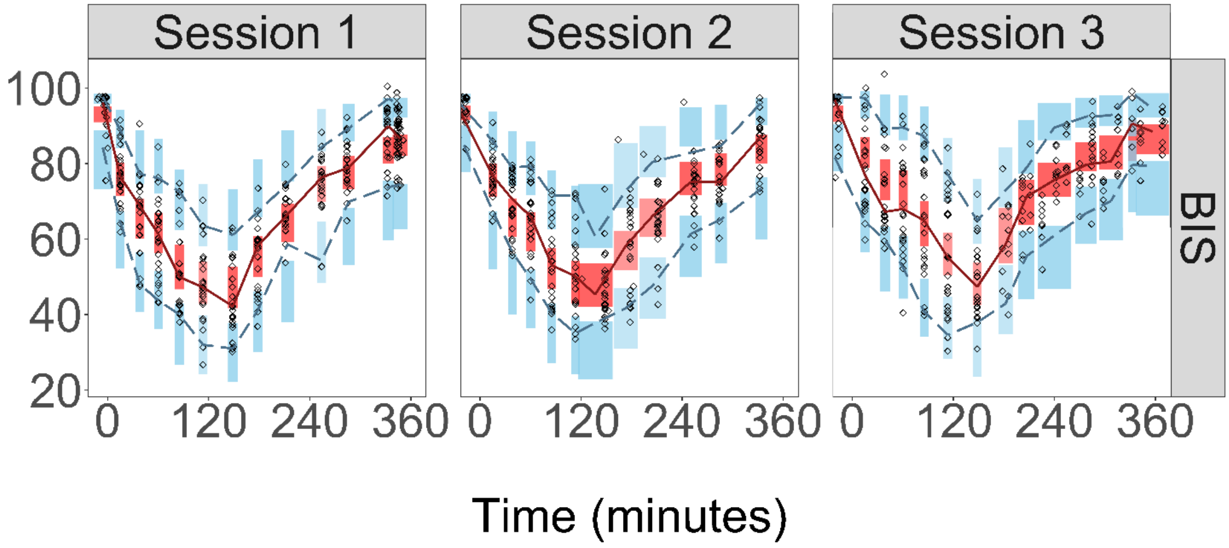

Session 1
Session 2
Session 3
100
80
60
40
20
0
120
240
360
BIS
Time (minutes)

Figure 2. Loglikelihood profiles – BIS

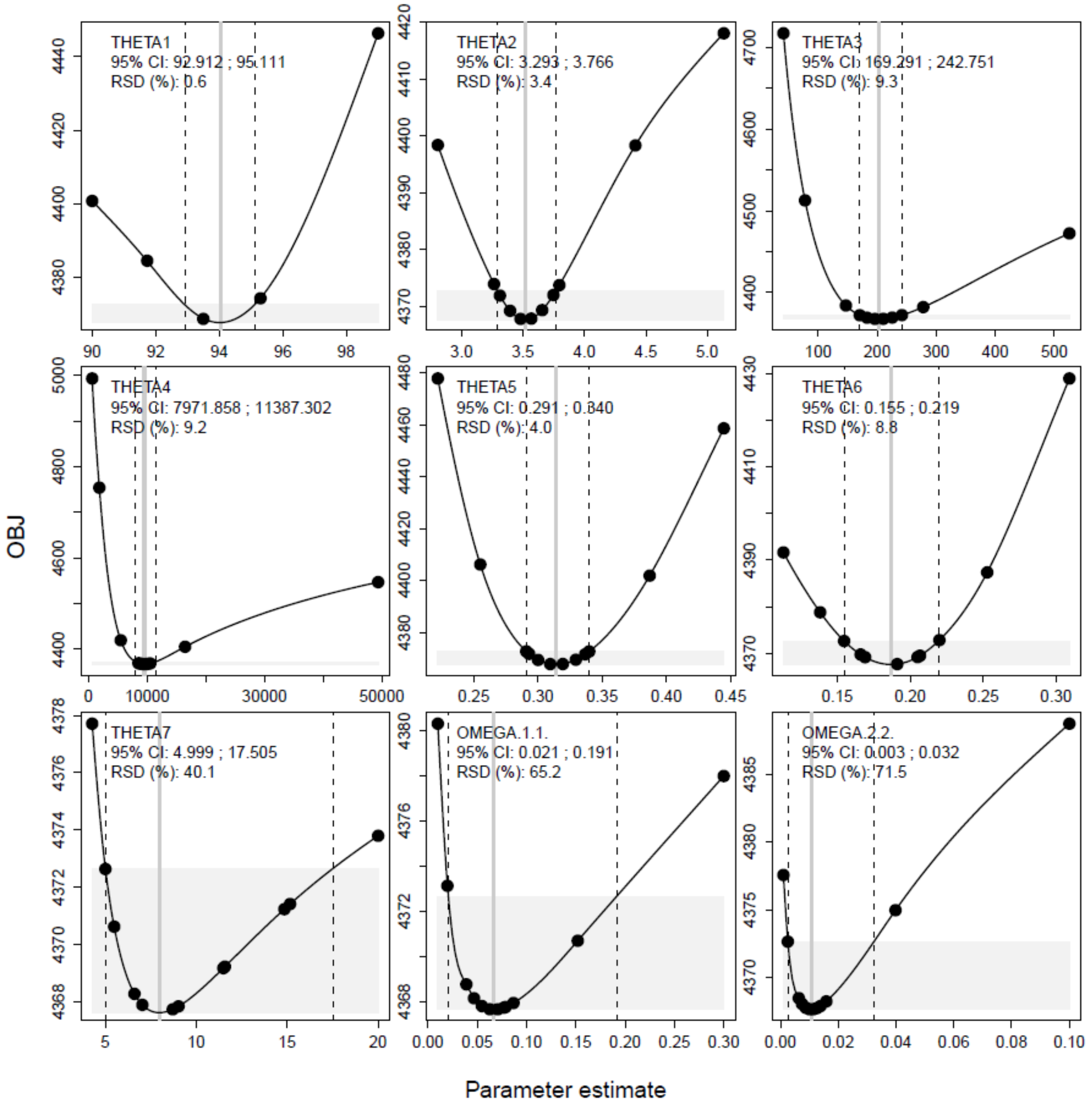

Model code – pharmacodynamic model (BIS)

```
$PROB   ….

$INPUT NMID=ID SESS RTIME DVTY AMT RATE EVID DV MDV BLQ AGE HEIGHT WEIGHT BMI SEX
CMT TIME IV1 IV2 IV3 ICL IQ2 IQ3 ICLM IV5 IKTR IIC50 IGAM IV1R IV2R IV3R ICLR IQ2R IQ3R

$DATA ….

$SUBROUTINES ADVAN6 TOL=9

$MODEL
COMP=(CENTRAL)
COMP=(PERIPHERAL1)
COMP=(PERIPHERAL2)
COMP=(TRANSIT)
COMP=(METABOLITE)
COMP=(REMIFENTANIL)
COMP=(PERIREM1)
COMP=(PERIREM2)


;Pharmacokinetics
$PK (ONCE PER IR)
        ;Parameters:
                ;Structural - Parent:
                V1 = IV1
                V2 = IV2
                V3 = IV3
                CL = ICL
                Q2 = IQ2
                Q3 = IQ3

                ;Structural - Metabolite:
                CLM = ICLM
```

```
V5 = IV5
KTR = IKTR

;Remifentanil model (Eleveld Model)
    V1R = IV1R
    V2R = IV2R
    V3R = IV3R
    CLR = ICLR
    Q2R = IQ2R
    Q3R = IQ3R

;Scaling Parameters
    ;Parent:
    S1 = V1/1000
    FM = 0.8

    ;Metabolite:
    S5 = V5/1000

    ;Remifentanil:
    S6 = V1R

;Rate constants:
    ;Remimazolam:
    K10 = (CL*(1-FM))/V1
    K12 = Q2/V1
    K21 = Q2/V2
    K13 = Q3/V1
    K31 = Q3/V3
```

```
;Metabolite:
K14 = (CL*FM)/V1
K50 = CLM/V5
K54 = KTR

;Remifentanil:
K60 = CLR/V1R
K67 = Q2R/V1R
K76 = Q2R/V2R
K68 = Q3R/V1R
K86 = Q3R/V3R

;Interaction
IC50 = IIC50
GAM = IGAM
;BIS
;Structural
BASEB = EXP(THETA(1))
EMAXB = EXP(THETA(2)+ETA(2))
EC50B = EXP(THETA(3))
EC50MB = EXP(THETA(4))
EC50RB = EXP(THETA(7))
LBASE = LOG(BASEB/(100 - BASEB)) + ETA(1)

;Error
ADDB = EXP(THETA(5))
PROPB = EXP(THETA(6))
```

```
;Differential Equations:

$DES

CONC = A(6)/V1R

INH = 0

IF(CONC.GT.0) INH = CONC**GAM / (CONC**GAM + IC50**GAM)


;Remimazolam:

DADT(1) = -K10*A(1) - K12*A(1) + K21*A(2) - K13*A(1) + K31*A(3) - K14*A(1)

DADT(2) =                     K12*A(1) - K21*A(2)

DADT(3) =                                                K13*A(1) - K31*A(3)


;Metabolite:

DADT(4) =                                                                     K14*A(1) - K54*A(4)

DADT(5) = -K50*A(5)*(1 - INH)                                                     + K54*A(4)


;Remifentanil:

DADT(6) = -K60*A(6) - K67*A(6) + K76*A(7) - K68*A(6) + K86*A(8)

DADT(7) =                    K67*A(6) - K76*A(7)

DADT(8) =                                                 K68*A(6) - K86*A(8)


$ERROR

;Redefine variables in DES:

CP = A(1)/V1*1000

RATIO = 425.3/439.3                              ;MW[Metabolite]425.3 / MW[Parent] 439.3

CM = A(5)/V5*1000*RATIO

CR = A(6)/V1R


;BIS:

            ;Structural:
```

```
    CA = 0
    CB = 0
    DRUGB = 0
    IF(CP.GT.0) CA = CP/EC50B
    IF(CM.GT.0) CB = CM/EC50MB
    IF(CR.GT.0) CC = CR/EC50RB
    IF(CP.GT.0) DRUGB = EMAXB * (CA + CC)/(1 + CA + CB + CC)

    ;Mapping to data:
    IF(DVTY.EQ.9) THEN
    SDB = SQRT(ADDB*ADDB+PROPB*PROPB*(LBASE - DRUGB)*(LBASE - DRUGB))
    ERRB = SDB * EPS(1)
    IPRED = 100 * (EXP(LBASE - DRUGB)/(1 + EXP(LBASE - DRUGB)))
    Y = 100 * (EXP(LBASE - DRUGB + ERRB)/(1 + EXP(LBASE - DRUGB + ERRB)))
    ENDIF

$THETA
(0, 4.54, 4.60517)      ;BASEB
(0, 1.2)                ;EMAXB
(0, 5.2)                ;EC50B
(0, 9.0)                ;EC50MB
-0.9                    ;ADDB
-3                      ;PROPB
2.08                    ; EC50RB

$OMEGA
0.1                             ;ETA BASE
0.03                            ;ETA EMAX
```

```
$SIGMA
1.0 FIX                    ;Error

$ESTM METHOD=1 INTER NOABORT POSTHOC PRINT=1 MAXEVAL=9999
$TABLE ….
```

Development of the population pharmacodynamic model (MOAAS Score)

In total, 1025 MOAAS observations were collected in three periods. The population pharmacodynamic model for MOAAS that was previously developed based on data of period 1 was refitted to the MOAAS observations collected in all periods of the trial. The addition of a remifentanil interaction on *Drug effect* according to equation 2 improved the model fit (ΔOFV: -171.6 points, df = 2, $p<0.001$). Removal of the competitive antagonism by CNS7054 in the model decreased the model fit (ΔOFV: 328.4 points, df = 1, $p<0.001$). Subsequently, the inter-individual variability part of the model was re-evaluated. Replacing the inter-individual variability parameter of the maximum drug effect with inter-individual variability on the baseline parameters of the model showed an additional improvement in model fit (ΔOFV: -29.8 points, df = 0, $p<0.001$). Visual predictive checks and likelihood profiles of the population pharmacodynamic model for MOAAS are shown in Figures 3 to 5, followed by the model code.

Figure 3. Visual predictive check for the modified observer's assessment of alertness or sedation (MOAAS) score stratified by session. *Black dashed line represents the median observations*. *Blue shaded areas are 95% confidence intervals of the model predictions.*

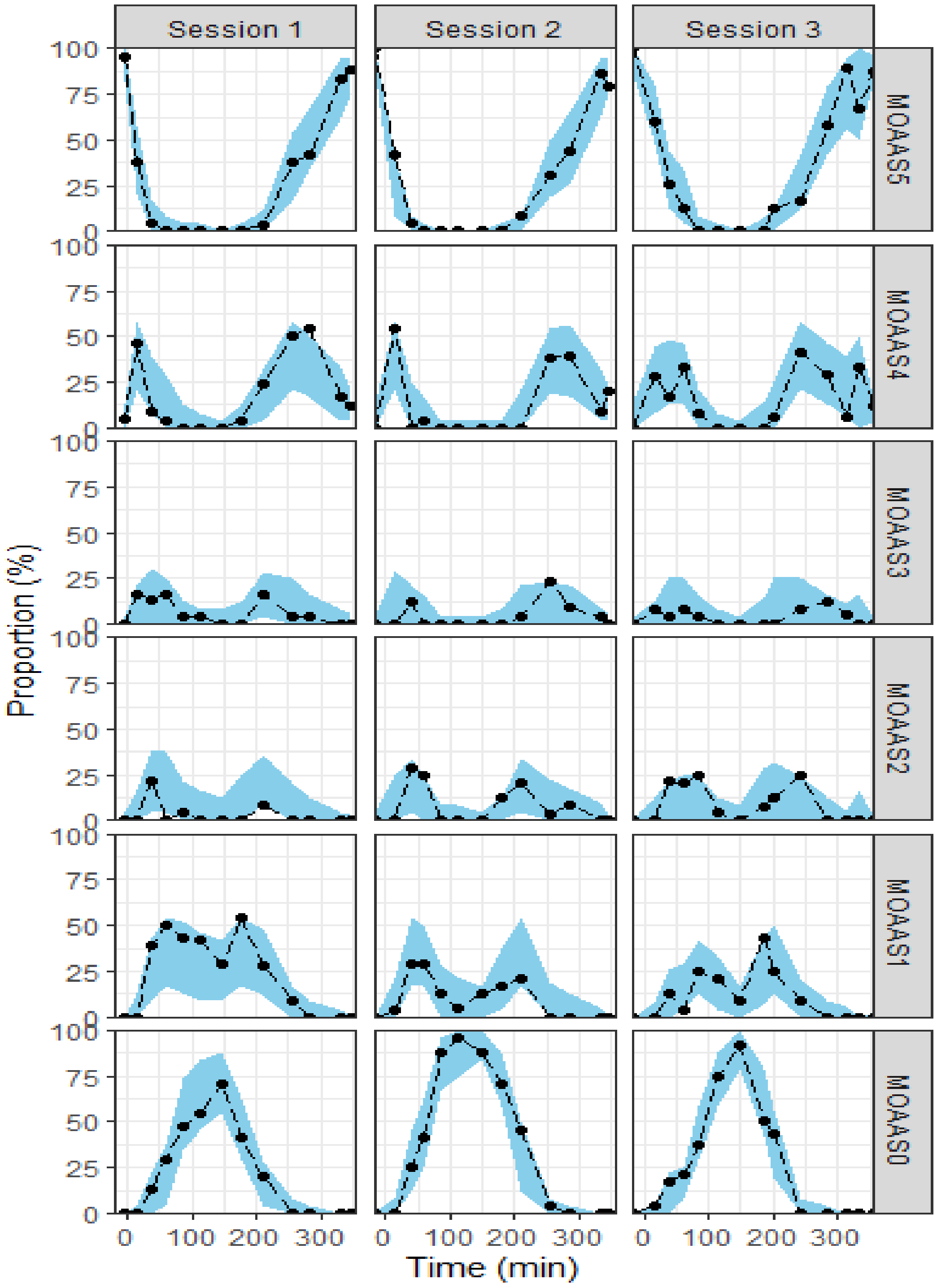

Figure 4. Log-likelihood profiles – THETAs – MOAAS Score

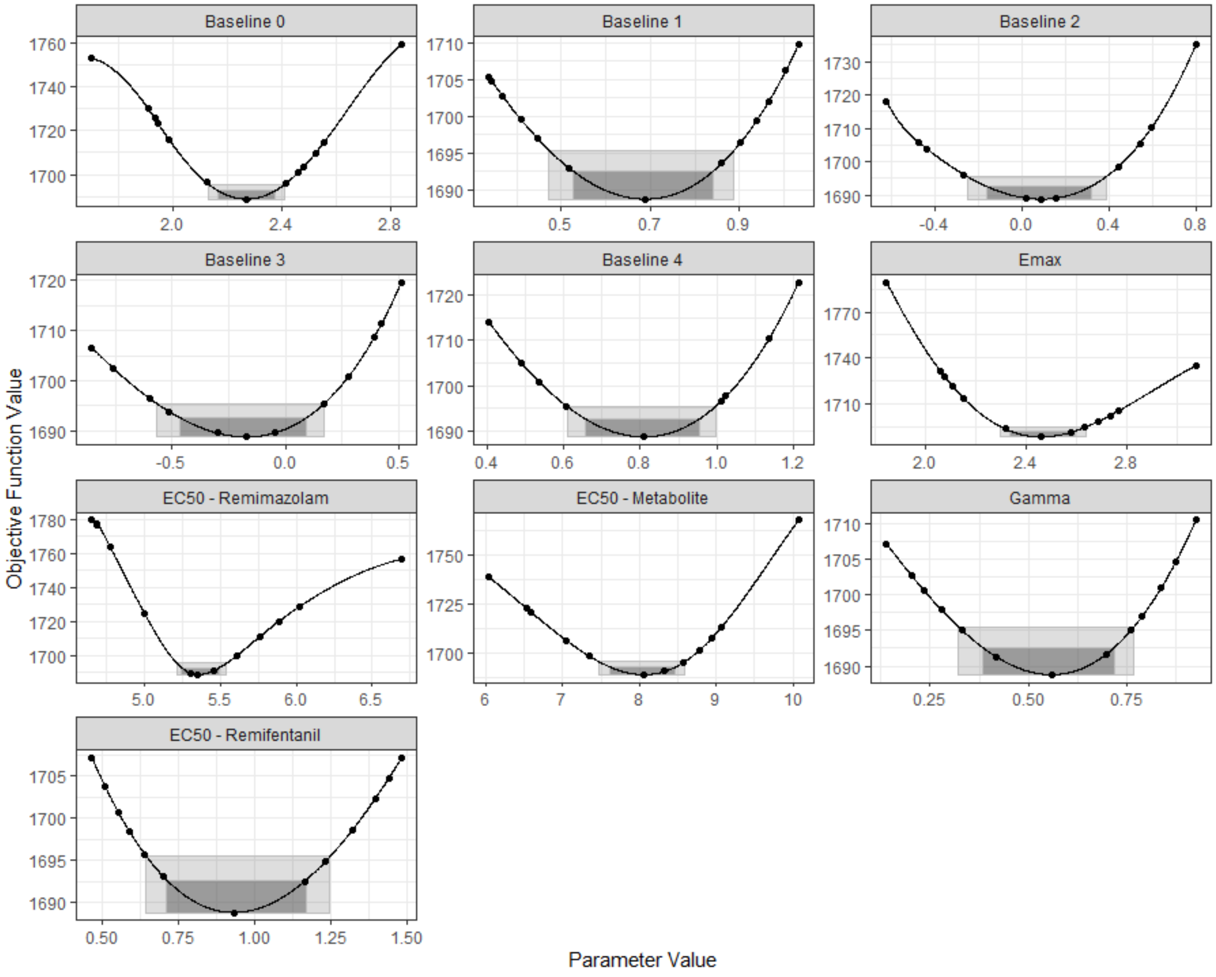

Figure 5. Log-likelihood profiles – OMEGAs – MOAAS Score

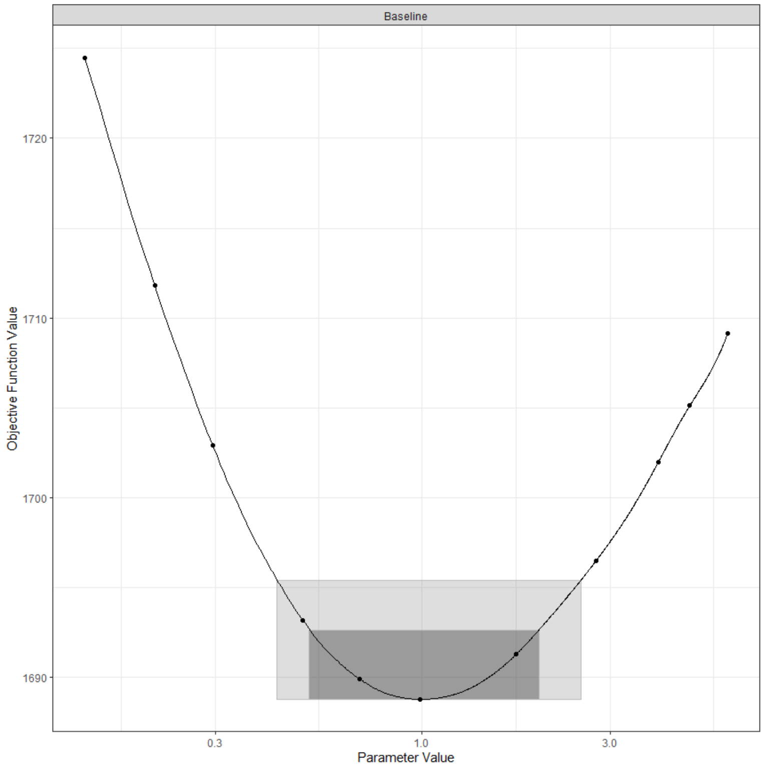

Model code – pharmacodynamic model (MOAAS Score)

```
$PROB   ….

$INPUT NMID=ID SESS RTIME DVTY AMT RATE EVID DV MDV BLQ AGE HEIGHT WEIGHT BMI SEX
CMT TIME IV1 IV2 IV3 ICL IQ2 IQ3 ICLM IV5 IKTR IIC50 IGAM IV1R IV2R IV3R ICLR IQ2R IQ3R

$DATA ….

$SUBROUTINES ADVAN6 TOL=9

$MODEL
COMP=(CENTRAL)
COMP=(PERIPHERAL1)
COMP=(PERIPHERAL2)
COMP=(TRANSIT)
COMP=(METABOLITE)
COMP=(REMIFENTANIL)
COMP=(PERIREM1)
COMP=(PERIREM2)


;Pharmacokinetics
$PK (ONCE PER IR)
        ;Parameters:
                ;Structural - Parent:
                V1 = IV1
                V2 = IV2
                V3 = IV3
                CL = ICL
                Q2 = IQ2
                Q3 = IQ3

                ;Structural - Metabolite:
                CLM = ICLM
```

```
V5 = IV5

KTR = IKTR


;Remifentanil model (Eleveld Model)

        V1R = IV1R

        V2R = IV2R

        V3R = IV3R

        CLR = ICLR

        Q2R = IQ2R

        Q3R = IQ3R


;Scaling Parameters

        ;Parent:

        S1 = V1/1000

        FM = 0.8


        ;Metabolite:

        S5 = V5/1000


        ;Remifentanil:

        S6 = V1R


;Rate constants:

        ;Remimazolam:

        K10 = (CL*(1-FM))/V1

        K12 = Q2/V1

        K21 = Q2/V2

        K13 = Q3/V1

        K31 = Q3/V3
```

```
;Metabolite:
K14 = (CL*FM)/V1
K50 = CLM/V5
K54 = KTR

;Remifentanil:
K60 = CLR/V1R
K67 = Q2R/V1R
K76 = Q2R/V2R
K68 = Q3R/V1R
K86 = Q3R/V3R

;Interaction
IC50 = IIC50
GAM = IGAM
;MOAAS
;Structural:
BASE0 = EXP(THETA(1))
BASE1 = EXP(THETA(2))
BASE2 = EXP(THETA(3))
BASE3 = EXP(THETA(4))
BASE4 = EXP(THETA(5))
EMAXM = EXP(THETA(6))
EC50M = EXP(THETA(7))
EC50MM = EXP(THETA(8))
GAMMAM = EXP(THETA(9))
ETA_BASE = ETA(1)
EC50MR = EXP(THETA(10))
```

```
;Differential Equations:
$DES
CONC = A(6)/V1R
INH = 0
IF(CONC.GT.0) INH = CONC**GAM / (CONC**GAM + IC50**GAM)

;Remimazolam:
DADT(1) = -K10*A(1) - K12*A(1) + K21*A(2) - K13*A(1) + K31*A(3) - K14*A(1)
DADT(2) =                     K12*A(1) - K21*A(2)
DADT(3) =                                                  K13*A(1) - K31*A(3)

;Metabolite:
DADT(4) =                                                                                     K14*A(1) - K54*A(4)
DADT(5) = -K50*A(5)*(1 - INH)                                                                            + K54*A(4)


;Remifentanil:
DADT(6) = -K60*A(6) - K67*A(6) + K76*A(7) - K68*A(6) + K86*A(8)
DADT(7) =                    K67*A(6) - K76*A(7)
DADT(8) =                                                    K68*A(6) - K86*A(8)

$ERROR
;Redefine variables in DES:
CP = A(1)/V1*1000
RATIO = 425.3/439.3                              ;MW[Metabolite]425.3 / MW[Parent] 439.3
CM = A(5)/V5*1000*RATIO
CR = A(6)/V1R


            ;Logits:
```

```
LLE0 = (-BASE0)                                    ;Logit(P(Y=1 | Y<1))
LLE1 = (-BASE0) + BASE1                            ;Logit(P(Y=2 | Y<2))
LLE2 = (-BASE0) + BASE1 + BASE2                    ;Logit(P(Y=3 | Y<3))
LLE3 = (-BASE0) + BASE1 + BASE2 + BASE3            ;Logit(P(Y=4 | Y<4))
LLE4 = (-BASE0) + BASE1 + BASE2 + BASE3 + BASE4    ;Logit(P(Y=5 | Y<5))

;Predictor functions:
CAM = 0
CBM = 0
CCM = 0
IF(CP.GT.0) CAM = (CP/EC50M)**GAMMAM
IF(CM.GT.0) CBM = (CM/EC50MM)
IF(CR.GT.0) CCM = (CR/EC50MR)
DRUGM = EMAXM * (CAM/(1 + CAM + CBM)) * (1 + CCM)

;Add Predictors to baseline logits:
LLE0 = LLE0 + DRUGM + ETA_BASE
LLE1 = LLE1 + DRUGM + ETA_BASE
LLE2 = LLE2 + DRUGM + ETA_BASE
LLE3 = LLE3 + DRUGM + ETA_BASE
LLE4 = LLE4 + DRUGM + ETA_BASE

;Cumulative Probabilities: Expit to ensure 0-1 scale
PLE0 = EXP(LLE0) / (1 + EXP(LLE0))                 ;P(Y=0 | Y<0)
PLE1 = EXP(LLE1) / (1 + EXP(LLE1))                 ;P(Y=1 | Y<1)
PLE2 = EXP(LLE2) / (1 + EXP(LLE2))                 ;P(Y=2 | Y<2)
PLE3 = EXP(LLE3) / (1 + EXP(LLE3))                 ;P(Y=3 | Y<3)
PLE4 = EXP(LLE4) / (1 + EXP(LLE4))                 ;P(Y=4 | Y<4)
```

```
        ;Probability per MOAAS score:
        P0 = PLE0                    ;P(Y=0 | Y<0) = P(Y=0)
        P1 = PLE1 - PLE0             ;P(Y=1 | Y<1) - P(Y=0 | Y>1) = P(Y=1)
        P2 = PLE2 - PLE1             ;P(Y=2 | Y<2) - P(Y=1 | Y>1) = P(Y=2)
        P3 = PLE3 - PLE2             ;P(Y=3 | Y<3) - P(Y=2 | Y>2) = P(Y=3)
        P4 = PLE4 - PLE3             ;P(Y=4 | Y<4) - P(Y=3 | Y>3) = P(Y=4)
        P5 = 1 - PLE4                ;1 - P(Y=4 | Y<4)  = P(Y=5)

        ;Estimate probabilities from data:
        IF(DVTY.EQ.4.AND.DV.EQ.5) Y = P5
        IF(DVTY.EQ.4.AND.DV.EQ.4) Y = P4
        IF(DVTY.EQ.4.AND.DV.EQ.3) Y = P3
        IF(DVTY.EQ.4.AND.DV.EQ.2) Y = P2
        IF(DVTY.EQ.4.AND.DV.EQ.1) Y = P1
        IF(DVTY.EQ.4.AND.DV.EQ.0) Y = P0

$THETA
2.1                 ;BASE0
0.5                 ;BASE1
-0.1                ;BASE2
-0.4                ;BASE3
0.6                 ;BASE4
2.4                 ;EMAXM
5.3                 ;EC50M
8.0                 ;EC50MM
0.5                 ;GAMMAM
-0.3                ;EC50R

$OMEGA
```

0.01 ;ETA Baseline

$ESTM METHOD=1 LAPLACIAN LIKELIHOOD NUMERICAL SLOW NOABORT POSTHOC PRINT=1 MAXEVAL=9999

$TABLE ….

Development of the population pharmacodynamic model (Tolerance to laryngoscopy)

In total 374 TOL observations were collected in three periods. Model development for TOL started by fitting a logistic regression model with inter-individual variability on baseline, but without a remimazolam drug effect. The addition of a remimazolam drug effect did not improve the overall model fit (ΔOFV: -0.3 points, df = 1, p=0.58). A remimazolam drug effect combined with a remifentanil interaction on $C50_{Remimazolam}$ did improve the model (ΔOFV: -83.1 points, df = 2, p<0.001). Competitive inhibition by CNS7054 was not supported by the TOTS data (ΔOFV: -1.4 points, df = 1, p=0.24). Replacing the inter-individual variability parameter on baseline with inter-individual variability on the $C50_{remimazolam}$ parameter of the model showed an additional improvement in overall model fit (ΔOFV: -22.2 points, df = 0, p<0.001). Likelihood profiling demonstrated that the baseline parameter (*baseline*) was not different from 1 at the 5% level of significance, indicating that the probability for observing tolerance to tetanic stimulation in the absence of drug effect is 0%. Therefore, the baseline probability in the population pharmacodynamic model was fixed to 0%. Visual predictive checks and likelihood profiles of the population pharmacodynamic model for TOL are shown in Figures 6 to 8, followed by the model code.

Figure 6. Visual predictive check for tolerance to laryngoscopy (TOL) stratified by session. *Black dashed line represents the median observations*. *Blue shaded areas are 95% confidence intervals of the model predictions.*

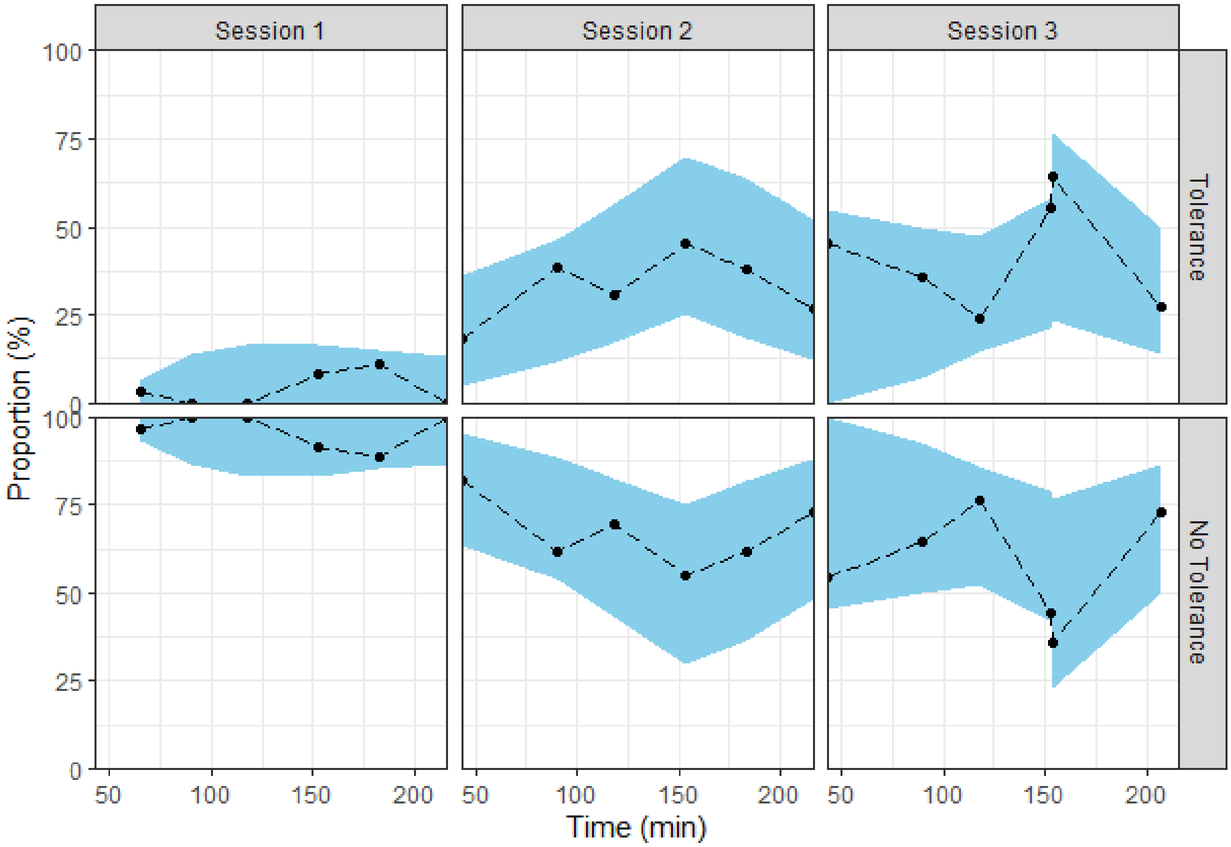

Figure 7. Log-likelihood profiles – THETAs – TOL

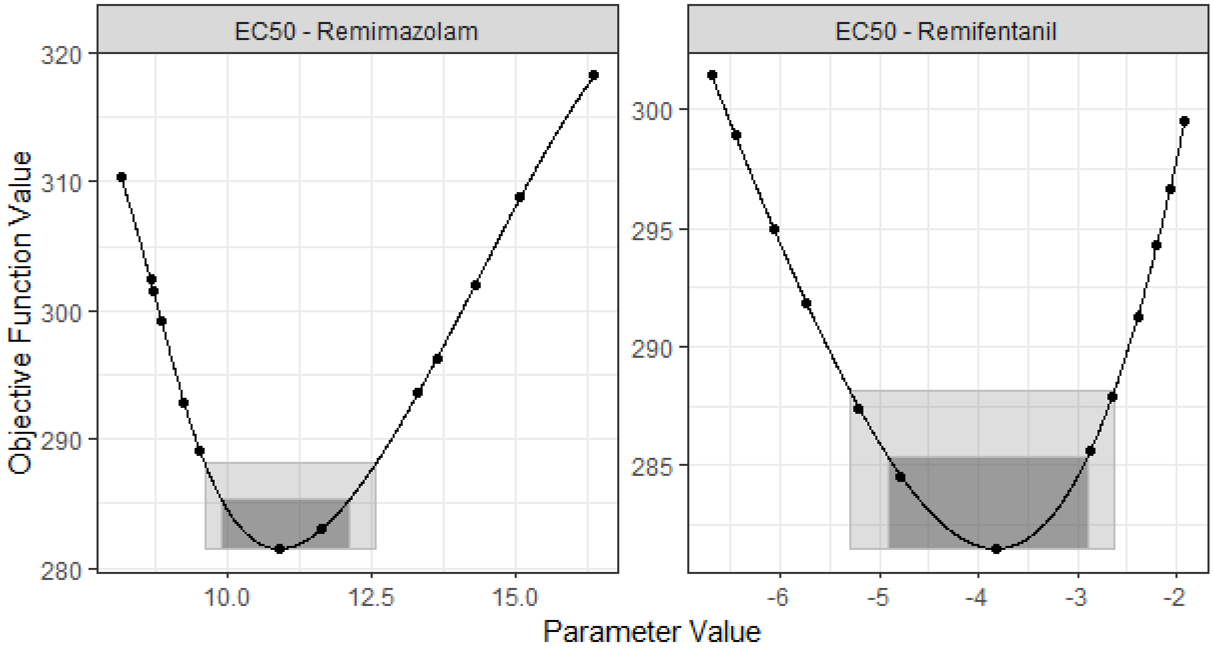

Figure 8. Log-likelihood profiles – OMEGAs – TOL

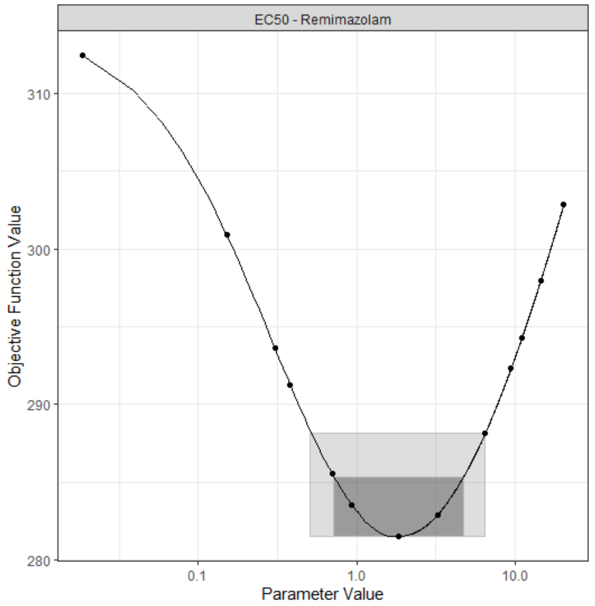

Model code – pharmacodynamic model (TOL)

```
$PROB   ….

$INPUT NMID=ID SESS RTIME DVTY AMT RATE EVID DV MDV BLQ AGE HEIGHT WEIGHT BMI SEX

CMT TIME IV1 IV2 IV3 ICL IQ2 IQ3 ICLM IV5 IKTR IIC50 IGAM IV1R IV2R IV3R ICLR IQ2R IQ3R

$DATA ….

$SUBROUTINES ADVAN6 TOL=9

$MODEL

COMP=(CENTRAL)

COMP=(PERIPHERAL1)

COMP=(PERIPHERAL2)

COMP=(TRANSIT)

COMP=(METABOLITE)

COMP=(REMIFENTANIL)

COMP=(PERIREM1)

COMP=(PERIREM2)


;Pharmacokinetics

$PK (ONCE PER IR)

        ;Parameters:

                ;Structural - Parent:

                V1 = IV1

                V2 = IV2

                V3 = IV3

                CL = ICL

                Q2 = IQ2

                Q3 = IQ3


                ;Structural - Metabolite:

                CLM = ICLM
```

```
V5 = IV5

KTR = IKTR


;Remifentanil model (Eleveld Model)

        V1R = IV1R

        V2R = IV2R

        V3R = IV3R

        CLR = ICLR

        Q2R = IQ2R

        Q3R = IQ3R


;Scaling Parameters

        ;Parent:

        S1 = V1/1000

        FM = 0.8


        ;Metabolite:

        S5 = V5/1000


        ;Remifentanil:

        S6 = V1R


;Rate constants:

        ;Remimazolam:

        K10 = (CL*(1-FM))/V1

        K12 = Q2/V1

        K21 = Q2/V2

        K13 = Q3/V1

        K31 = Q3/V3
```

```
;Metabolite:
K14 = (CL*FM)/V1
K50 = CLM/V5
K54 = KTR

;Remifentanil:
K60 = CLR/V1R
K67 = Q2R/V1R
K76 = Q2R/V2R
K68 = Q3R/V1R
K86 = Q3R/V3R

;Interaction
IC50 = IIC50
GAM = IGAM
;TOL
;Structural
EC50T = EXP(THETA(1)+ETA(1))
EC50TR = EXP(THETA(2))
;Differential Equations:
$DES
CONC = A(6)/V1R
INH = 0
IF(CONC.GT.0) INH = CONC**GAM / (CONC**GAM + IC50**GAM)

;Remimazolam:
DADT(1) = -K10*A(1) - K12*A(1) + K21*A(2) - K13*A(1) + K31*A(3) - K14*A(1)
DADT(2) =           K12*A(1) - K21*A(2)
```

```
DADT(3) =                                            K13*A(1) - K31*A(3)

;Metabolite:
DADT(4) =                                                                       K14*A(1) - K54*A(4)
DADT(5) = -K50*A(5)*(1 - INH)                                                              + K54*A(4)

;Remifentanil:
DADT(6) = -K60*A(6) - K67*A(6) + K76*A(7) - K68*A(6) + K86*A(8)
DADT(7) =                   K67*A(6) - K76*A(7)
DADT(8) =                                             K68*A(6) - K86*A(8)

$ERROR
;Redefine variables in DES:
CP = A(1)/V1*1000
RATIO = 425.3/439.3                              ;MW[Metabolite]425.3 / MW[Parent] 439.3
CM = A(5)/V5*1000*RATIO
CR = A(6)/V1R

;Tol:
        ;Structural:
        CA = 0
        CB = 0
        DRUGB = 0
        IF(CP.GT.0) CA = CP/(EC50T*(1 - CR/(CR + EC50TR)))
        IF(CM.GT.0) CB = 0
        IF(CP.GT.0) DRUGT = CA/(1 + CA + CB)

        ;Mapping to data:
        P0 = 1 * (1 - DRUGT)
```

```
    IF(DVTY.EQ.8.AND.DV.EQ.0) Y = P0

    IF(DVTY.EQ.8.AND.DV.EQ.1) Y = 1 - P0


$THETA

5.5                 ;EC50T

0.3                 ;EC50TR


$OMEGA

0.1                 ;ETA EC50T

$ESTM METHOD=1 LAPLACIAN LIKELIHOOD NUMERICAL SLOW NOABORT POSTHOC PRINT=1
MAXEVAL=9999

$TABLE ….
```

Development of the population pharmacodynamic model (Tolerance to tetanic stimulus)

In total 611 TOTS observations were collected in three periods. Model development for TOTS initiated by fitting a logistic regression model with inter-individual variability on the baseline, but without a remimazolam drug effect. The addition of a remimazolam drug effect improved the model (ΔOFV: -55.5 points, df = 1, p<0.001). An additional remifentanil interaction on the $C50_{Remimazolam}$ according to equation 5 further improved the model fit (ΔOFV: -134.2 points, df = 1, p<0.001). Competitive inhibition by CNS7054 was not supported by the TOTS data (ΔOFV: -1.5 points, df = 1, p=0.22). Replacing the inter-individual variability parameter of baseline with inter-individual variability on $C50_{Remimazolam}$ improved the model (ΔOFV: -26.5 points, df = 0, p<0.001). Likelihood profiling demonstrated that the baseline parameter (*baseline*) was not different from 1 at the 5% level of significance, indicating that the probability for observing tolerance to tetanic stimulation in the absence of drug effect is 0%. Therefore, the baseline probability in the population pharmacodynamic model was fixed to 0%. Visual predictive checks and likelihood profiles of the population pharmacodynamic model for TOTS are shown in Figures 9 to 11, followed by the model code.

Figure 9. Visual predictive check for tolerance to tetanic stimulus (TOTS) stratified by session. *Black dashed line represents the median observations. Blue shaded areas are 95% confidence intervals of the model predictions.*

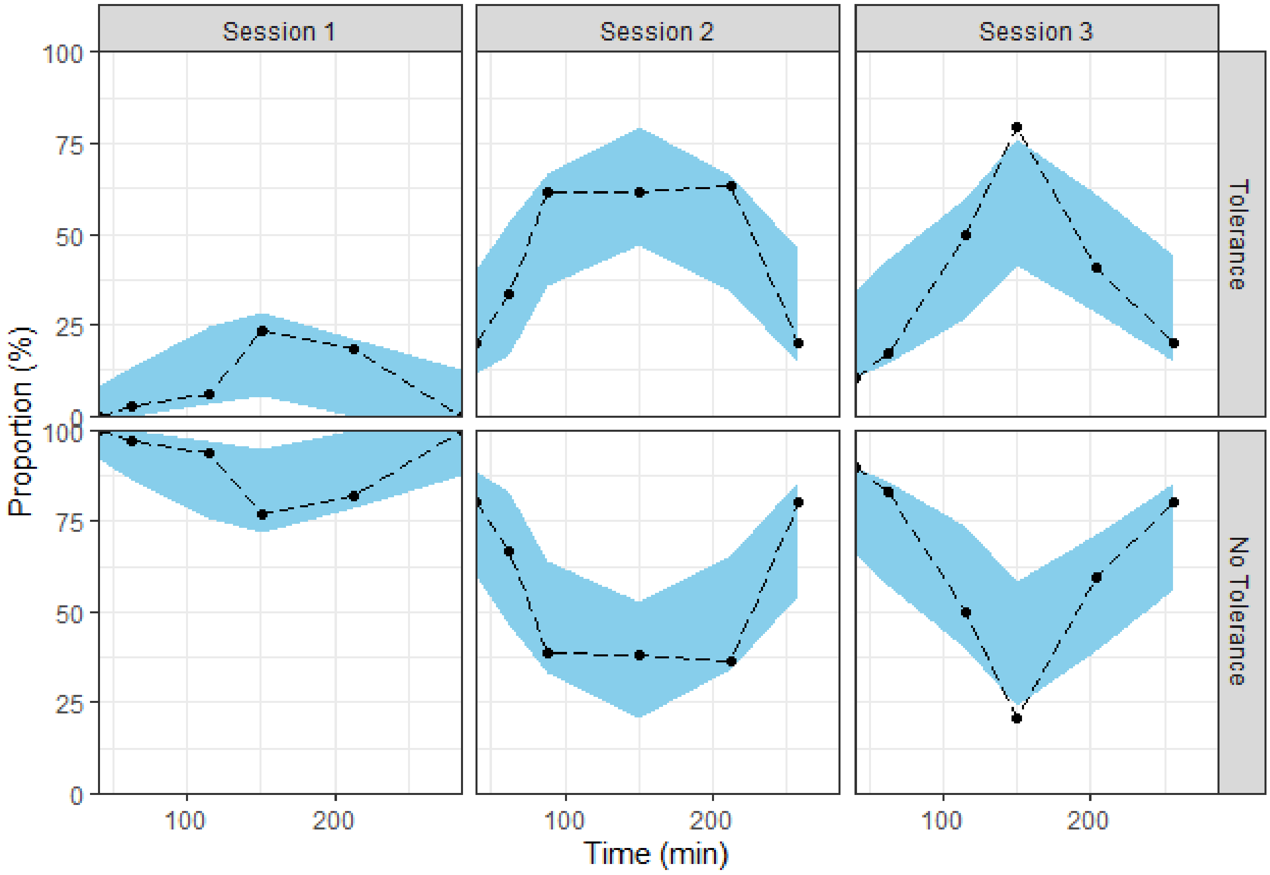

Figure 10. Log-likelihood profiles – THETAs – TOTS

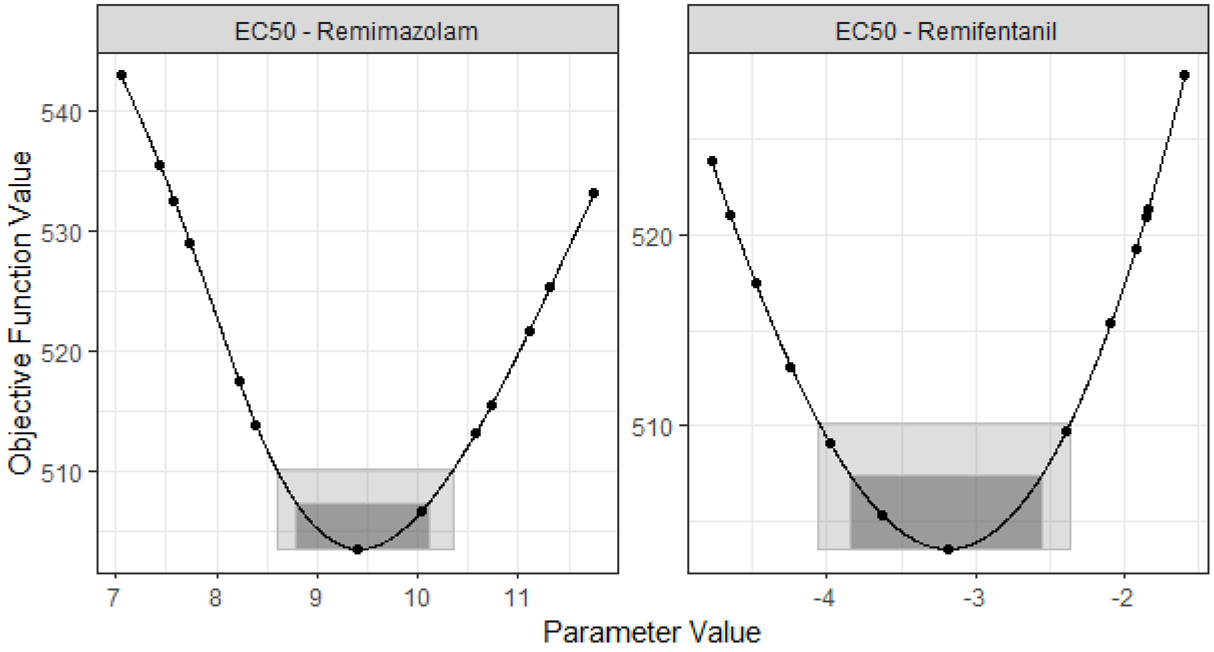

Figure 11. Log-likelihood profiles – OMEGAs – TOTS

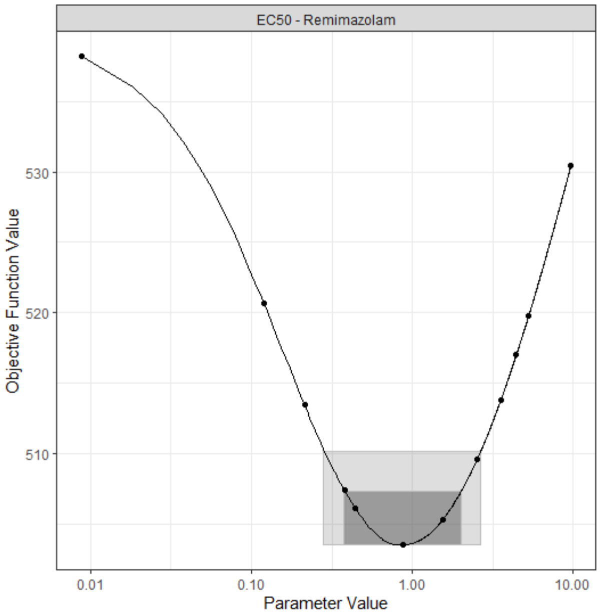

Model code – pharmacodynamic model (TOTS)

```
$PROB   ….
$INPUT NMID=ID SESS RTIME DVTY AMT RATE EVID DV MDV BLQ AGE HEIGHT WEIGHT BMI SEX
CMT TIME IV1 IV2 IV3 ICL IQ2 IQ3 ICLM IV5 IKTR IIC50 IGAM IV1R IV2R IV3R ICLR IQ2R IQ3R
$DATA ….
$SUBROUTINES ADVAN6 TOL=9
$MODEL
COMP=(CENTRAL)
COMP=(PERIPHERAL1)
COMP=(PERIPHERAL2)
COMP=(TRANSIT)
COMP=(METABOLITE)
COMP=(REMIFENTANIL)
COMP=(PERIREM1)
COMP=(PERIREM2)

;Pharmacokinetics
$PK (ONCE PER IR)
        ;Parameters:
                ;Structural - Parent:
                V1 = IV1
                V2 = IV2
                V3 = IV3
                CL = ICL
                Q2 = IQ2
                Q3 = IQ3

                ;Structural - Metabolite:
                CLM = ICLM
```

```
V5 = IV5
KTR = IKTR

;Remifentanil model (Eleveld Model)
        V1R = IV1R
        V2R = IV2R
        V3R = IV3R
        CLR = ICLR
        Q2R = IQ2R
        Q3R = IQ3R

;Scaling Parameters
        ;Parent:
        S1 = V1/1000
        FM = 0.8

        ;Metabolite:
        S5 = V5/1000

        ;Remifentanil:
        S6 = V1R

;Rate constants:
        ;Remimazolam:
        K10 = (CL*(1-FM))/V1
        K12 = Q2/V1
        K21 = Q2/V2
        K13 = Q3/V1
        K31 = Q3/V3
```

```
			;Metabolite:
			K14 = (CL*FM)/V1
			K50 = CLM/V5
			K54 = KTR

			;Remifentanil:
			K60 = CLR/V1R
			K67 = Q2R/V1R
			K76 = Q2R/V2R
			K68 = Q3R/V1R
			K86 = Q3R/V3R

		;Interaction
		IC50 = IIC50
		GAM = IGAM
;TOTS
		;Structural
		EC50T = EXP(THETA(1)+ETA(1))
		EC50TR = EXP(THETA(2))
;Differential Equations:
$DES
CONC = A(6)/V1R
INH = 0
IF(CONC.GT.0) INH = CONC**GAM / (CONC**GAM + IC50**GAM)

;Remimazolam:
DADT(1) = -K10*A(1) - K12*A(1) + K21*A(2) - K13*A(1) + K31*A(3) - K14*A(1)
DADT(2) =           K12*A(1) - K21*A(2)
```

```
DADT(3) =                                     K13*A(1) - K31*A(3)

;Metabolite:
DADT(4) =                                                         K14*A(1) - K54*A(4)
DADT(5) = -K50*A(5)*(1 - INH)                                                 + K54*A(4)

;Remifentanil:
DADT(6) = -K60*A(6) - K67*A(6) + K76*A(7) - K68*A(6) + K86*A(8)
DADT(7) =               K67*A(6) - K76*A(7)
DADT(8) =                                     K68*A(6) - K86*A(8)

$ERROR
;Redefine variables in DES:
CP = A(1)/V1*1000
RATIO = 425.3/439.3                  ;MW[Metabolite]425.3 / MW[Parent] 439.3
CM = A(5)/V5*1000*RATIO
CR = A(6)/V1R

;TOTS:
        ;Structural:
        CA = 0
        CB = 0
        DRUGB = 0
        IF(CP.GT.0) CA = CP/(EC50T*(1 - CR/(CR + EC50TR)))
        IF(CM.GT.0) CB = 0
        IF(CP.GT.0) DRUGT = CA/(1 + CA + CB)

        ;Mapping to data:
        P0 = 1 * (1 - DRUGT)
```

```
        IF(DVTY.EQ.7.AND.DV.EQ.0) Y = P0

        IF(DVTY.EQ.7.AND.DV.EQ.1) Y = 1 - P0


$THETA

7.8             ;EC50T

-0.3            ;EC50TR


$OMEGA

0.1             ;ETA EC50T

$ESTM METHOD=1 LAPLACIAN LIKELIHOOD NUMERICAL SLOW NOABORT POSTHOC PRINT=1
MAXEVAL=9999

$TABLE ….
```
