# Supplementary material for: Influence of Remifentanil on the Pharmacokinetics and Pharmacodynamics of Remimazolam in Healthy Volunteers
Source: Anesthesiology. 2025 Jan 15;142(4):666–79. doi: 10.1097/ALN.0000000000005348 (PMC11892992; doi:10.1097/ALN.0000000000005348)

Supplemental Digital Content 7

Table 1. Predicted relationship between remimazolam target concentration and Bispectral Index, Modified Observer's Assessment of Alertness and Sedation Scores, tolerance to tetanic stimulation and tolerance to laryngoscopy stratified by remifentanil concentration after 60 minutes of target controlled infusion of remimazolam.

Remifentanil target concentration (ng/mL)	Remimazolam target concentration (ng/mL)	BIS	TOL (%)	TOTS (%)	MOAAS 5 (%)	MOAAS 4 (%)	MOAAS 3 (%)	MOAAS 2 (%)	MOAAS 1 (%)	MOAAS 0 (%)
0	150	78	0.3	1.2	50.0	40.4	5.2	2.8	1.3	0.2
	300	68	0.6	2.4	5.6	30.2	20.6	23.0	17.1	3.4
	400	63	0.7	3.2	1.9	13.7	14.4	26.1	34.2	9.6
	800	53	1.5	6.2	0.3	2.2	3.1	9.4	41.3	43.7
	1300	48	2.3	9.7	0.1	1.0	1.4	4.6	28.7	64.2
	2000	45	3.6	14.2	0.1	0.6	0.9	2.9	21.1	74.4
0.1	150	78	1.5	4.1	46.7	42.5	5.8	3.2	1.5	0.2
	300	68	3.0	7.8	4.4	26.1	20.0	24.8	20.4	4.3
	400	63	4.0	10.1	1.5	10.8	12.2	24.7	38.5	12.4
	800	53	7.6	18.4	0.2	1.6	2.2	7.0	36.4	52.7
	1300	49	11.8	26.8	0.1	0.7	1.0	3.2	22.4	72.7
	2000	45	17.1	36.0	0.0	0.4	0.6	2.0	15.5	81.5
0.5	150	77	6.2	13.9	33.7	49.1	9.0	5.3	2.5	0.4
	300	67	11.7	24.4	1.7	12.4	13.5	25.6	36.0	10.7
	400	63	15.0	30.1	0.5	3.8	5.1	14.1	45.7	30.9
	800	53	26.2	46.3	0.0	0.4	0.5	1.8	14.4	82.8
	1300	48	36.5	58.3	0.0	0.1	0.2	0.7	6.0	93.0
	2000	45	46.9	68.3	0.0	0.1	0.1	0.4	3.5	96.0
1	150	76	11.5	23.7	20.4	50.4	14.1	9.4	4.8	0.8
	300	66	20.6	38.3	0.5	4.1	5.5	15.0	45.9	29.0
	400	62	25.8	45.3	0.1	0.9	1.3	4.4	27.8	65.5
	800	53	41.0	62.4	0.0	0.1	0.1	0.3	2.7	96.8
	1300	48	53.0	72.9	0.0	0.0	0.0	0.1	0.9	99.0
	2000	45	63.4	80.6	0.0	0.0	0.0	0.0	0.4	99.5
2	150	74	20.5	37.9	6.2	32.1	20.8	22.1	15.8	3.1
	300	65	34.0	54.9	0.0	0.4	0.5	1.8	14.5	82.7
	400	61	40.7	61.9	0.0	0.1	0.1	0.3	2.4	97.2
	800	53	57.9	76.5	0.0	0.0	0.0	0.0	0.1	99.9
	1300	48	69.0	84.1	0.0	0.0	0.0	0.0	0.0	100.0
	2000	45	77.4	89.0	0.0	0.0	0.0	0.0	0.0	100.0
4	150	70	33.8	54.7	0.4	3.5	4.7	13.3	45.4	32.8
	300	62	50.6	70.7	0.0	0.0	0.0	0.0	0.1	99.8
	400	59	57.7	76.3	0.0	0.0	0.0	0.0	0.0	100.0
	800	52	73.2	86.6	0.0	0.0	0.0	0.0	0.0	100.0
	1300	48	81.6	91.3	0.0	0.0	0.0	0.0	0.0	100.0
	2000	45	87.2	94.1	0.0	0.0	0.0	0.0	0.0	100.0

Figure 1. Predicted relationship between remimazolam target concentration and Bispectral Index, Modified Observer's Assessment of Alertness and Sedation Scores, tolerance to tetanic stimulation and tolerance to laryngoscopy stratified by remifentanil target concentration after 60 minutes of target controlled infusion of remimazolam.

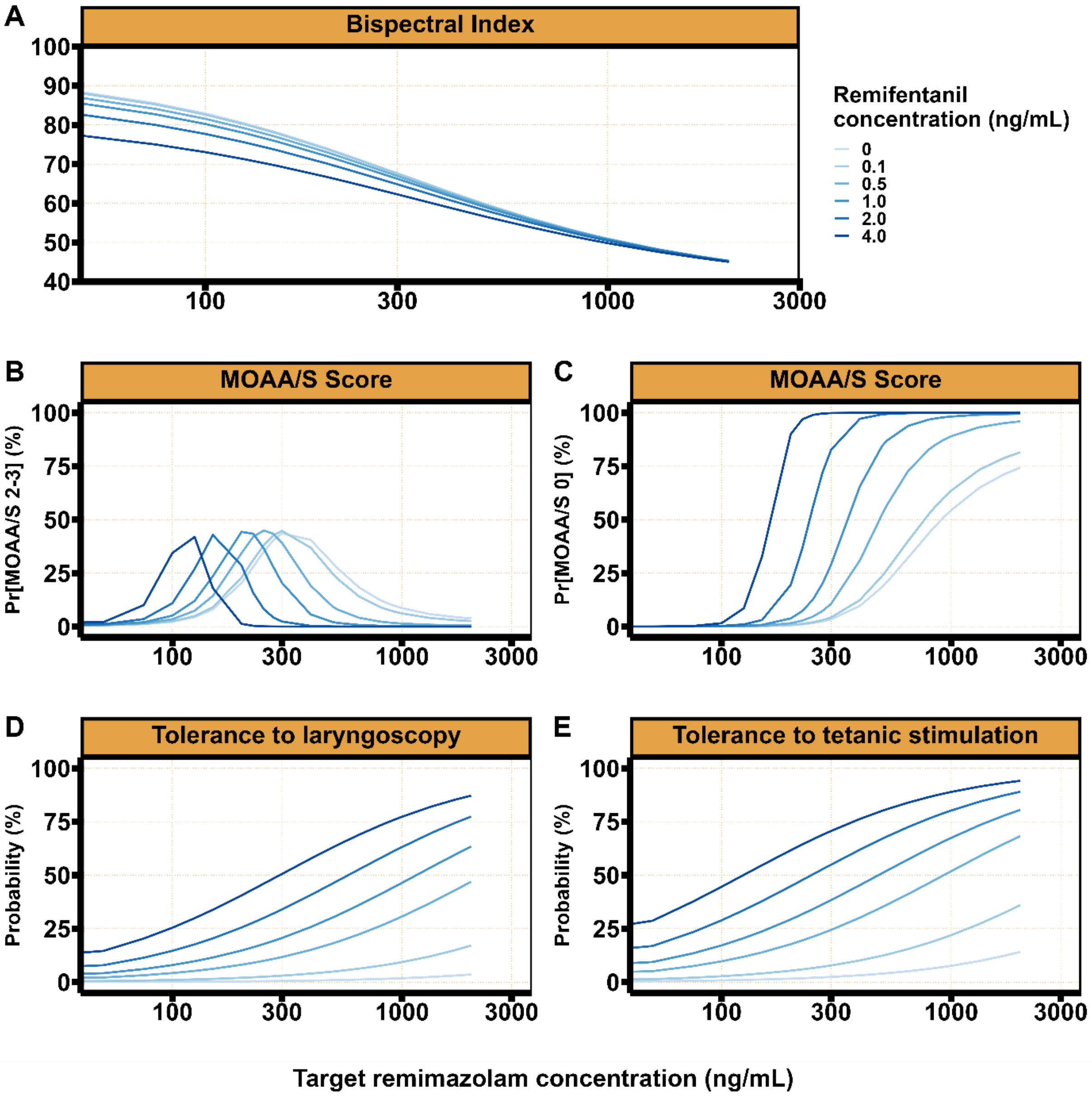

Table 2. Predicted relationship between remimazolam target concentration and Bispectral Index, Modified Observer's Assessment of Alertness and Sedation Scores, tolerance to tetanic stimulation and tolerance to laryngoscopy stratified by remifentanil concentration after 120 minutes of target controlled infusion of remimazolam.

Remifentanil target concentration (ng/mL)	Remimazolam target concentration (ng/mL)	BIS	TOL (%)	TOTS (%)	MOAAS 5 (%)	MOAAS 4 (%)	MOAAS 3 (%)	MOAAS 2 (%)	MOAAS 1 (%)	MOAAS 0 (%)
0	150	79	0.3	1.2	58.1	34.8	3.9	2.1	0.9	0.2
	300	70	0.6	2.4	10.0	41.2	19.7	17.0	10.3	1.8
	400	66	0.7	3.2	3.8	23.4	19.3	25.7	22.9	5.0
	800	57	1.5	6.2	0.5	4.3	5.7	15.5	46.0	28.0
	1300	53	2.4	9.7	0.2	1.7	2.5	7.7	38.0	49.9
	2000	50	3.6	14.2	0.1	1.0	1.4	4.6	28.7	64.3
0.1	150	79	1.5	4.1	55.4	36.8	4.3	2.3	1.0	0.2
	300	69	3.0	7.8	8.3	37.9	20.4	19.0	12.1	2.2
	400	66	4.0	10.1	3.0	19.8	17.9	26.5	26.5	6.2
	800	57	7.6	18.4	0.4	3.2	4.4	12.5	44.9	34.6
	1300	53	11.8	26.8	0.1	1.2	1.8	5.7	32.6	58.7
	2000	50	17.1	36.0	0.1	0.7	1.0	3.2	22.6	72.5
0.5	150	78	6.2	13.9	43.6	44.4	6.5	3.6	1.7	0.3
	300	69	11.7	24.4	3.8	23.2	19.2	25.7	23.0	5.1
	400	65	15.1	30.1	1.1	8.7	10.4	22.8	41.7	15.3
	800	57	26.2	46.3	0.1	0.9	1.3	4.2	27.1	66.4
	1300	53	36.5	58.4	0.0	0.3	0.4	1.4	11.7	86.1
	2000	50	47.0	68.3	0.0	0.1	0.2	0.7	6.2	92.8
1	150	77	11.5	23.7	29.8	50.3	10.3	6.2	3.0	0.5
	300	68	20.6	38.4	1.3	9.9	11.5	24.0	39.8	13.5
	400	65	25.8	45.4	0.3	2.6	3.7	10.9	43.3	39.2
	800	57	41.0	62.4	0.0	0.2	0.2	0.9	7.5	91.2
	1300	53	53.0	72.9	0.0	0.0	0.1	0.2	2.1	97.5
	2000	51	63.4	80.6	0.0	0.0	0.0	0.1	0.9	98.9
2	150	75	20.5	37.9	11.3	43.3	19.0	15.6	9.1	1.6
	300	67	34.0	54.9	0.2	1.3	1.9	6.0	33.6	57.1
	400	64	40.7	61.9	0.0	0.2	0.3	1.1	9.3	89.1
	800	57	57.9	76.5	0.0	0.0	0.0	0.0	0.3	99.7
	1300	53	69.0	84.1	0.0	0.0	0.0	0.0	0.1	99.9
	2000	51	77.4	89.0	0.0	0.0	0.0	0.0	0.0	100.0
4	150	71	33.9	54.7	1.1	8.7	10.4	22.9	41.6	15.2
	300	65	50.6	70.7	0.0	0.0	0.0	0.1	0.9	99.0
	400	62	57.7	76.3	0.0	0.0	0.0	0.0	0.1	99.9
	800	56	73.2	86.6	0.0	0.0	0.0	0.0	0.0	100.0
	1300	53	81.6	91.3	0.0	0.0	0.0	0.0	0.0	100.0
	2000	51	87.2	94.1	0.0	0.0	0.0	0.0	0.0	100.0

Figure 2. Predicted relationship between remimazolam target concentration and Bispectral Index, Modified Observer's Assessment of Alertness and Sedation Scores, tolerance to tetanic stimulation and tolerance to laryngoscopy stratified by remifentanil target concentration after 120 minutes of target controlled infusion of remimazolam.

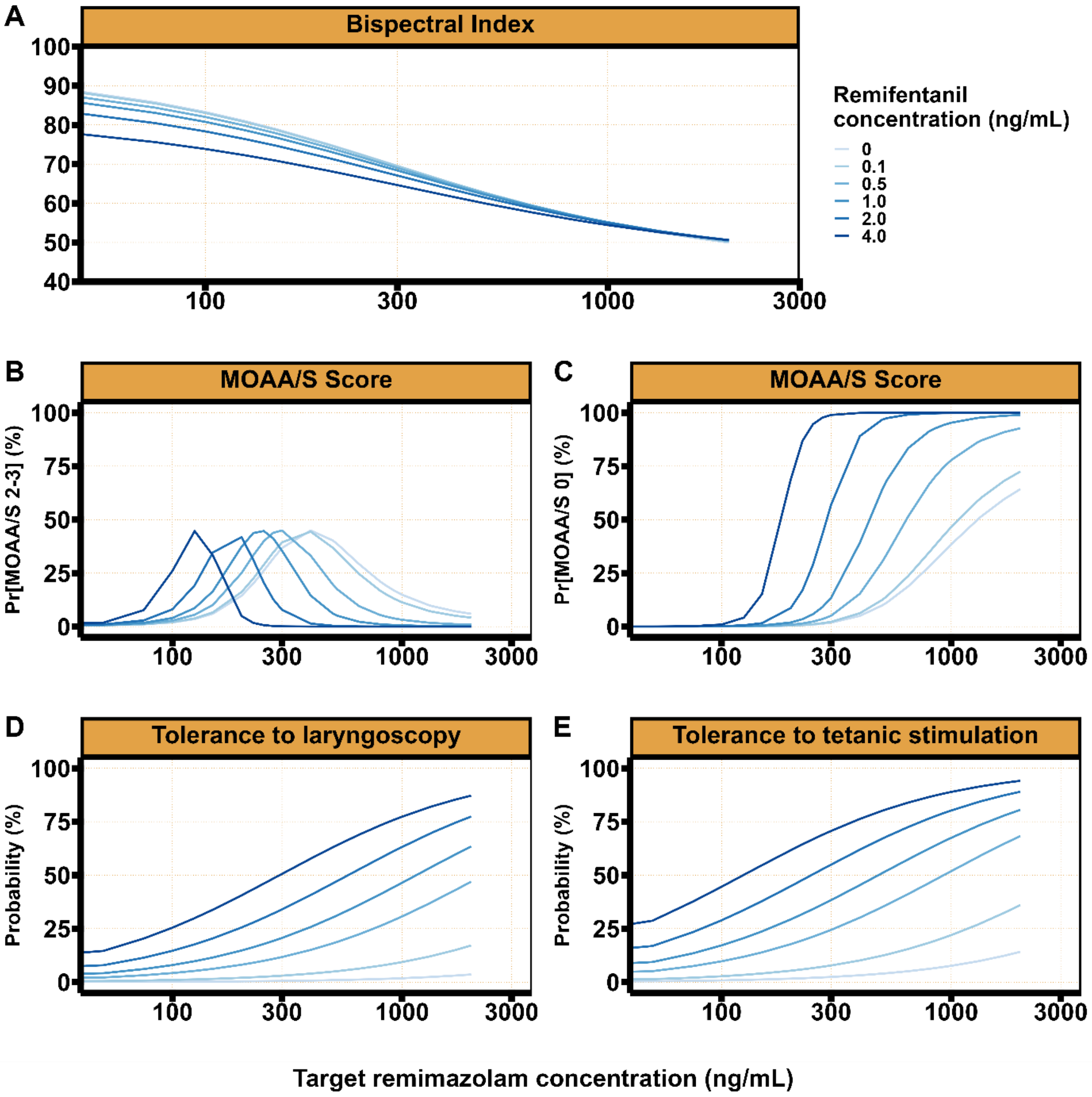

Supplement: Supplementary file 7 [file aln-142-666-s007.pdf]
